# Additive effects of fecal microbiota transplantation and infliximab on gut microbiome and metabolome in refractory inflammatory bowel disease patients

**DOI:** 10.1128/msystems.00774-25

**Published:** 2026-03-23

**Authors:** Xinjun Wang, Weijun Wu, Bo Yang, Yunkun Liu, Yue Xu, Le Wang, Xiaoqiong Lv, Junhui Gao, Man Lu, Anqi Yu, Ning Li, Qiyi Chen, Liesheng Lu, Di Zhao

**Affiliations:** 1 Department of Functional Intestinal Diseases, General Surgery of Shanghai Tenth People's Hospital, Tongji University School of Medicine 481875, Shanghai, China; 2 Affiliated Suzhou Hospital of Nanjing Medical University, Suzhou Municipal Hospital, Gusu School, Nanjing Medical University 117958 , Suzhou, China; 3 Shanghai Gastrointestinal Microecology Research Center, Shanghai, China; 4 Shanghai Institution of Gut Microbiota Research and Engineering Development, Shanghai, China; 5 Clinical Research Center for Digestive Diseases, Tongji University School of Medicine 481875, Shanghai, China; 6 Shanghai Zhangjiang Institute of Medical Innovation, Shanghai, China; 7 Department of Pathology, Shanghai Tenth People’s Hospital, Shanghai, China; 8 Department of Gastrointestinal Surgery, General Surgery of Shanghai Tenth People's Hospital, Tongji University School of Medicine 481875, Shanghai, China; Purdue University, West Lafayette, Indiana, USA

**Keywords:** inflammatory bowel disease, fecal microbiota transplantation, infliximab, microbiome, metabolome

## Abstract

Fecal microbiota transplantation (FMT) is an emerging therapy for inflammatory bowel disease (IBD), yet its efficacy in patients refractory to conventional treatments and its underlying mechanisms require further elucidation. We studied 37 IBD patients (15 ulcerative colitis [UC], 22 Crohn’s disease [CD]) refractory to conventional therapies and 16 healthy donors. FMT monotherapy from a single donor induced week-4 clinical response in 12 UC and 9 biologic-naïve CD patients, with all responders sustaining remission and most achieving endoscopic remission by week 14. Integrated multi-omics revealed FMT restored microbial diversity and profoundly reorganized host-microbiota-metabolite networks. In nine refractory CD patients (7 infliximab [IFX] non-responders, 2 FMT non-responders), IFX-FMT combination led to week-4 response in 6 patients, all of whom attained clinical and endoscopic remission by week 14, with more complete microbial-metabolic restoration than monotherapy. Our findings establish that FMT induces remission in refractory IBD via ecosystem network rewiring, and that IFX-FMT exhibits additive effects, supporting further trials of microbiome-directed adjunctive strategies.

**IMPORTANCE:**

This study provides mechanistic and clinical insights into the therapeutic effects of fecal microbiota transplantation (FMT) in inflammatory bowel disease (IBD), particularly when combined with the anti-tumor necrosis factor (anti-TNF) biologic infliximab (IFX). While both FMT and IFX achieve response in approximately 60% of IBD patients, their combined influence on the gut microbial and metabolic landscape in refractory disease has been poorly understood. Here, we demonstrate that FMT monotherapy restores gut microbial diversity and reconfigures host-microbiota-metabolite networks, correlating with clinical and endoscopic remission in patients refractory to conventional treatments. Furthermore, in Crohn's disease patients unresponsive to either therapy alone, combined IFX-FMT induced more complete microbial and metabolic normalization and achieved remission where monotherapy had failed. These findings reveal ecosystem-level network rewiring as a central mechanism of FMT efficacy and establish the additive potential of combining microbiome-targeted and immunomodulatory therapies. This work supports the development of microbiome-informed adjunctive strategies for severe or refractory IBD, highlighting an actionable path toward personalized, mechanism-based treatment regimens.

**CLINICAL TRIALS:**

This study is registered with ClinicalTrials.gov as NCT07149441.

## INTRODUCTION

Inflammatory bowel disease (IBD), encompassing ulcerative colitis (UC) and Crohn’s disease (CD), is characterized by chronic intestinal inflammation driven by immune dysregulation and gut microbiota dysbiosis (1–6). Anti-tumor necrosis factor (anti-TNF) therapies, such as infliximab (IFX), achieve clinical response in approximately 60% of IBD patients by neutralizing TNFα and mitigating inflammation (7, 8). However, primary or secondary non-response, often linked to persistent dysbiosis, limits efficacy in a significant proportion of patients, particularly those with refractory CD who fail initial IFX therapy (4, 9, 10). Fecal microbiota transplantation (FMT), which restores gut microbial balance, has emerged as a promising treatment for IBD, with a response rate of ~60% in UC and CD, especially in early-stage disease or with *Clostridium difficile* infection (11–17). Despite its efficacy, FMT’s role as an adjunct to anti-TNF therapies remains underexplored.

The gut microbiota influences IBD pathogenesis through host-microbe co-metabolism, involving short-chain fatty acids (SCFAs), bile acids, and amino acids (18–24). Dysregulated metabolites, such as reduced SCFAs and elevated bile acids, exacerbate inflammation and impair therapeutic outcomes (19, 23). FMT modulates these pathways, enhancing microbial diversity and metabolic profiles in responders (25), while IFX increases diversity in some patients (9). However, the additive effects of FMT and IFX on the gut microbiome and metabolome, especially in patients refractory to conventional treatments, are poorly understood. Such combined effects could improve outcomes for patients unresponsive to standard therapies.

This study evaluates (i) the impact of FMT monotherapy on the gut microbiome and metabolome in UC and biologic-naive CD patients, using a fixed, individual donor per patient for consistent omics analyses, and (ii) the additive potential of IFX-FMT combination therapy in CD patients. By integrating 16S rRNA sequencing, liquid chromatography-tandem mass spectrometry (LC-MS/MS)-based metabolomics, and clinical assessments at weeks 4 and 14, we aim to elucidate microbial and metabolic mechanisms underlying treatment responses, providing insights into personalized IBD therapeutic strategies.

## MATERIALS AND METHODS

### Characteristics of study cohort

We recruited 53 participants from Shanghai Tenth People’s Hospital, including 30 IFX-naïve IBD patients (15 UC, 15 CD) who failed conventional therapy and 16 healthy FMT donors (see Table S1). UC was diagnosed based on persistent diarrhea, mucinous bloody stool, abdominal pain, and positive colonoscopy/biopsy results. CD was diagnosed based on diarrhea, abdominal pain, fistula formation, and positive colonoscopy/biopsy findings. Healthy donors (aged 20–30 years) underwent stool tests, 16S rDNA sequencing, and multidrug resistance screening, adhering to the FMT International Consensus (26). IBD patients (aged 15–70 years) followed an anti-inflammatory diet (27) supplemented with partial enteral nutrition, monitored via weekly surveys to minimize dietary variability. The seven patients with primary non-response to infliximab met the criteria for refractory Crohn’s disease, defined as failure to respond to corticosteroids and/or mesalazine, lack of induction of remission after a prior course of another biologic agent, and persistent disease activity (Crohn’s disease activity index [CDAI] decrease <100 points or ongoing clinical activity) despite completion of standard infliximab induction therapy (5 mg/kg at weeks 0, 2, and 6).

### FMT and IFX treatment protocols

FMT monotherapy was administered to 30 patients (15 UC, 15 CD). Donor feces (100 g) were transferred to a sterile commercial blender bag under ambient atmospheric conditions and homogenized with 300 mL of sterile 0.9% saline (1:3 wt/vol) using a stomacher (3 × 60 s cycles at high speed). The homogenate was sequentially filtered through sterile stainless-steel sieves (2.0 mm, followed by 0.5 mm pore size) to remove undigested particulate matter. Sterile glycerol was then added to a final concentration of 10% (vol/vol) as a cryoprotectant. Aliquots (50–60 mL) were immediately transferred to sterile 100-mL screw-cap polypropylene containers and frozen at −80°C (not −20°C) for storage of 1–8 weeks, in accordance with international FMT consensus guidelines and published evidence demonstrating superior preservation of anaerobic taxa at −80°C.

Recipient preparation consisted of a 6-day oral vancomycin pretreatment (500 mg twice daily) to reduce resident microbiota, followed by bowel cleansing with 2–3 L of polyethylene glycol solution 12–24 h prior to the first transplant. On the day of administration, aliquots were thawed in a 37°C water bath for 20–25 min under continuous gentle agitation. Viability checks (optional live/dead staining or CFU plating on selective media) confirmed <15% loss of anaerobic bacteria post-thawing. The suspension was drawn into 60-mL catheter-tip syringes and administered within 30 min of thawing via a pre-placed nasojejunal tube (Flocare or equivalent), with the tip positioned fluoroscopically or endoscopically in the proximal jejunum. A total volume of 100 mL was instilled daily over 5–10 min for six consecutive days, delivering an estimated 10¹¹–10¹² viable microorganisms per transplant (13, 28). The single-donor approach facilitated direct pre- and post-treatment comparisons of microbiome and metabolome profiles between donor and recipient, consistent with clinical FMT practices for IBD (26, 28). Nine CD patients received IFX-FMT combination therapy. Primary IFX non-response was defined as persistent disease activity (CDAI > 150) after standard IFX induction (5 mg/kg at weeks 0, 2, and 6). For the seven IFX non-responders, combination therapy consisted of continued IFX maintenance dosing (5 mg/kg every 8 weeks) plus a single FMT. For the two FMT non-responders, combination therapy involved IFX induction (5 mg/kg at weeks 0, 2, 6, and week 14) with a second FMT from the same donor.

### Disease assessment

Clinical response was assessed at week 4: UC patients required a partial Mayo score reduction of ≥2 points and a rectal bleeding subscore decrease of ≥1 point; CD patients required a CDAI decrease of ≥100 points. At week 14, clinical remission (UC: partial Mayo score < 3; CD: CDAI < 150) and endoscopic remission (UC: Mayo endoscopic subscore ≤ 1; CD: simplified endoscopic score for Crohn’s disease [SES-CD] ≤ 2) were evaluated via colonoscopy.

### Sample collection and analysis

Fecal samples were collected from donors and patients per Integrative Human Microbiome Project protocols (23). For patients receiving FMT monotherapy (15 UC, 15 CD), samples were collected pre-treatment (baseline) and 28 days post-FMT to assess microbial and metabolomic changes. For the nine CD patients receiving IFX-FMT combination therapy, sample collection varied by subgroup: (i) for the seven IFX non-responders, samples were collected before the single FMT and 28 days post-FMT; (ii) for the two FMT non-responders, samples were collected before the second FMT and 28 days after the second FMT while receiving IFX induction therapy. Fresh samples were used for calprotectin measurement and 16S rDNA sequencing, with remaining samples stored at −80°C for LC-MS-based metabolomics (custom panel per Zhao et al. [18]). Microbial α-diversity (ACE, Chao1, Simpson) and β-diversity were analyzed. Metabolites were screened for FMT response using three criteria: (i) differential abundance in baseline versus donors, (ii) shift to donor levels in responders, and (iii) persistence of baseline levels in non-responders.

### Metabolomic experiments

Fecal samples were thawed in an ice bath to minimize degradation. Approximately 5 mg of the lyophilized sample was weighed and transferred to a 1.5 mL tube. The sample was reconstituted with 25 μL of deionized water and homogenized with zirconium oxide beads for 3 min. Metabolites were extracted by adding 120 μL of methanol containing an internal standard, followed by an additional 3 min of homogenization. The mixture was centrifuged at 18,000 × *g* for 20 min at 4°C, and 20 μL of the supernatant was transferred to a 96-well plate. Subsequent steps were automated using an Eppendorf epMotion Workstation (Eppendorf Inc., Hamburg, Germany). Each well received 20 μL of freshly prepared derivatization reagents, and the plate was sealed and incubated at 30°C for 60 min. Post-derivatization, 330 μL of ice-cold 50% methanol was added to dilute the sample. The plate was stored at −20°C for 20 min and centrifuged at 4,000 × *g* for 30 min at 4°C. Then, 135 μL of the supernatant was transferred to a new 96-well plate, supplemented with 10 μL of internal standards per well. Serial dilutions of derivatized stock standards were added to the remaining wells. The plate was sealed and analyzed by LC-MS/MS with a 5 μL injection volume. Metabolite quantification was performed using a UPLC-MS/MS system (ACQUITY UPLC-Xevo TQ-S, Waters Corp., Milford, MA, USA) equipped with an ACQUITY UPLC BEH C18 1.7 μm column (2.1 × 100 mm). Mobile phase A was deionized water with 0.1% formic acid, and mobile phase B was acetonitrile with 30% isopropanol. The gradient was as follows: 0 min, 5% B; 1 min, 5% B; 11 min, 78% B; 13.5 min, 95% B; 14 min, 100% B; 16 min, 100% B; 16.1 min, 5% B; 18 min, 5% B, at a flow rate of 0.4 mL/min. Mass spectrometry conditions included capillary voltages of 1.5 kV (ESI+) and 2.0 kV (ESI−), source temperature of 150°C, desolvation temperature of 550°C, and desolvation gas flow of 1,000 L/h.

### Metabolome data analysis

Raw LC-MS/MS data were processed using MassLynx software (v4.1, Waters Corp., Milford, MA, USA) for peak integration, calibration, and metabolite quantification. Metabolite concentrations were determined by comparing sample responses to calibration curves generated from standard samples with known concentrations. Calibration curves were constructed by plotting instrument response (peak area) against analyte concentration, following the linear equation y = ax + b, where y is the response, a is the slope (sensitivity), b is the background, and x is the concentration. Statistical analyses were conducted using R Studio (v4.2.3). Data were normalized, and multivariate analyses, including principal component analysis (PCA), were performed to identify patterns in metabolite profiles.

### Microbiome experiments

Fresh fecal samples were collected in sterile tubes (Fisher Scientific, Waltham, MA, USA) and stored at −80°C. Microbial DNA was extracted from 200 mg of each sample using the QIAamp PowerFecal Pro DNA Kit (QIAGEN, Hilden, Germany), which includes a bead-beating step. Briefly, 200 mg of the sample was mixed with 800 μL of lysis buffer and vortexed with beads at maximum speed for 10 min. After centrifugation at 15,000 × *g* for 1 min, 350 μL of supernatant was processed per kit instructions. DNA was eluted in 100 μL of elution buffer. The V4 region of the 16S rRNA gene was amplified using primers 515F (5′-GTGYCAGCMGCCGCGGTA-3′) and 806R (5′-GGACTACNVGGGTWTCTAAT-3′) in a Veriti 96-Well Thermal Cycler (Thermo Fisher Scientific, Waltham, MA, USA). The PCR program consisted of 95°C for 3 min, 21 cycles of 95°C for 30 s, 56°C for 30 s, and 72°C for 30 s, followed by 72°C for 5 min. Amplicons were pooled and sequenced on an Illumina NovaSeq 6000 (Illumina, San Diego, CA, USA) at Shanghai Biotecan Pharmaceuticals Co., Ltd. (Shanghai, China), per manufacturer protocols.

### Microbiome data analysis

Sequences were clustered at 97% similarity using the Greengenes database (v13_8) in mothur (v1.39.5). Taxonomy was assigned with Greengenes for comparative analysis. Functional pathways were predicted using PICRUSt and visualized in STAMP (v2.1.3). Linear discriminant analysis (LDA) effect size was performed (http://huttenhower.sph.harvard.edu/galaxy) to identify differentially abundant taxa, with a cutoff of LDA score (log10) >3.0 and *P* < 0.05. Alpha diversity indices (ACE, Chao1, Shannon, Simpson) were calculated using mothur’s “summary.single” script. Co-abundance networks were constructed based on Spearman correlations, including genera present in ≥60% of samples with absolute correlation coefficients |r| >0.6 and *P* < 0.05, using the Igraph R package and visualized in Cytoscape (v3.6.0).

### Statistical analysis

Statistical analyses were performed using R Studio (http://cran.r-project.org/). Metabolomic and microbiome data underwent multivariate analyses, including PCA and partial least squares discriminant analysis (PLS-DA), and univariate analyses, such as Student’s *t*-test, Mann-Whitney U-test, and ANOVA. In PCA and PLS-DA, samples were represented in K-dimensional space, color-coded by group, with R²X and R²Y indicating variance explained by the X and Y matrices, respectively, and Q²Y reflecting predictive accuracy. Models with R²X, R²Y, and Q²Y close to 1.0 were considered robust. Variables with a variable importance in the projection score >0.9 were deemed significantly different between groups. Spearman correlation analysis was used for network construction, with *P* < 0.05 considered significant.

## RESULTS

### FMT monotherapy induces clinical and endoscopic remission in patients failing conventional therapy

**Fig 1:**
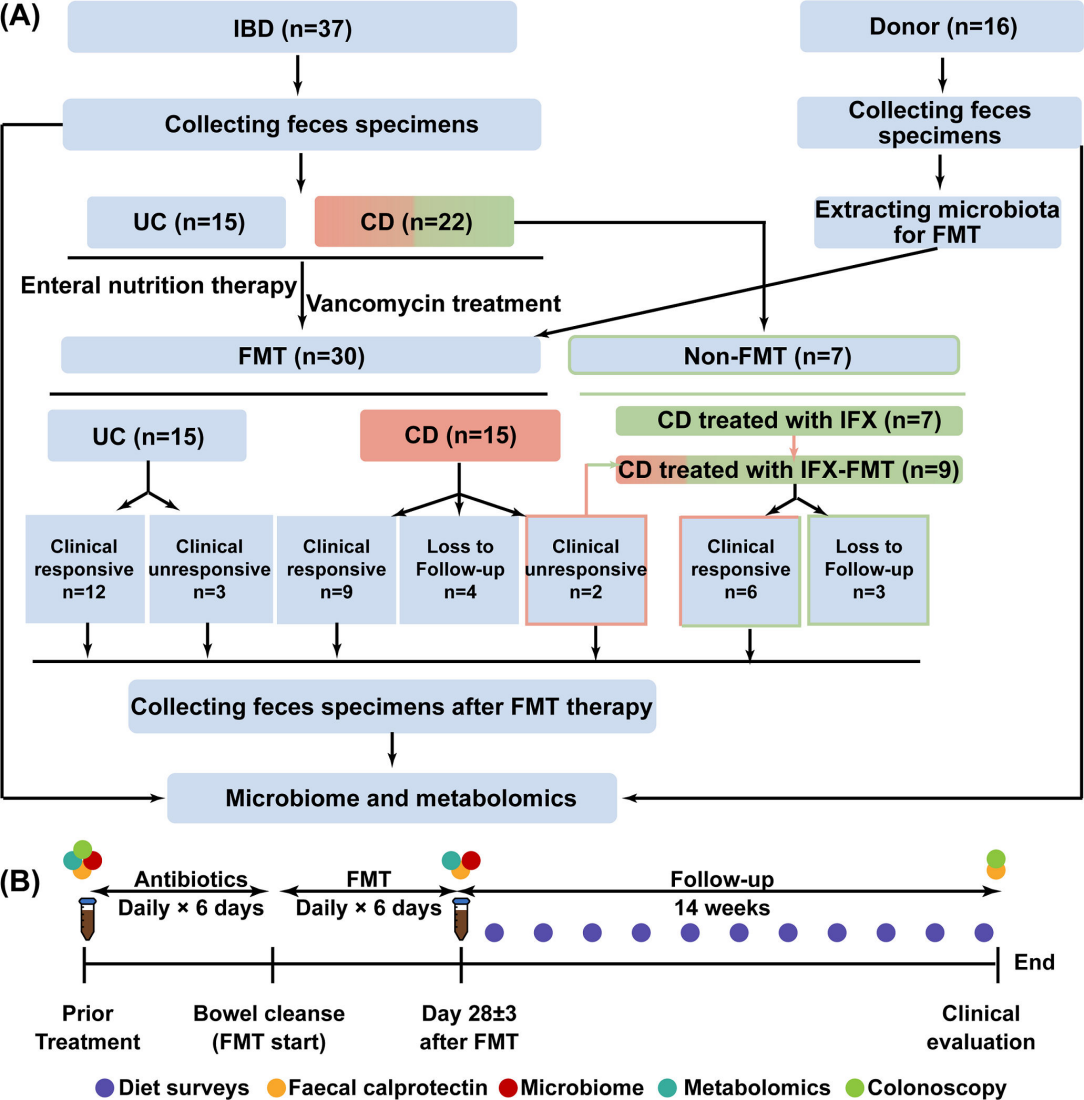
Cohort characteristics and study protocol. (**A**) Cohort schema illustrating the integration of microbiome and metabolomics analyses to identify microbe-metabolite indicators associated with FMT efficacy in IBD patients. (**B**) Sample collection strategy for microbiome and metabolome analysis. Legend: *n,* number of patients; IFX, infliximab.

We enrolled 37 IBD patients (15 UC, 22 CD) who had failed conventional therapies (mesalazine, corticosteroids, and/or immunomodulators) and 16 healthy FMT donors (Fig. 1A; Table S1). Donors (aged 20–30 years) met FMT International Consensus criteria (26). Thirty biologic-naïve patients (15 UC, 15 CD) received FMT monotherapy using a single fixed donor per patient.

In UC, 12 of 15 patients achieved clinical response at week 4, and all 12 maintained clinical remission at week 14, with 8 achieving endoscopic remission (Table 1). In biologic-naïve CD, 9 of 15 patients achieved clinical response at week 4, and all 9 sustained clinical remission at week 14, with 8 achieving endoscopic remission (Table 1).

**TABLE 1:** Outcomes at 4 and 14 weeks in UC and CD patients

Patient	Clinical outcome by week 4			Clinical outcome by week 14			
	CRP (mg/dL)	Calprotectin protein (μg/g)	Clinical response	CRP (mg/dL)	Calprotectin protein (μg/g)	Clinical remission	Endoscopic remission
UC 1	8.21	25.2	R	2.55	15.4	R	R
UC 2	10.2	79.6	R	5.5	54.2	R	R
UC 3	7.5	15	R	10.1	23	R	R
UC 4	3.2	117.9	R	3.04	72	R	NR
UC 5	7.5	342.9	R	4.1	98	R	NR
UC 6	8.2	15	R	2.9	23.4	R	R
UC 7	4.6	83.4	R	5.3	73.8	R	NR
UC 8	6.3	15	R	5.6	18	R	R
UC 9	10.0	41.5	R	8.7	15.4	R	R
UC 10	4.3	29.9	R	2.26	37.5	R	R
UC 11	5.3	93.2	R	2.03	123.9	R	NR
UC 12	3.9	15	R	5.13	43	R	R
UC 13	24.8	861.7	NR	7.3	362.7	NR	NR
UC 14	15.5	125.9	NR	2.3	231.9	NR	NR
UC 15	31.1	261.7	NR	6.86	321.6	NR	NR
CD 1	2.2	34.6	R	4.0	36.4	R	R
CD 2	3.08	62.4	R	9.7	42.6	R	R
CD 3	5.2	23.8	R	3.32	28.3	R	R
CD 4	4.9	53.3	R	7.1	33.5	R	R
CD 5	4.2	16.7	R	5.22	37.6	R	R
CD 6	3.8	20.4	R	6.55	24.0	R	R
CD 7	5.5	19.7	R	5.03	17	R	R
CD 8	6.3	49.1	R	3.10	19.4	R	R
CD 9	15.8	117.1	R	2.3	231.5	R	NR
CD 10	4.74	26.9	R	10.9	33.5	R	R
CD 11	6.3	120.8	R	20.1	54.2	R	R
IFX 1	3.6	129.7	R	2.08	72	R	R
IFX 2	7.02	29.6	R	3.02	41.2	R	R
IFX 3	5.1	66.6	R	4.23	52.3	R	R
IFX 4	3.2	104.8	R	3.32	19.2	R	R

Notably, even in patients who had failed either FMT or infliximab monotherapy, subsequent combined IFX-FMT therapy induced rapid clinical improvement (marked reductions in CRP and fecal calprotectin) and endoscopic healing by week 14 (Fig. 4), suggesting potential additive effects that we explore further below.

These results demonstrate that FMT monotherapy can induce substantial clinical and endoscopic remission in IBD patients refractory to conventional therapies, laying the foundation for understanding its mechanistic basis and potential combined effects with infliximab.

### FMT remission is driven by restoration of microbial diversity and reorganization of host-microbiota-metabolite interaction networks

In both UC and biologic-naïve CD patients receiving FMT monotherapy, clinical responders exhibited consistent patterns of microbial and metabolomic restoration toward donor-like profiles. Key shared mechanisms included increased α-diversity, normalization of response-associated taxa and metabolites, and profound reorganization of host-microbiota-metabolite interaction networks, with ternary correlations dramatically expanding post-treatment while baseline patterns largely disappeared. These findings highlight network rewiring as a central driver of FMT efficacy across IBD subtypes.

**Fig 2:**
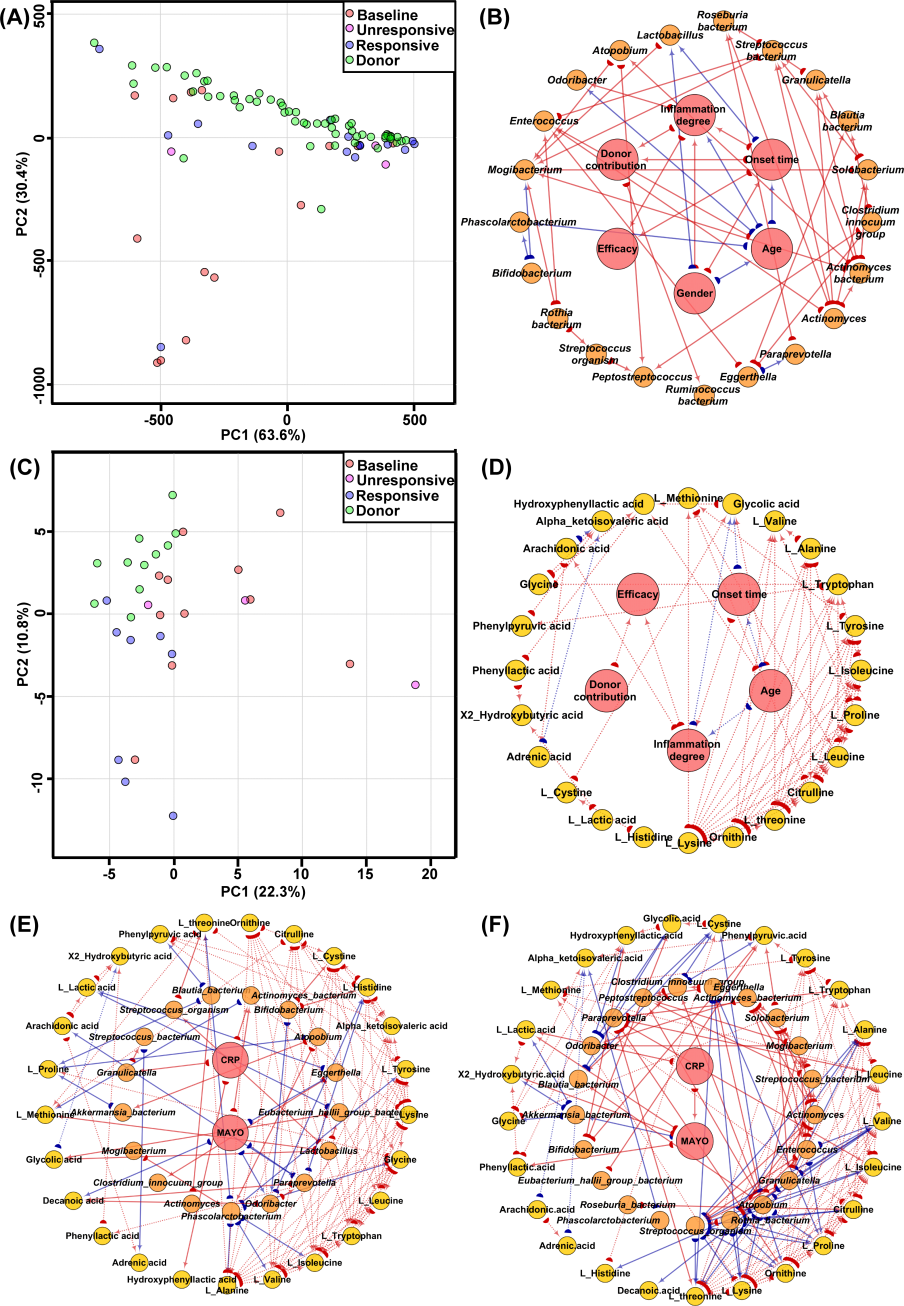
Characteristics of the intestinal microbiome and metabolome in the UC cohort. (**A**) PCA score plot illustrating microbiome profiles among UC baseline subjects, FMT-responsive and unresponsive subjects, and healthy donors, using FMT efficacy-associated microbes as the data set. (**B**) Spearman correlation network showing relationships among FMT efficacy-associated microbes and baseline clinical indicators of the subjects. (**C**) PCA score plot representing metabolomic profiles among UC baseline subjects, FMT-responsive and unresponsive subjects, and healthy donors. (**D**) Spearman correlation network depicting associations among FMT efficacy-associated metabolites and baseline clinical indicators. (**E**) Spearman correlation network of inflammatory indicators, FMT efficacy-related microbes, and metabolites in UC baseline subjects. (**F**) Spearman correlation network of inflammatory indicators, FMT efficacy-related microbes, and metabolites in UC subjects post-FMT. The red arrow suggests a positive correlation, the blue arrow indicates a negative correlation, and the arrow’s direction shows a correlation between the source and target.

Of the 15 UC patients receiving FMT monotherapy, 12 achieved clinical response (partial Mayo score reduction ≥2 points and rectal bleeding subscore decrease ≥1 point) at week 4, while 3 had no response. By week 14, all 12 responders maintained clinical remission (partial Mayo score <3), with 8 achieving endoscopic remission (Mayo endoscopic subscore ≤1) and 4 failing to reach endoscopic remission. UC patients were categorized into baseline, FMT-responsive, and FMT-unresponsive groups based on week 4 clinical response. FMT increased α-diversity indices (ACE, Chao1, OTUs) in most UC patients, particularly non-responders, with no significant baseline-donor differences (Fig. S1). Using screening criteria for FMT response-associated microbes (differential abundance in baseline UC or donor samples, shift to donor levels in responders, persistence at baseline levels in non-responders), 14 genera were identified: 5 Actinobacteriota (*Actinomyces*, *Actinomyces_bacterium*, *Rothia_bacterium*, *Atopobium*, *Eggerthella*) and 9 Firmicutes (*Solobacterium*, *Granulicatella*, *Enterococcus*, *Streptococcus_bacterium*, *Streptococcus_organism*, *Blautia_bacterium*, *Eubacterium_hallii_group_bacterium*, *Mogibacterium*, *Peptostreptococcus*) (Fig. S2 to S5). PCA using these genera distinguished baseline, FMT-treated, and donor profiles (Fig. 2A).

Previously reported UC-associated bacteria (*Bifidobacterium*, *Odoribacter*, *Paraprevotella*, *Clostridium_innocuum_group*, *Lactobacillus*, *Roseburia_bacterium*, *Ruminococcus_bacterium*, *Phascolarctobacterium*, *Akkermansia_bacterium*) showed *Bifidobacterium*, *Odoribacter*, and *Paraprevotella* enrichment at baseline, and *Roseburia_bacterium* and *Ruminococcus_bacterium* depletion (*P* < 0.05, Fig. S6), but none correlated with FMT efficacy (*P* > 0.05).

Spearman correlation networks linked baseline characteristics (age, gender, disease duration, inflammation degree) and FMT response-associated microbes (Fig. 2B). FMT efficacy correlated with disease duration (q = 0.51) and inflammation degree (q = 0.65), but not with differentially enriched microbes or donor contribution. Notably, two non-responders received FMT from *Prevotella*-enterotype donors (6 samples), while others received *Bacteroides-*enterotype FMT (51 samples), with *Prevotella* donors showing higher Simpson indices (Fig. S7). Volcano plot analysis identified 15 donor-associated genera, with *Actinomyces_bacterium* enriched in non-responders and *Prevotella* donors.

PCA of the metabolomic data clearly separated baseline profiles of UC patients from those of donors, with non-responders retaining characteristics similar to their own baseline (Fig. 2C). We identified 41 metabolites associated with response to FMT (Fig. S8 to S10). FMT induced a pronounced shift in amino acid profiles toward donor-like states, whereas bile acid profiles showed more limited correction (Fig. S10 and S11). Pathway enrichment analysis (MetaboAnalyst, HMDB database) confirmed the association of these metabolic changes with IBD-relevant pathways (*P* < 0.05; Fig. S10 and S11).

To identify metabolites with statistically significant differential abundance, we performed hypothesis testing and applied the Benjamini-Hochberg procedure to control the false discovery rate. This yielded 25 significant metabolites (adjusted *P* < 0.05), which were subjected to downstream network analysis. These Spearman correlation networks revealed associations linking FMT efficacy to donor-like metabolomic features and reduced inflammation (Fig. 2D), with disease inflammation emerging as key predictors.

Ternary Spearman correlation networks integrating inflammatory markers (fecal calprotectin, CRP, and Mayo score), microbial taxa, and metabolites revealed 115 correlation pairs at baseline and 155 pairs post-FMT. After excluding metabolite-metabolite correlations, 43 pairs remained at baseline and 95 post-FMT (Fig. 2E and F; red: positive correlations; blue: negative correlations). Notably, only 7% (3 of 43) of the baseline non-metabolite correlations persisted after FMT (Fig. S13), indicating that FMT extensively remodels host-microbe-metabolite interactions.

Among the 15 biologic-naïve patients with CD who received FMT monotherapy, 9 achieved clinical response at week 4 (defined as a ≥100-point decrease in CDAI), 2 showed no response, and 4 were lost to follow-up. By week 14, all nine responders had sustained clinical remission (CDAI < 150), of whom eight also achieved endoscopic remission (SES-CD ≤ 2) and one did not.

**Fig 3:**
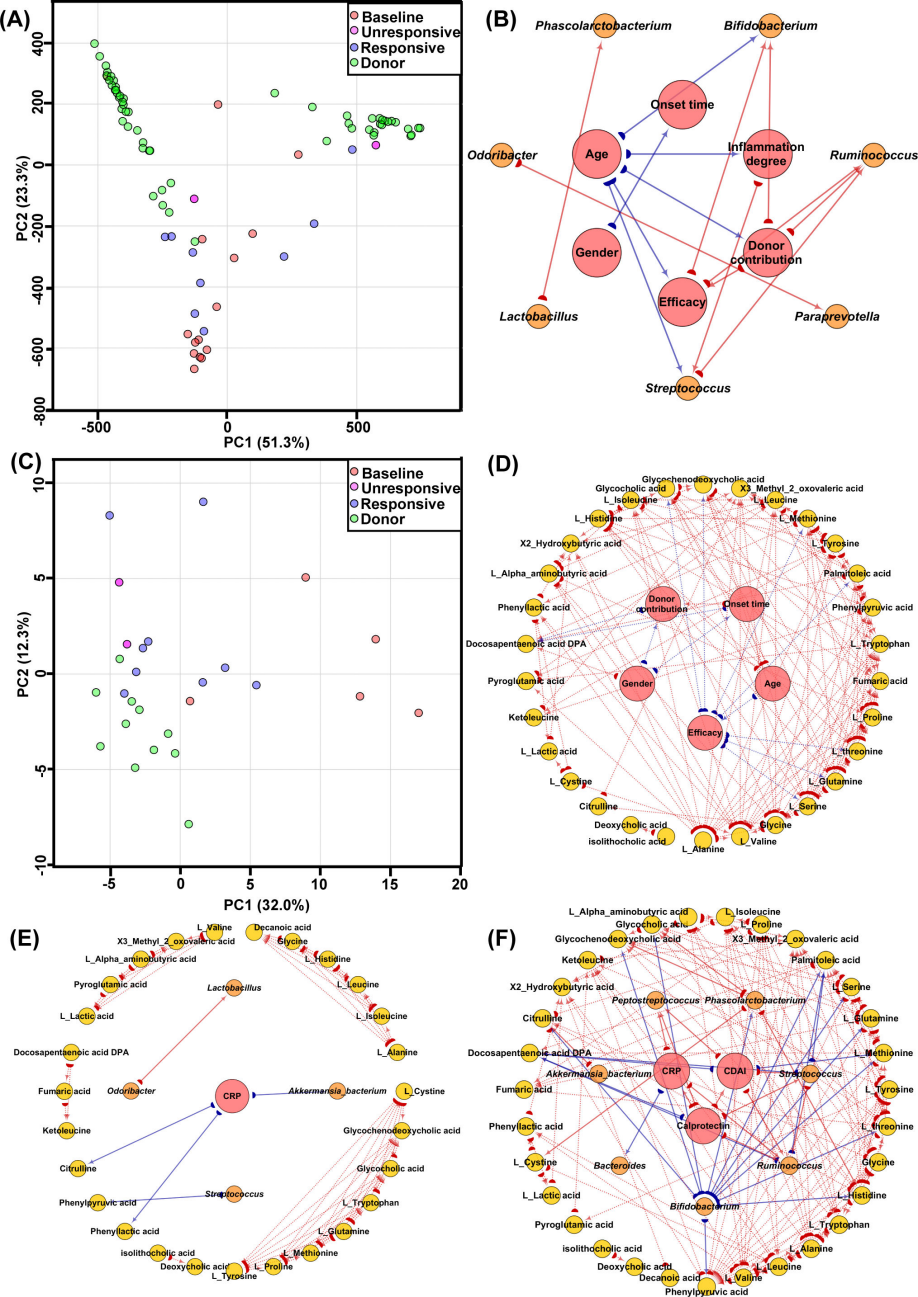
Intestinal microbiome and metabolome characteristics of the CD cohort. (**A**) PCA score plot of microbiome data among CD baseline subjects, FMT-responsive and FMT-unresponsive subjects, and healthy donors, based on FMT-efficacy-associated microbes. (**B**) Spearman correlation network illustrating associations between FMT-efficacy-associated microbes and CD subjects’ baseline indicators. (**C**) PCA score plot of metabolomics data among CD baseline subjects, FMT-responsive and FMT-unresponsive subjects, and healthy donors. (**D**) Spearman correlation network showing relationships between FMT-efficacy-associated metabolites and CD subjects’ baseline indicators. (**E**) Spearman correlation network of inflammatory indicators, FMT-efficacy-associated microbes, and metabolites in CD baseline subjects. (**F**) Spearman correlation network of inflammatory indicators, FMT-efficacy-associated microbes, and metabolites in CD subjects after FMT. The red arrow suggests a positive correlation, the blue arrow indicates a negative correlation, and the arrow’s direction shows a correlation between the source and target.

We identified 17 microbial taxa associated with FMT response, including *Bifidobacterium*, *Streptococcus*, and *Peptostreptococcus*, which were enriched at baseline and normalized to donor-like levels in responders (Fig. S14 to 18). PCA of the microbiomic data clearly separated baseline patient profiles, post-FMT profiles, and donor profiles (Fig. 3A). Additional CD-associated taxa (*Bacteroides*, *Odoribacter*, *Paraprevotella*, *Lactobacillus*, *Roseburia*, *Ruminococcus*, *Phascolarctobacterium*, and an *Akkermansia* bacterium) showed baseline enrichment of *Lactobacillus* alongside depletion of the others (Fig. S19). Compared with donors, baseline CD samples exhibited significantly lower α-diversity (ACE, Chao1, and observed OTUs) and higher Simpson indices (*P* < 0.05); in responders, these diversity metrics shifted toward donor levels after FMT (*P* > 0.05; Fig. S15). Spearman correlation analysis linked FMT efficacy to younger patient age (ρ = −0.59), donor selection (ρ = 1.00), and higher baseline abundance of *Bifidobacterium* (ρ = 0.58) and *Ruminococcus* (ρ = 0.77) (Fig. 3B).

Metabolomic PCA distinctly separated baseline CD profiles from those of responders, non-responders, and donors (Fig. 3C). We identified 50 metabolites associated with FMT response, characterized by elevated amino acids and depleted bile acids at baseline (Fig. S20 to S22). Notably, CD patients displayed dysregulation of primary bile acids (glycocholic and glycochenodeoxycholic acids) and the secondary bile acid deoxycholic acid. Pathway enrichment analysis confirmed the relevance of these metabolites to inflammatory diseases (*P* < 0.05; Fig. S22 and S23). Independent hypothesis testing with false discovery rate correction (Benjamini-Hochberg procedure) identified 31 differentially abundant metabolites for downstream analyses. FMT efficacy was negatively correlated with levels of L-glutamine, L-serine, L-threonine, L-methionine, glycocholic acid, glycochenodeoxycholic acid, and palmitoleic acid (Fig. 3D).

Ternary Spearman correlation networks integrating inflammatory markers, microbial taxa, and metabolites revealed 62 correlation pairs at baseline and 145 pairs post-FMT; after excluding metabolite-metabolite correlations, these numbers decreased to 5 and 35 pairs, respectively (Fig. 3E and F; red: positive correlations; blue: negative correlations). None of the baseline non-metabolite correlation patterns persisted after FMT (Fig. S24 and SS25).

Collectively, FMT monotherapy in both UC and CD responders drove comparable remodeling of the gut ecosystem: restoration of microbial diversity, normalization of key taxa and metabolites, and a marked expansion of host-microbiota-metabolite interactions with near-complete loss of baseline correlation patterns. Despite subtype-specific predictors (namely, disease duration and inflammatory status in UC, and younger age with higher baseline abundance of *Bifidobacterium* and *Ruminococcus* in CD), the shared signature of extensive network reorganization suggests this rewiring is fundamental to FMT’s therapeutic effect and provides a benchmark for evaluating combination strategies in refractory disease.

### Combined IFX-FMT therapy achieves superior remission in monotherapy-resistant patients via additive network optimization

**Fig 4:**
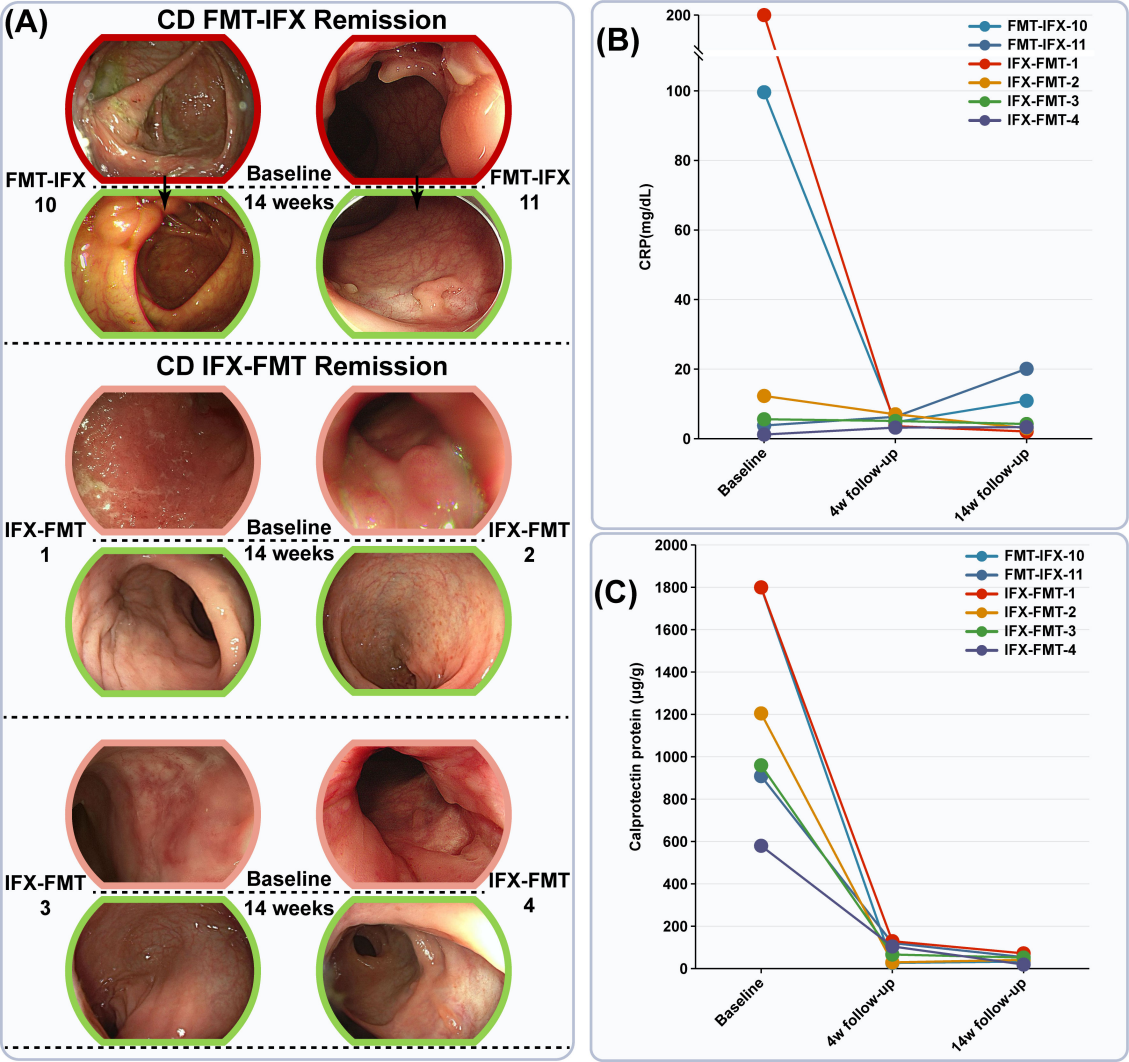
Combined FMT and IFX therapy induces remission in CD patients unresponsive to monotherapy. (**A**) Colonoscopy images from two CD patients unresponsive to FMT and four CD patients unresponsive to IFX, assessed before treatment and after 14 weeks of combined FMT-IFX therapy. (**B**) C-reactive protein levels were measured at baseline, 4-week follow-up, and 14-week follow-up in the six CD patients. (**C**) Fecal calprotectin levels were assessed at baseline, 4-week follow-up, and 14-week follow-up in the six CD patients. CRP, C-reactive protein.

**Fig 5:**
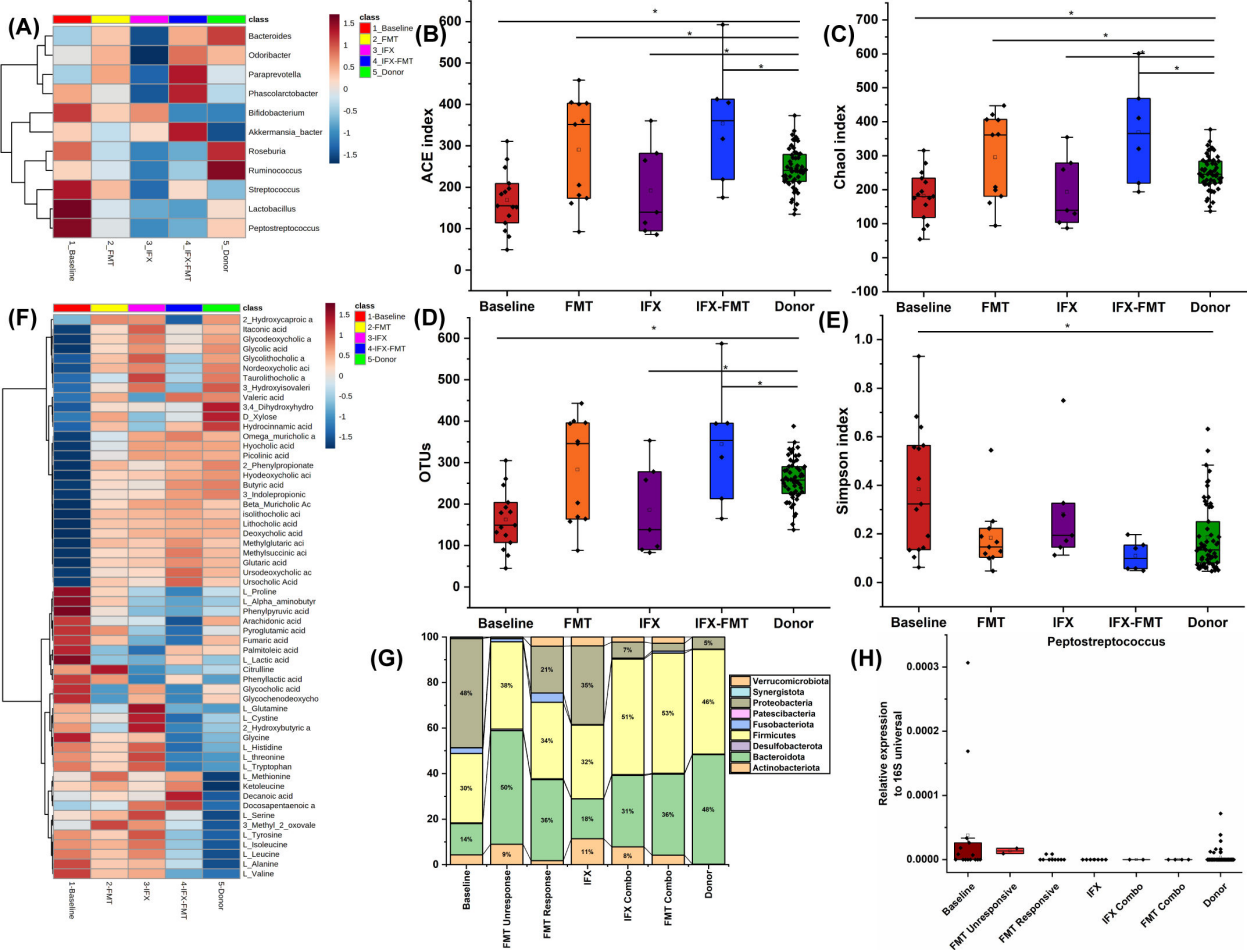
Changes in intestinal microbiome and metabolome in CD patients treated with FMT, IFX, and IFX-FMT combined therapy. (**A**) Heatmap displaying the relative abundance of CD FMT efficacy-associated microbes in CD baseline subjects, donors, and CD patients receiving FMT, IFX, or IFX-FMT combined therapy. (**B–E**) Comparison of α-diversity indices among the five groups. (**F**) Differences in metabolites associated with FMT efficacy among the five groups, highlighting metabolomic shifts. (**G**) Differences in microbiota composition at the phylum level, showcasing taxonomic changes. (**H**) Changes in *Peptostreptococcus* abundance, a key microbe linked to FMT response, across the groups.

Nine CD patients (seven with primary IFX non-response, two FMT non-responders) received IFX-FMT combination therapy. Specifically, for the seven IFX non-responders, a single FMT was administered while continuing IFX maintenance dosing; for the two FMT non-responders, a second FMT was given alongside IFX re-induction. At week 4, six patients achieved clinical response (CDAI decrease ≥ 100 points) (Fig. 4A), while three were lost to follow-up. By week 14, all six patients maintained clinical response and achieved both clinical remission (CDAI < 150) and endoscopic remission (SES-CD ≤ 2) (Table 1, Fig. 4B and C). IFX non-responders showed depleted *Odoribacter* and *Paraprevotella* but enriched *Bifidobacterium* and *Akkermansia_bacterium* (Fig. 5A). They had lower α-diversity (OTUs, ACE, Chao1) and higher Simpson indices, with elevated Proteobacteria and reduced Firmicutes/Bacteroidota (Fig. 5B through E and G). Metabolomics revealed higher bile acids and persistent amino acid elevations (L-histidine, L-tryptophan, L-threonine, L-tyrosine) in IFX non-responders (Fig. 5F).

IFX-FMT restored α-diversity to donor levels (Fig. 5B through E). Sixty-eight microbes were associated with combination therapy, with PCA and Venn analyses showing integrated IFX and FMT signatures (Fig. S26 and 27). *Bacteroidota*, *Odoribacter*, and *Paraprevotella* increased to donor levels in responders (Fig. 5A). Metabolomic profiles shifted toward donors, with most bile acids (except glycocholic and glycochenodeoxycholic) and amino acids (L-histidine, L-valine, L-isoleucine, L-leucine) normalized (Fig. 5F). In FMT non-responders, Actinobacteriota increased, Proteobacteria decreased (<1%), and *Peptostreptococcus* reduced to donor levels (Fig. 5H; Fig. S29 to S31). Amino acids normalized post-combination therapy (Fig. S32 and S23). In summary, IFX-FMT combination therapy demonstrated combined microbial and metabolomic effects, with five of nine CD patients who failed initial IFX or FMT therapy achieving clinical and endoscopic remission at 14 weeks.

## DISCUSSION

Gut dysbiosis and disrupted host-microbiota-metabolite interactions play critical roles in IBD pathogenesis, yet the mechanisms underlying therapeutic modulation of these networks remain incompletely understood (18–24). This study investigated FMT monotherapy in UC and biologic-naïve CD patients refractory to conventional therapies, and its combination with IFX in CD patients unresponsive to either modality alone, using a fixed individual donor to ensure omics consistency.

FMT monotherapy induced substantial clinical and endoscopic remission, with consistent microbial and metabolomic restoration across IBD subtypes. In responders, response-associated taxa and metabolites shifted toward donor-like levels, accompanied by increased α-diversity and enrichment of pathways relevant to IBD. Most strikingly, ternary Spearman correlation networks revealed profound reorganization in both subtypes: dramatic expansion of host-microbiota-metabolite interactions post-FMT, with near-complete loss of baseline patterns. This extensive network rewiring establishes reorganization of gut ecosystem interactions as a central mechanism driving FMT efficacy, though subtype-specific clinical predictors were observed. In contrast, IFX monotherapy in primary non-responders only partially corrected dysbiosis, with persistent abnormalities in microbial composition, metabolomic profiles, and interaction networks.

The potential additive effects between FMT and biologics may stem from complementary gut microbiota-immune interactions (29). Biologics (e.g., infliximab) block pro-inflammatory pathways (TNF-α), while FMT restores microbial balance and generates anti-inflammatory metabolites. For example, L-ornithine from *Faecalibacterium prausnitzii* suppresses the EGR1–IL12RB1 axis and Th17 activity, potentially amplifying biologic effects. The pilot trial (NCT06455267) showed L-ornithine plus ustekinumab improved endoscopic remission in CD (30). However, long-term efficacy, safety, and mechanisms in refractory IBD require further validation through larger trials.

International studies conducted outside China—primarily in Europe, North America, and Australia—provide valuable context for interpreting our findings, particularly regarding FMT as monotherapy or adjunctive therapy alongside conventional (e.g., 5-ASA, corticosteroids, immunomodulators) or advanced treatments (biologics and small molecules). Most evidence derives from observational cohorts, small series, and subgroup analyses of randomized trials rather than direct controlled comparisons. For example, Moayyedi et al. reported that FMT by enema induced clinical remission in 24% of active UC patients versus 5% with placebo, with many patients on stable concomitant 5-ASA or corticosteroids showing no diminution of FMT benefit (31). Similarly, Sokol et al. demonstrated that FMT (versus sham) helped maintain steroid-free remission and prevented postoperative recurrence in Crohn’s disease, suggesting additive effects when combined with prior steroid induction (17). Kedia et al. showed that multidonor FMT combined with an anti-inflammatory diet (AID) induced remission in 46.2% of mild-to-moderate UC patients versus 23.3% with AID alone, with 1-year maintenance rates of 61.5% versus 33.3%, underscoring sustained benefits from this adjunctive strategy (32). Pooled North American data encompassing >100 IBD cases have reported overall remission rates of 45%–60% with FMT, frequently in patients on background conventional or biologic therapy, with no significant adverse impact of concomitant biologics, steroids, or antibiotics on outcomes (*P* = 0.28–0.47) (33). These findings align with the clinical response rates observed in our cohort (~60%) and indicate that concomitant therapies generally do not impair—and may complement—FMT efficacy across diverse patient populations and treatment norms. Nonetheless, international studies have largely emphasized clinical and endoscopic endpoints, with limited multi-omics interrogation of microbiome and metabolome dynamics under combination regimens. Our work addresses this gap by providing detailed pre- and post-treatment profiling in patients receiving FMT alongside infliximab, identifying specific microbial and metabolic signatures associated with response in refractory Crohn’s disease.

This study has several limitations inherent to its exploratory pilot design. As a single-center investigation with a modest sample size (37 IBD patients), statistical power and generalizability are limited. Furthermore, the lack of a parallel IFX monotherapy control arm precludes definitive attribution of the observed benefits in the combination group to additive interaction rather than additive or independent effects. The restricted number of clinical events precluded reliable multivariable adjustment for potential confounders (e.g., age, disease duration, baseline inflammation severity). To overcome these limitations and provide higher-level evidence, we have initiated a multicenter randomized controlled trial (ClinicalTrials.gov identifier NCT07149441) involving 20 tertiary hospitals in China. This ongoing study aims to enroll approximately 290 IBD patients who have not achieved clinical remission during maintenance therapy with advanced biologics. It directly compares FMT combined with advanced therapy versus advanced therapy alone over 52 weeks, with the primary endpoint of remission rate at week 52 and secondary endpoints, including long-term adverse event rates and pre/post-treatment microbiome changes.

In conclusion, FMT modulates the IBD microbiome and metabolome by extensively rewiring host-microbiota-metabolite networks, inducing remission in patients refractory to conventional therapies. IFX-FMT combination shows preliminary evidence of additive benefit in monotherapy-resistant refractory CD. The week 14 clinical and endoscopic remission data, together with planned longer-term follow-up, strengthen these insights and support larger trials to advance microbiome-directed strategies in IBD.

## Data Availability

All data generated or analyzed during this study are included in this published article and its supplemental material. Protocols and raw data are available at the MetaboLights database (https://www.ebi.ac.uk/metabolights/editor/MTBLS3909/descriptors) and the National Center for Biotechnology Information server under study accession numbers PRJNA850886 and PRJNA940621. As absolute quantification technology was used in the metabolomics analysis of this study; the raw data can be accessed via the dataset files under MTBLS3909. For any technical inquiries, please contact the corresponding author.

## SUPPLEMENTAL MATERIAL

Supplemental material msystems.00774-25-s0001.pdf Supplemental text, figures, and tables.

ASM does not own the copyrights to Supplemental Material that may be linked to, or accessed through, an article. The authors have granted ASM a non-exclusive, world-wide license to publish the Supplemental Material files. Please contact the corresponding author directly for reuse.
