# Supplementary material for: Additive effects of fecal microbiota transplantation and infliximab on gut microbiome and metabolome in refractory inflammatory bowel disease patients
Source: mSystems. 2026 Mar 23;11(4):e00774-25. doi: 10.1128/msystems.00774-25 (PMC13098230; doi:10.1128/msystems.00774-25)
Supplement: Supplemental material — Supplemental text, figures, and tables. [file msystems.00774-25-s0001.pdf]

Supplementary Information：

Additive Effects of Fecal Microbiota Transplantation and Infliximab on Gut Microbiome and Metabolome in Refractory Inflammatory Bowel Disease Patients

Xinjun Wang[1,2,3,4,5,6,*], Weijun Wu[8], Bo Yang[1,3,4,5], Yunkun Liu[1,3,4,5], Yue Xu[1,3,4,5], Le Wang[1,3,4,5], Xiaoqiong Lv[1,3,4,5], Junhui Gao[6], Man Lu[6], Anqi Yu[6], Ning Li[1,3,4,5], Qiyi Chen[1,3,4,5], Liesheng Lu[7,*], Di Zhao[1,3,4,5,*]

1 Department of Functional Intestinal Diseases, General Surgery of Shanghai Tenth People's Hospital, Tongji University School of Medicine, Shanghai, China.

2 Affiliated Suzhou Hospital of Nanjing Medical University, Suzhou Municipal Hospital, Gusu School, Nanjing Medical University, Suzhou, China.

3 Shanghai Gastrointestinal Microecology Research Center, Shanghai, China.

4 Shanghai Institution of Gut Microbiota Research and Engineering Development, Shanghai, China.

5 Clinical Research Center for Digestive Diseases, Tongji University School of Medicine, Shanghai, China

6 Shanghai Zhangjiang Institute of Medical Innovation, Shanghai, China.

7 Department of Gastrointestinal Surgery, General Surgery of Shanghai Tenth People's Hospital, Tongji University School of Medicine, Shanghai, China.

8 Department of Pathology, Shanghai Tenth People’s Hospital, Shanghai, China

* Corresponding to Xinjun Wang (xjwang16@fudan.edu.cn), Liesheng Lu (luliesheng1980@163.com), Di Zhao (dizhaomd@vip.163.com); Xinjun Wang is the lead contact for this project.

Contents

S1. Eeperimental section............. 2

S2. Trajectory of the microbiome of UC patients receiving FMT............. 2

S3. Host-microbe co-metabolites disordered in UC patients............. 9

S4. Inflammation-driven microbe-metabolite network interaction of UC patients............. 13

S5. Trajectory of the microbiome of CD patients receiving FMT............. 15

S6. Host-microbe co-metabolites disordered in CD patients............. 20

S7. Inflammation-driven microbe-metabolite network interaction of CD patients............. 24

S8. Intervention effect on intestinal microbiota diversity by FMT or IFX treatment can be inherited by IFX-FMT combination treatment............. 26

S9. Analysis of failure cases in monotherapy............. 29

S10. Intervention effect on host-microbe co-metabolism by FMT or IFX treatment also can be inher-ited by IFX-FMT combination treatment............. 32

S11. Annexed tables............. 35

S1. EXPERIMENTAL SECTION

Study population. 37 IBD patients were prospectively recruited from Shanghai Tenth People's Hospital from October 2019 to May 2021 (Figure 1A). Among them, 15 were diagnosed as UC, and 22 were diagnosed as CD (Table S1). For CD patients, 15 were received FMT treatment, 7 were received infliximab IFX treatment. All IFX treated CD patients did not present clinical response, and 2 of them eventually took concomitant FMT treatment. 2 FMT-unresponsive CD patients also accept IFX-FMT combination therapy, aiming to obtain a rapid induction into remission. Healthy donors were recruited from Shanghai Tenth People's Hospital (n=16). For IBD patients, the first copy of feces sample was collected after diagnosis, and the second copies were collected at the end of FMT treatment (Figure 1B). Due to the little amount of microbiota in watery stool, all collected stool samples were prioritized for microbiome analysis.

S2. Trajectory of the microbiome of UC patients receiving FMT

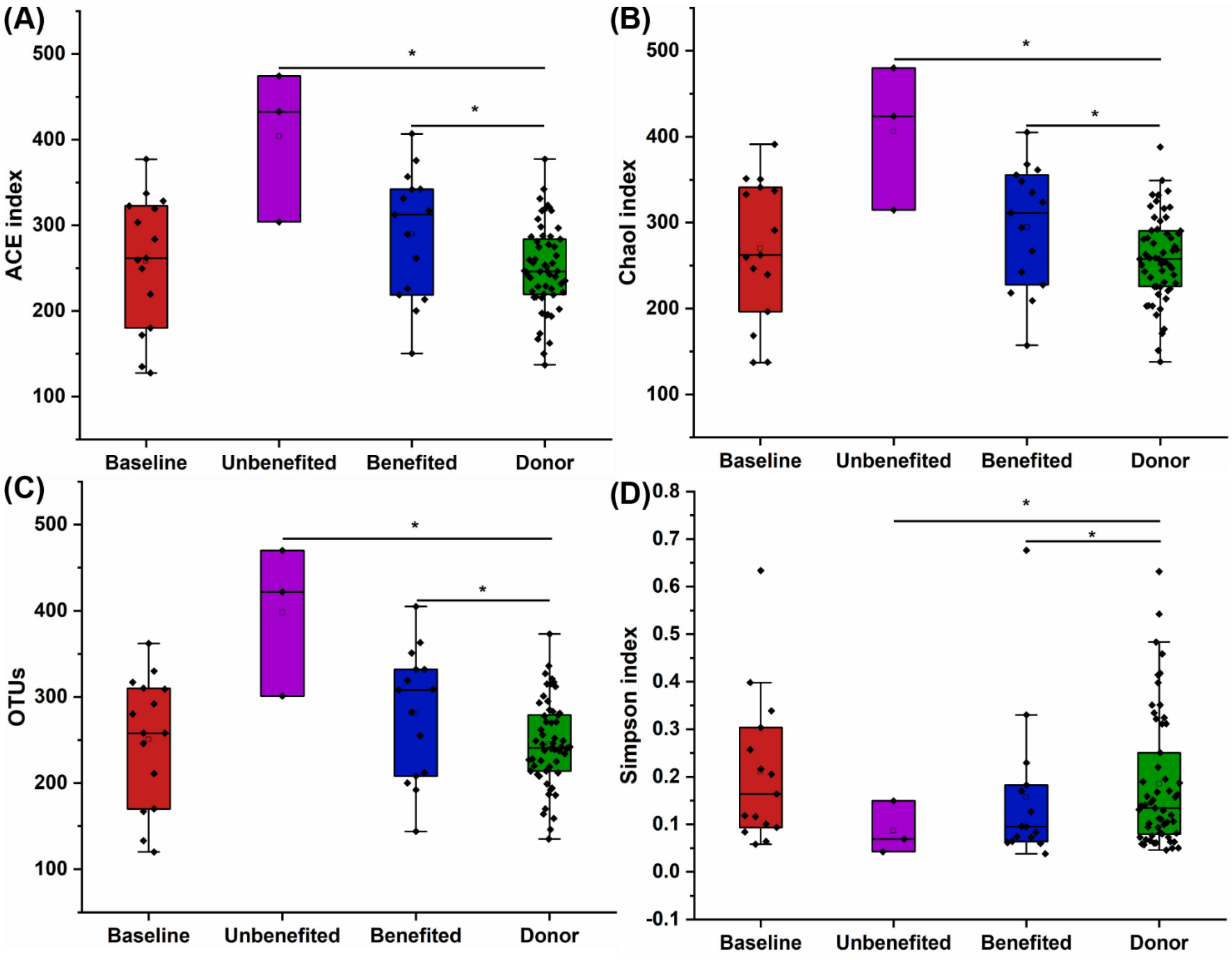

Figure S1. The α-diversity indexes of (A) ACE index, (B) Chao I index, (C) OTUs and (D) Simpson index among UC baseline subjects, FMT responsive and unresponsive subjects, and healthy donors.

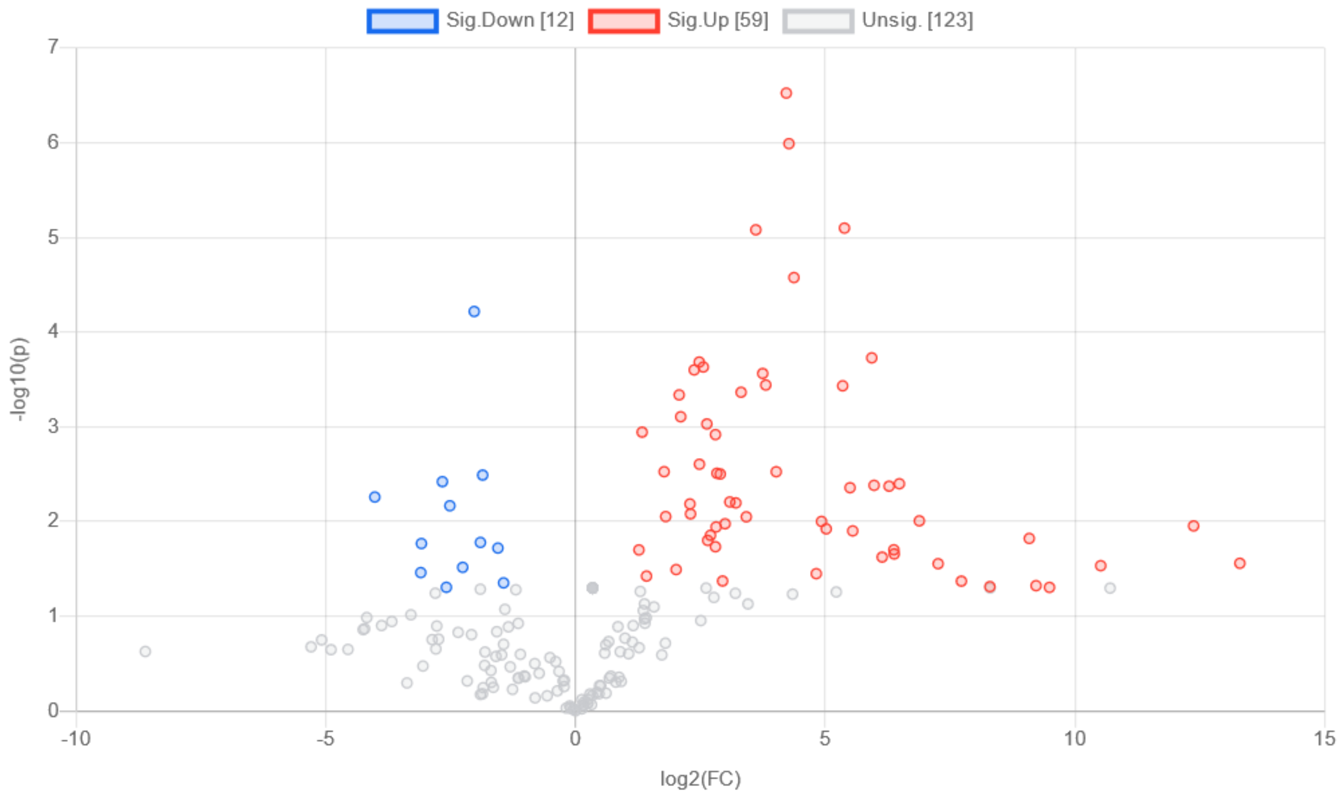

Figure S2. Volcano plot of microbe abundance between donors and UC baseline subjects. Legend: sig. down, downregulation relative to donor subjects; sig. up, upregulation relative to donor subjects; unsig, no significant difference between UC and donor subjects.

To find the trajectory of patients' microbiota changing during FMT treatment, we first used the volcano plot analysis to find the differentially expressed microbes between baseline and donor subjects. As shown in Figure S2 and Table S2, 12 microbes were significantly down-regulated and 59 microbes were up-regulated in UC baseline subjects.

(A)

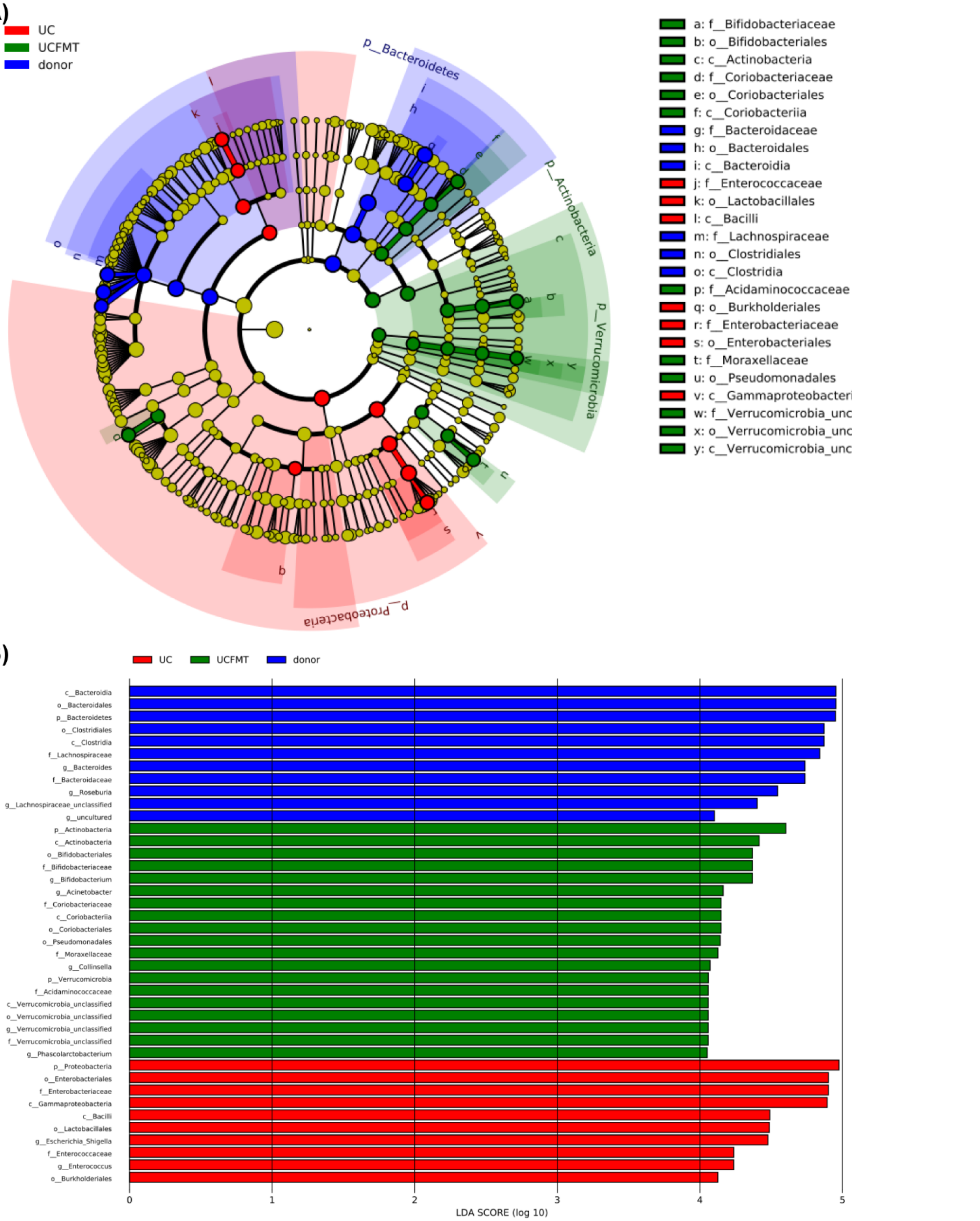

Figure S3. Linear discriminant analysis (LDA) integrated with effect size (LEfSe). (A) Cladogram indicating the phylogenetic distribution of microbiota correlated with UC patients (before and after

FMT treatment) and donors. (B) The microbiota abundance differences among UC patients (before and after FMT treatment) and donors.

The microbiota changes on the predominant taxon between UC and donor subjects was demonstrated by LEfSe analysis with threshold of LDA scores (log10) = 4 (Figure S3). Opportunistic pathogens, including *Enterobacteriaceae*[1] and *Gammaproteobacteria*[1], were significantly enriched in UC baseline group. *Enterobacteriaceae*, belonging to *Proteobacteria* phylum, promises a large family of Gram-negative bacteria, mass propagation under intestinal inflammation induced obligate-anaerobes-decreased environment, and is responsible for substantial infection-related illness and death worldwide.[2] *Gammaproteobacteria*, also belonging to *proteobacteria* phylum, is emerged as a hallmark of acute mucosal infections and enhanced pathology, and has been linked to the etiology of IBD.[3] In contrary, beneficial bacteria of *Roseburia*[4] was enriched in donor group. *Roseburia*, a butyrate-producing microbe, can alleviate colitis symptom by balancing Treg/Th17 proportion and protect intestinal epithelial barrier.[5-7] *Roseburia*-produced butyrate was previously found to be significantly reduced in feces of IBD patients,[8] and its recovery is usually positively correlated with the prognosis.[9, 10] Via FMT treatment, the opportunistic pathogens mentioned above were successfully decreased, and some beneficial bacteria gradually possessed domination, such as *Verrucomicrobiales* and *Bifidobacterium* (Figure S3).

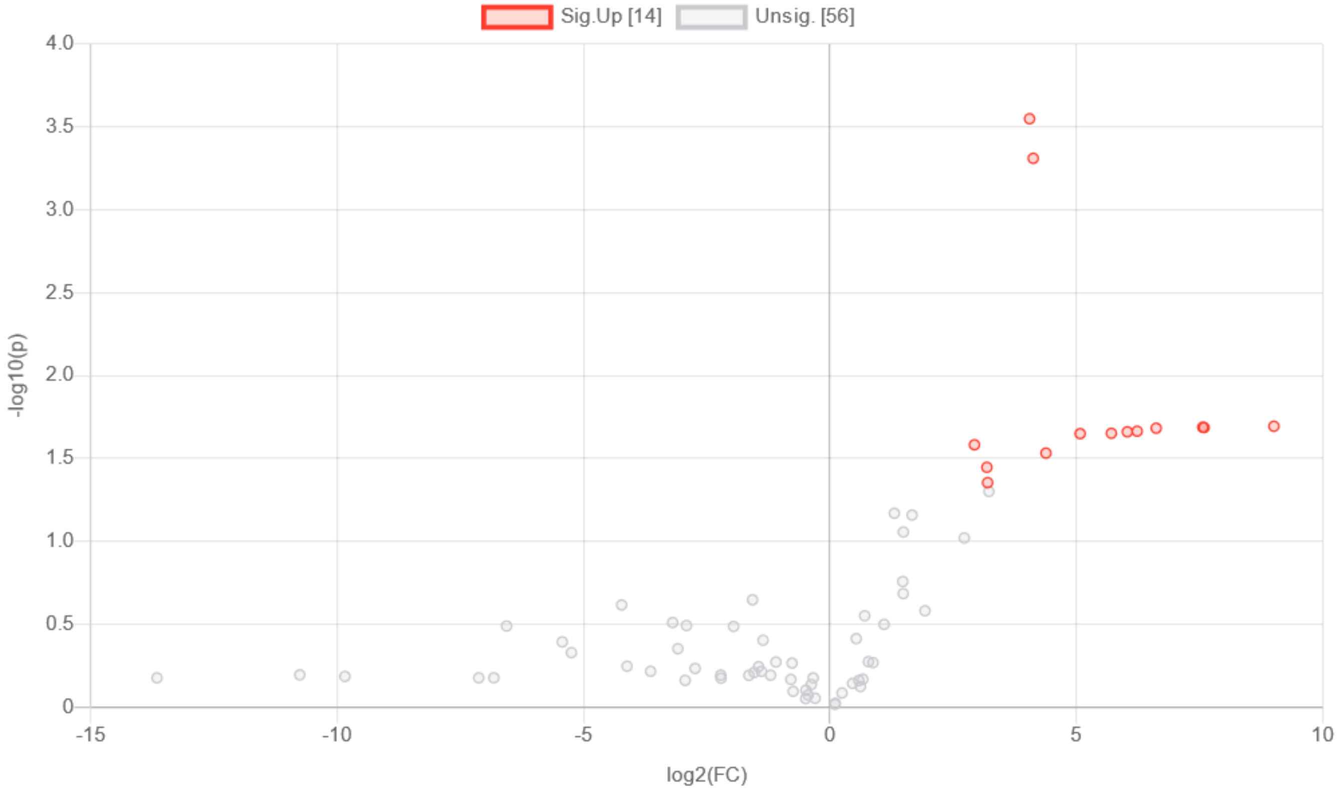

Figure S4. Volcano plot of microbe abundance between FMT responsive and unresponsive UC subjects. Legend: sig. up, upregulated microbe of FMT unresponsive patients relative to FMT responsive patients; unsig, no significant difference between FMT responsive and unresponsive subjects.

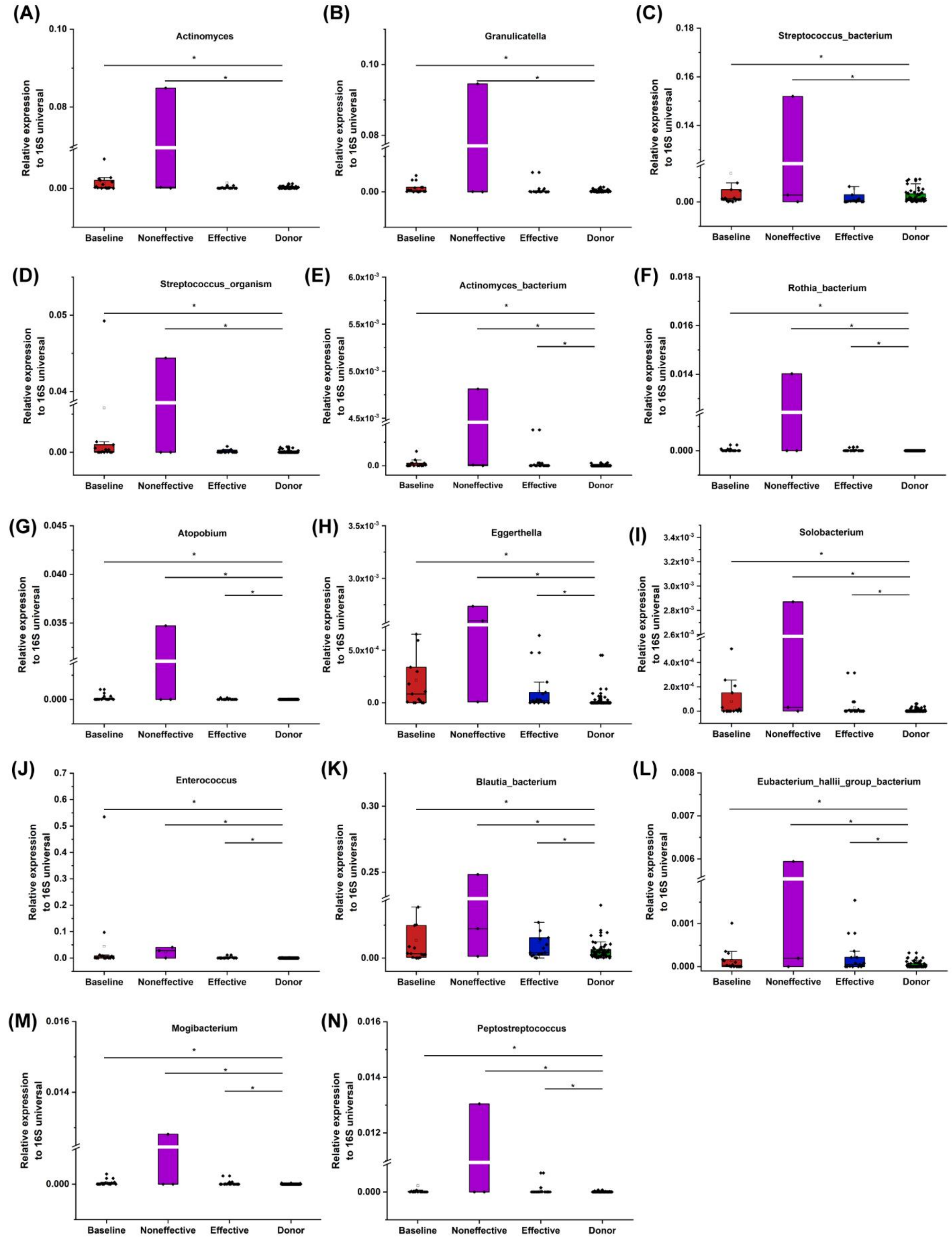

Figure S5. (A-N) The relative abundance of FMT efficacy-associated microbes among UC baseline, FMT responsive/unresponsive patients, and healthy donors.

For further identifying FMT efficacy-associated microbes, we performed volcano plot analysis between FMT responsive and unresponsive patients under the data set of the above 71 UC-associated microbes (Figure S4). Totally 14 microbes, including 5 *Actinobacteriota* (*Actinomyces*, *Actinomyces_bacterium*, *Rothia_bacterium*, *Atopobium*, and *Eggerthella*) and 9 *Firmicutes* (*Solobacterium, Granulicatella*, *Enterococcus*, *Streptococcus_bacterium*, *Streptococcus_organism*, *Blautia_bacterium*, *Eubacterium_hallii_group_bacterium*, *Mogibacterium*, and *Peptostreptococcus*), were identified enrichment in FMT unresponsive patients (Table S3). Through these 14 microbes, the PCA score plot showed a distinct phenotype difference between UC FMT and baseline subjects (Figure 2A). More importantly, part of these 14 microbes (including *Actinomyces*, *Granulicatella*, *Streptococccus_bacterium*, and *Streptococcus_organism*) were enriched in the UC baseline (p-value < 0.05), and decreased or even reached to the donor level after FMT treatment (p-value > 0.05) (Figure S5). On the other hand, traditional UC differentially changed microbes were also examined, including *Bifidobacterium* (belonging to *Actinobacteriota*); *Odoribacter* and *Paraprevotella* (belonging to *Bacteroidota*); *Clostridium_innocuum_group*, *Lactobacillus*, *Roseburia_bacterium*, *Ruminococcus_bacterium*, *Phascolarctobacterium* (belonging to *Firmicutes*); *Akkermansia_bacterium* (belonging to *Verrucomicrobiota*). Among them, *Bifidobacterium*, *Odoribacter* and *Paraprevotella* were identified enrichment in baseline subjects, but *Roseburia_bacterium* and *Ruminococcus_bacterium* were depleted in baseline patients (p-value < 0.05) (Figure S6). Even though, all of these traditional pathogenic microbes did not show sufficient correlation with FMT efficacy in our UC cohort (p-value > 0.05 between FMT responsive and unresponsive groups). However, considering the small number of our cohort and the confirmed relationship of these traditional bacteria to IBD prognosis, we still cautiously regard them as potential FMT efficacy contributors to perform subsequent analysis.

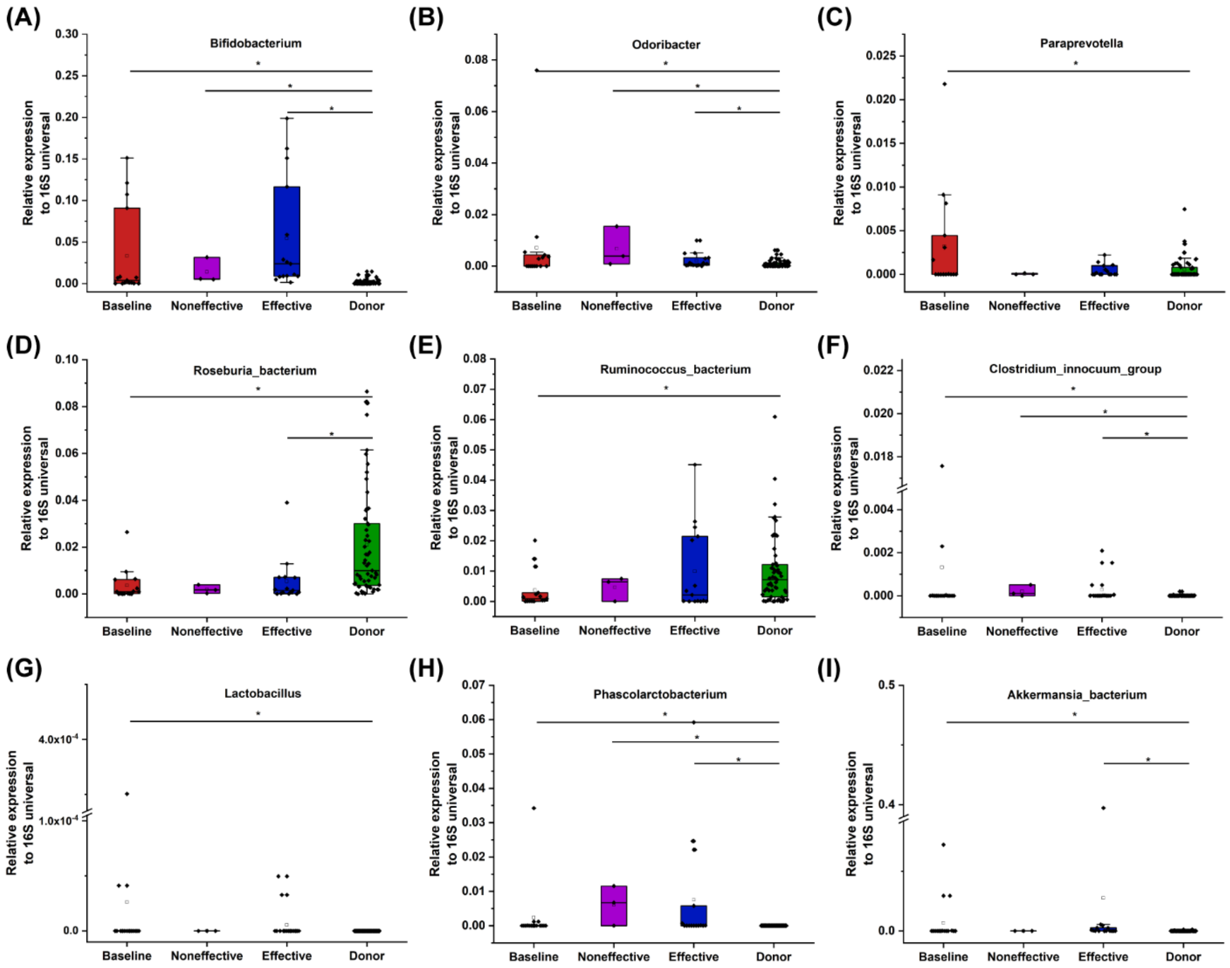

Figure S6. (A-I) The relative abundance of reported UC prognosis-associated microbes among UC baseline, FMT responsive/unresponsive patients, and healthy donors.

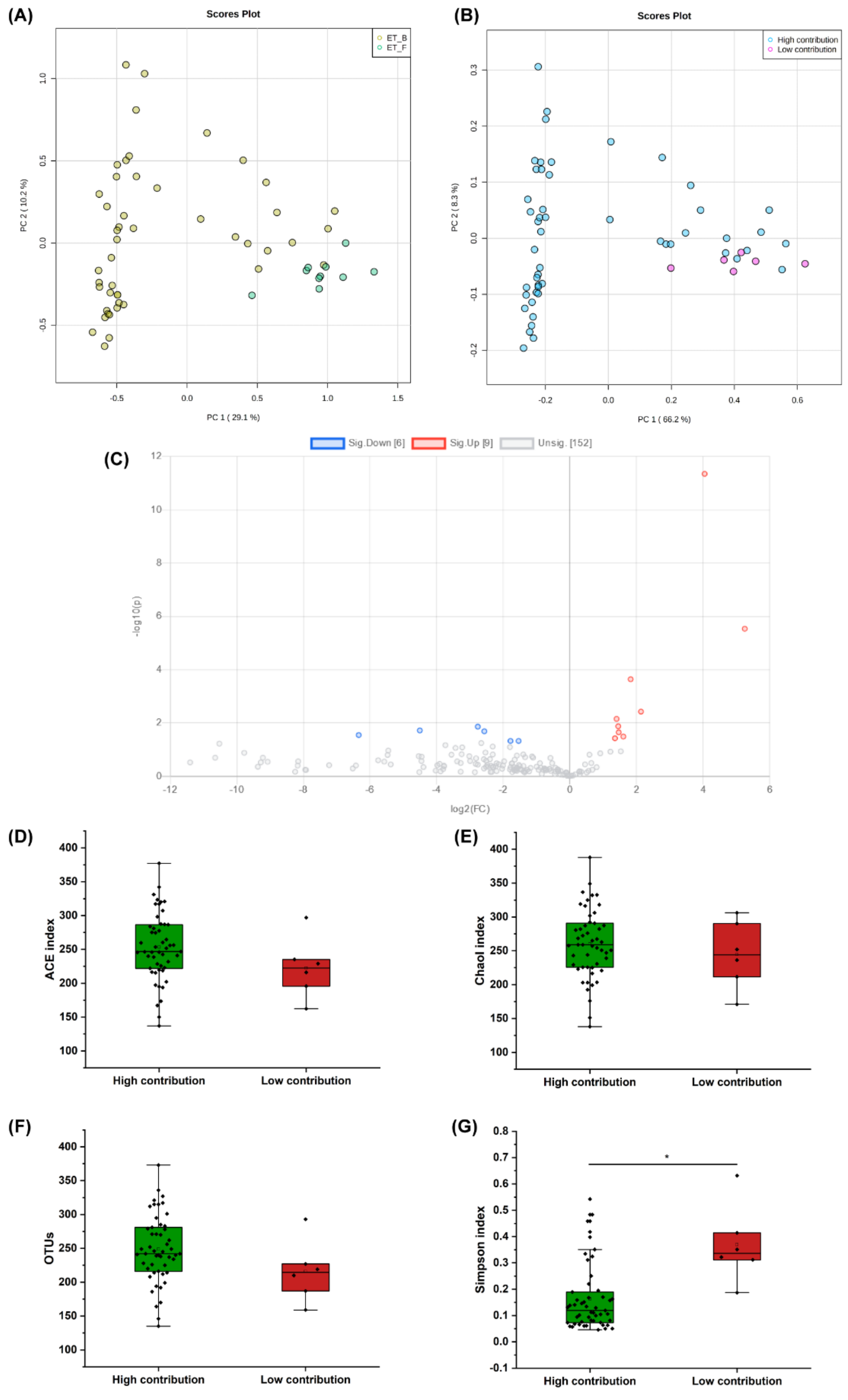

Figure S7. Donors' contribution to FMT efficacy. (A) Enterotype distinguishing among donor subjects. (B) PCA score plot of donors' microbes between high- and low-contributed donors. (C) Volcano plot of microbe abundance between high- and low-contributed donors. (D-G) α diversity indexes difference between high- and low-contributed donors.

S3. Host-microbe co-metabolites disordered in UC patients.

In the PCA score plot, UC baseline metabolome showed a distinct difference relative to donor subjects (Figure 2C). Moreover, FMT unresponsive subjects presented baseline-like characteristics and were separated from the FMT responsive subjects (Figure 2C). Partial least squares discrimination analysis (abbr. PLS-DA) was then used to identify differentially changed metabolites between donors and UC baseline subjects (Figure S8A). The R^2 and Q^2 of PLS-DA model was 82% and 50% respectively, indicating good interpretability and predictability of the constructed classification model.[11] Analysis of PLS-DA variable importance for the projection (VIP) showed 65 UC differentially changed metabolites with VIP values greater than 0.9 (Figure S8B and Table S4). These UC differentially changed metabolites were inclusive of 22 bile acids, 16 amino acids, 11 fatty acids, 9 organic acids, 3 benzenoids, 3 phenylpropanoic acids, and 1 indole. More specifically, amino acid and fatty acid metabolites were abundant, but bile acid metabolites were depleted in UC baseline patients. Organic acid metabolites were not found a consistent trend among this case-control study (Figure S8C). The dysbiosis of these metabolite classes were consistent with previous findings.[12]

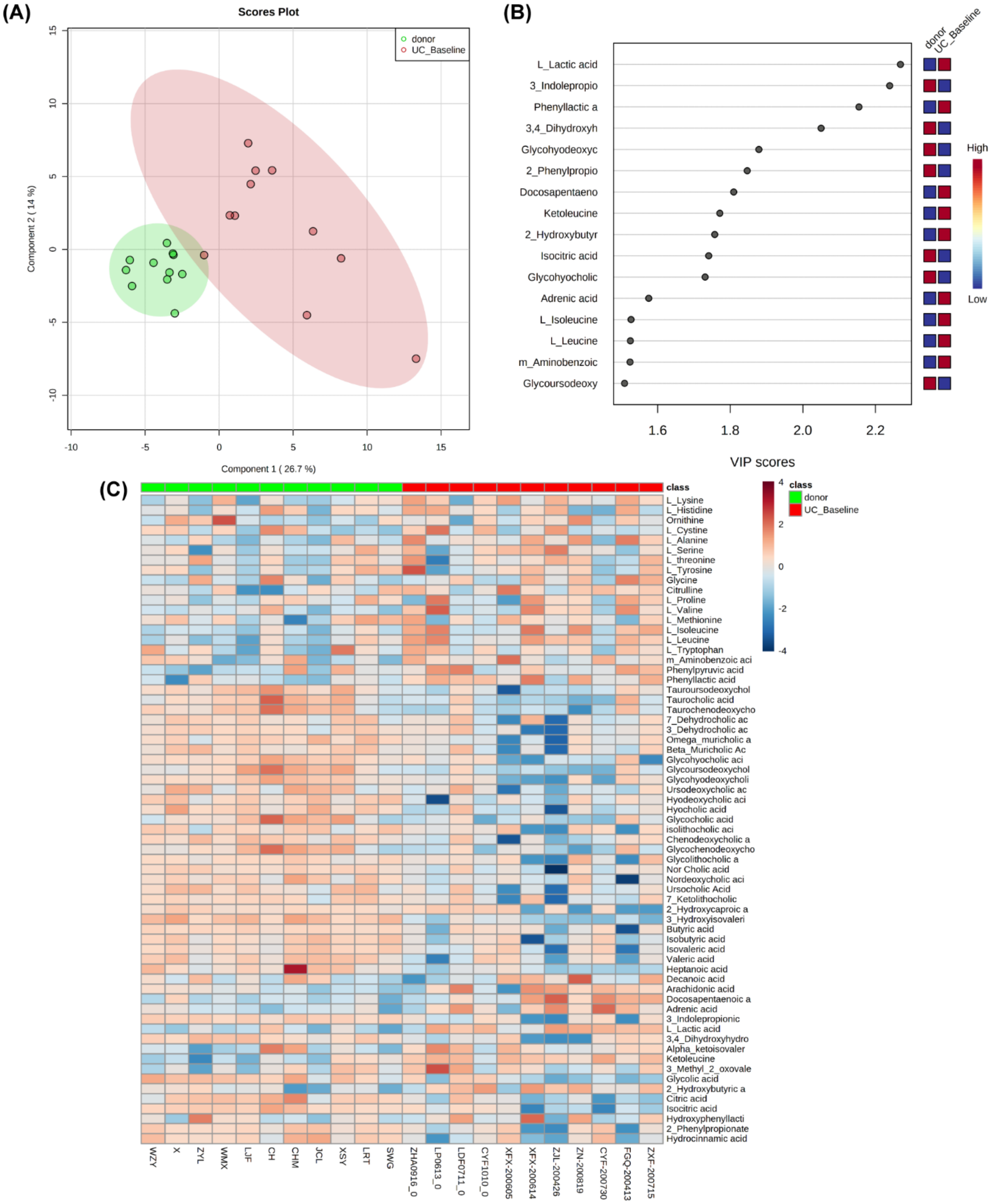

Figure S8. Metabolome difference between UC baseline subjects and donors. (A) PLS-DA score plot of metabolome between UC baseline subjects and donors. (B) Top 16 of key metabolites with VIP value more than 0.9. (C) Heatmap of 65 UC differentially expressed metabolites.

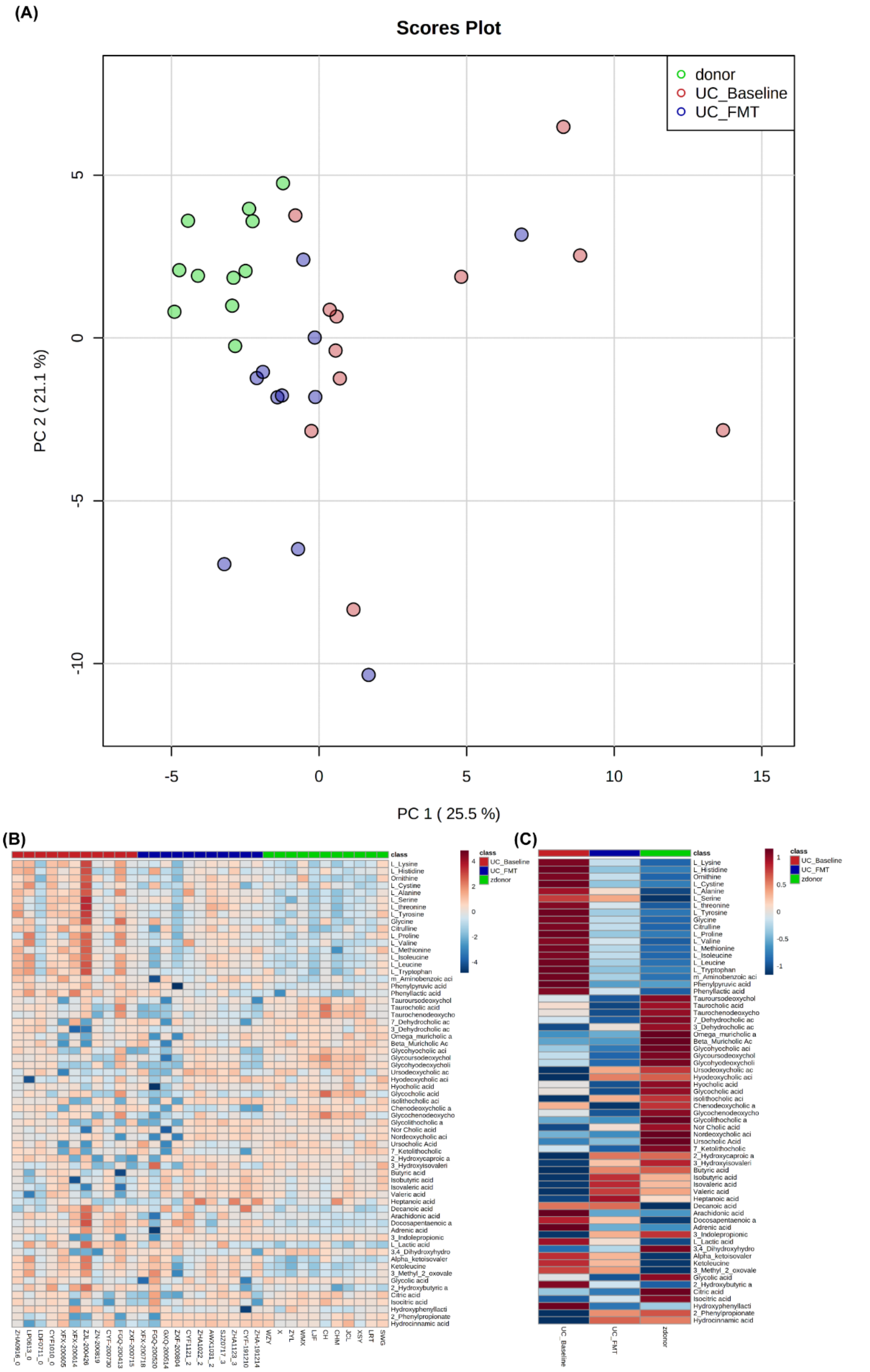

Figure S9. Metabolome difference among UC baseline, UC FMT, and donor subjects. (A) PCA score plot of UC differentially expressed metabolites among UC baseline, UC FMT, and donor subjects. Corresponding (B) heatmap and (C) mean-value heatmap among these three groups.

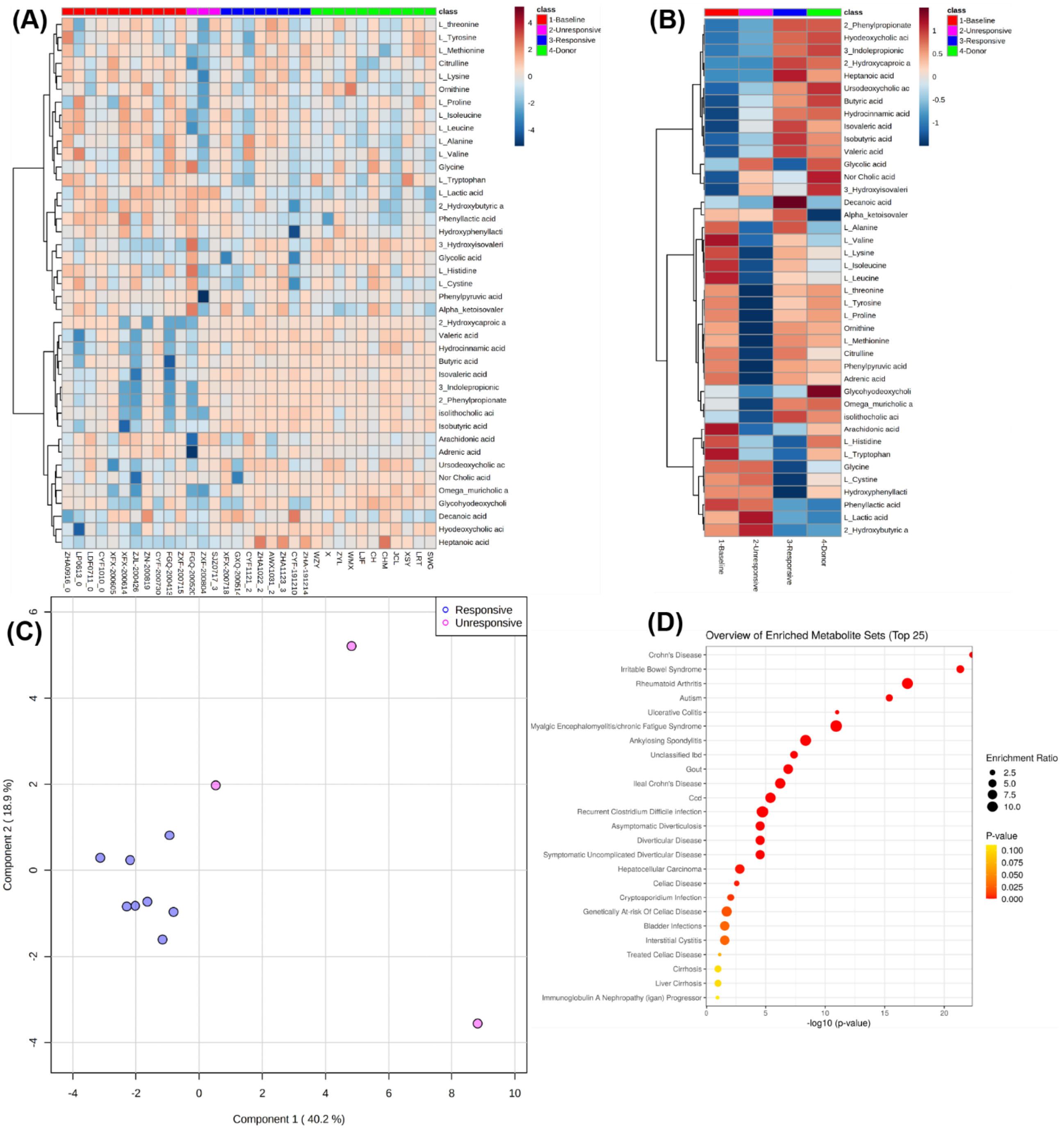

Figure S10. (A) Heatmap of the expression of FMT response associated metabolites that differentiate among UC baseline subjects, FMT responsive and unresponsive subjects, and healthy donors. (B) Heatmap of the average abundance of FMT response associated metabolites that differentiate the four groups. (C) PCA score plot of FMT response associated metabolites among FMT responsive and unresponsive subjects. (D) Disease enrichment analysis based on FMT response associated metabolites.

After FMT treatment, the metabolic phenotype of UC subjects gradually closed to the donor-like characteristic (Figure S9). Based on the algorithm of linear regression combined with exhaustive calculation, 41 metabolites were further identified to be relevant with FMT therapy (Figure S10A-B, Table S5). Interestingly, 93% amino acids and 91% fatty acids presented recovery tendency

in UC FMT subject, but only 27.3% of bile acids were recovered (p-value > 0.05, relative to donors). This recovery of amino acids levels in FMT subjects indicated an increased amino acid absorbance in the intestine, which may further suppress the release of colonic proinflammatory mediators, mitigated inflammation-induced increase of dendritic cells and T cells, and inhibited the Th1/Th17 responses in colonic mucosal lamina propria.[13] Recovered bile acid was inclusive of omega-muricholic acid, glycohyodeoxycholic acid, ursodeoxycholic acid, hyodeoxycholic acid, isolithocholic acid and nor cholic acid. Among them, ursodeoxycholic acid was reported to have anti-inflammatory functions[14], and can promote colonic epithelial wound healing[15]. Based on the above evidence, the 41 metabolites were subsequently used to validate FMT efficacy response, and the result showed good characteristic separation of FMT unbenefited patients from the FMT benefited subjects (Figure S10B-C). In order to confirm the universal dysbiosis of these 41 metabolites occurred in IBD patients, we then used the enrichment analysis of disease signatures based on the MetaboAnalyst and HMDB database. As shown in Figure S10D, these metabolites were significantly enriched in IBD or IBD-related diseases, including CD, IBS and UC, with p-value < 0.05.

S4. Inflammation-driven microbe-metabolite network interaction of UC patients.

To clarify the potential associations between microbiota dysbiosis and metabolite disorders, we subsequently performed the functional enrichment analysis of these dysfunctional microbes and metabolites. For dysfunctional metabolites, 25 enriched KEGG pathway was identified. Among them, bile acid biosynthesis, linolenic metabolism, and amino acid (glycine and serine) metabolism pathway are the most significant categories with p-value lower than 0.01 (Figure S11B). For dysfunctional microbes, 7 KEGG categories were successfully identified, including cellular processes, environmental information processing, genetic information processing, human diseases, metabolism, organismal systems, and unclassified (Figure S11A). Within these 7 KEGG categories, energy metabolism and amino acid metabolism was significant enrichment, in accordance with the above differentially enriched KEGG metabolic pathways.

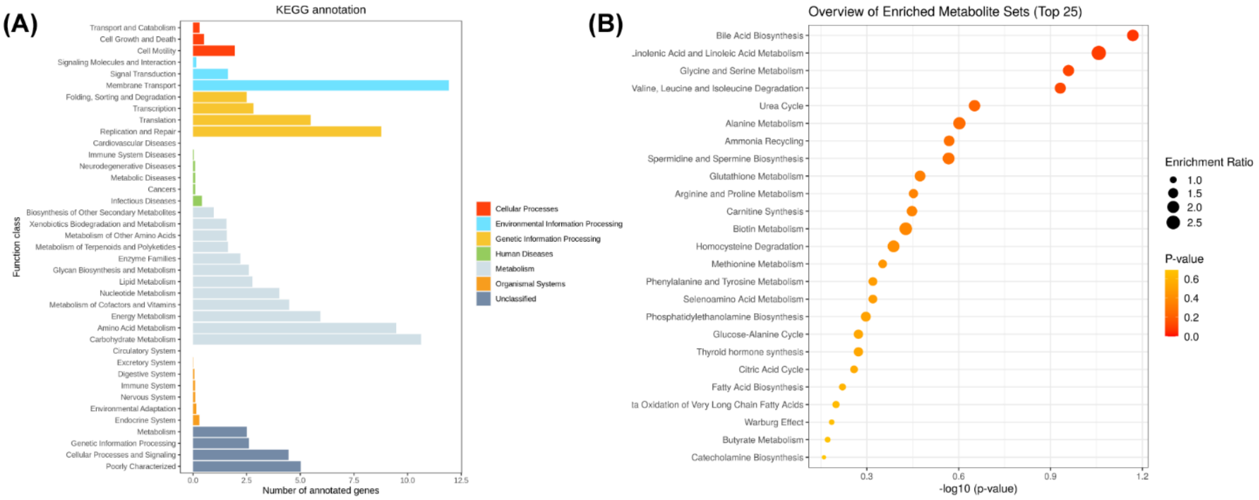

Figure S11. KEGG function analysis of (A) microbiota and (B) UC FMT responsive metabolites.

Then, the Spearman correlation analysis was performed to discover potential microbe-metabolite interaction. Herein, we used calprotectin, CRP and MAYO scores as prognostic indicators to correlate FMT responsive microbes and metabolites, and a totally of 41 metabolites and 23 microbes were involved in this network (Figure S12). These UC prognosis markers, microbes, metabolites, as well as associations between them were core indicators that correlated with the efficacy of FMT therapy and may as match indicators for FMT donor matching.

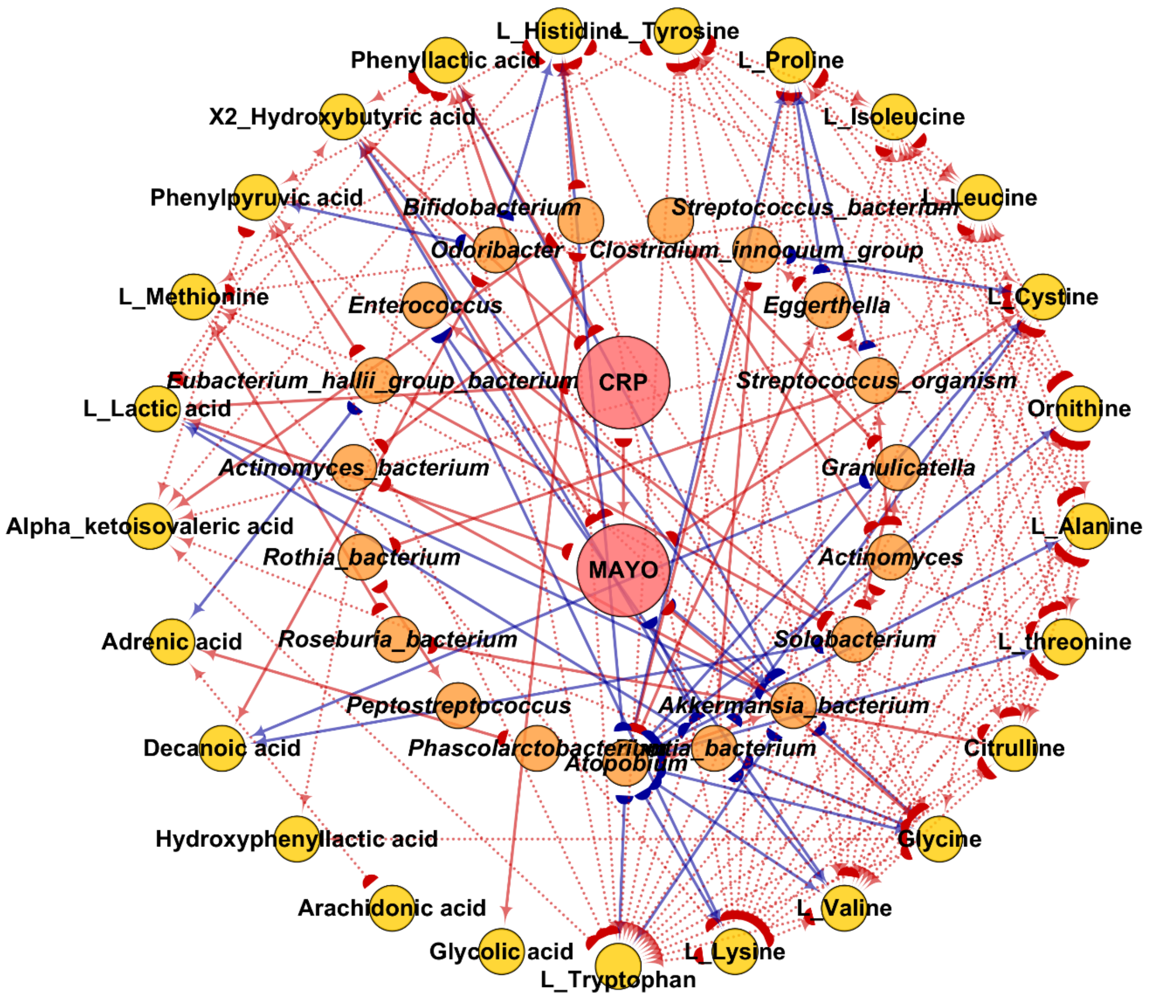

Figure S12. Spearman correlation network among FMT efficacy associated microbes, metabolites, and subjects' clinical indicators.

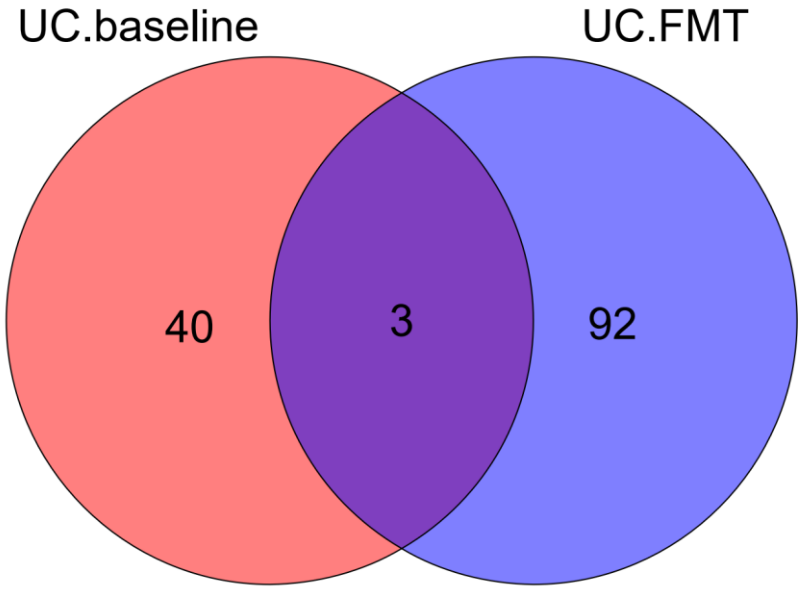

Figure S13. Venn diagram of Spearman correlation pairs (after excluding correlation between metabolites) among UC baseline and UC FMT subjects.

S5. Trajectory of the microbiome of CD patients receiving FMT.

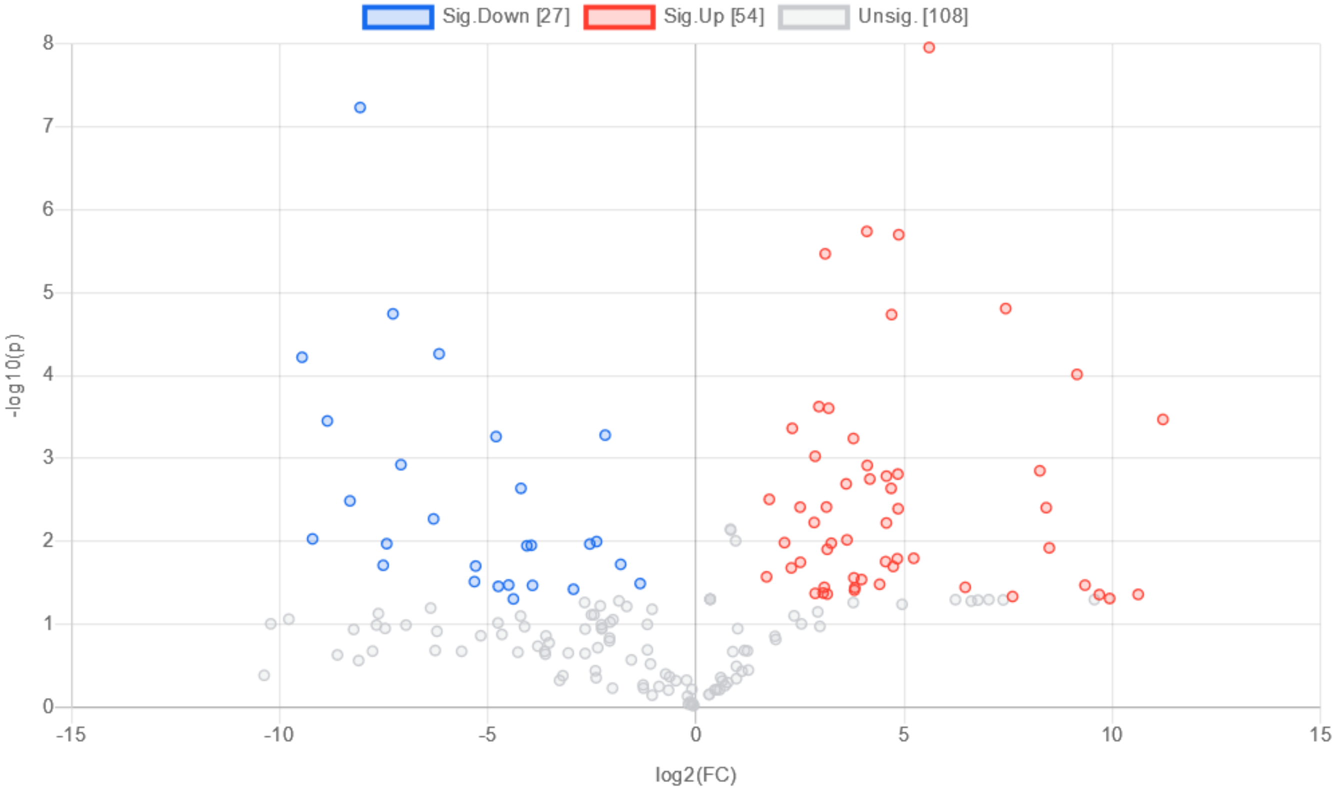

Figure S14. Volcano plot of microbe abundance between CD baseline subjects and donors. Legend: sig. down, downregulated microbe of CD baseline subjects relative to donors; sig. up, upregulated microbe of CD baseline subjects relative to donors; unsig, no significant difference between CD baseline and donors.

Referring to the analysis strategy of the UC cohort, 57 and 26 microbes were firstly identified up- and down-regulated in CD baseline, respectively (Figure S14 and Table S6). Among them, *Firmicutes* account for a 54.4% proportion in CD differentially abundant microbes. Moreover, CD specifically depleted microbes almost 88.5% also were *Firmicutes*.

On the other hand, α diversity indexes of ACE, Chao I and OTUs in CD baseline were significantly lower than donor level, and Simpson index was significantly higher in CD baseline (p-value < 0.05) (Figure S15). Via FMT treatment, these four α diversity indexes in FMT responsive subjects were all recovered to donor level (p-value > 0.05). However, like FMT-unresponsive subjects in the UC cohort, the FMT-unresponsive subjects in the CD cohort also showed the highest α diversity indexes of ACE, Chao I and OTUs.

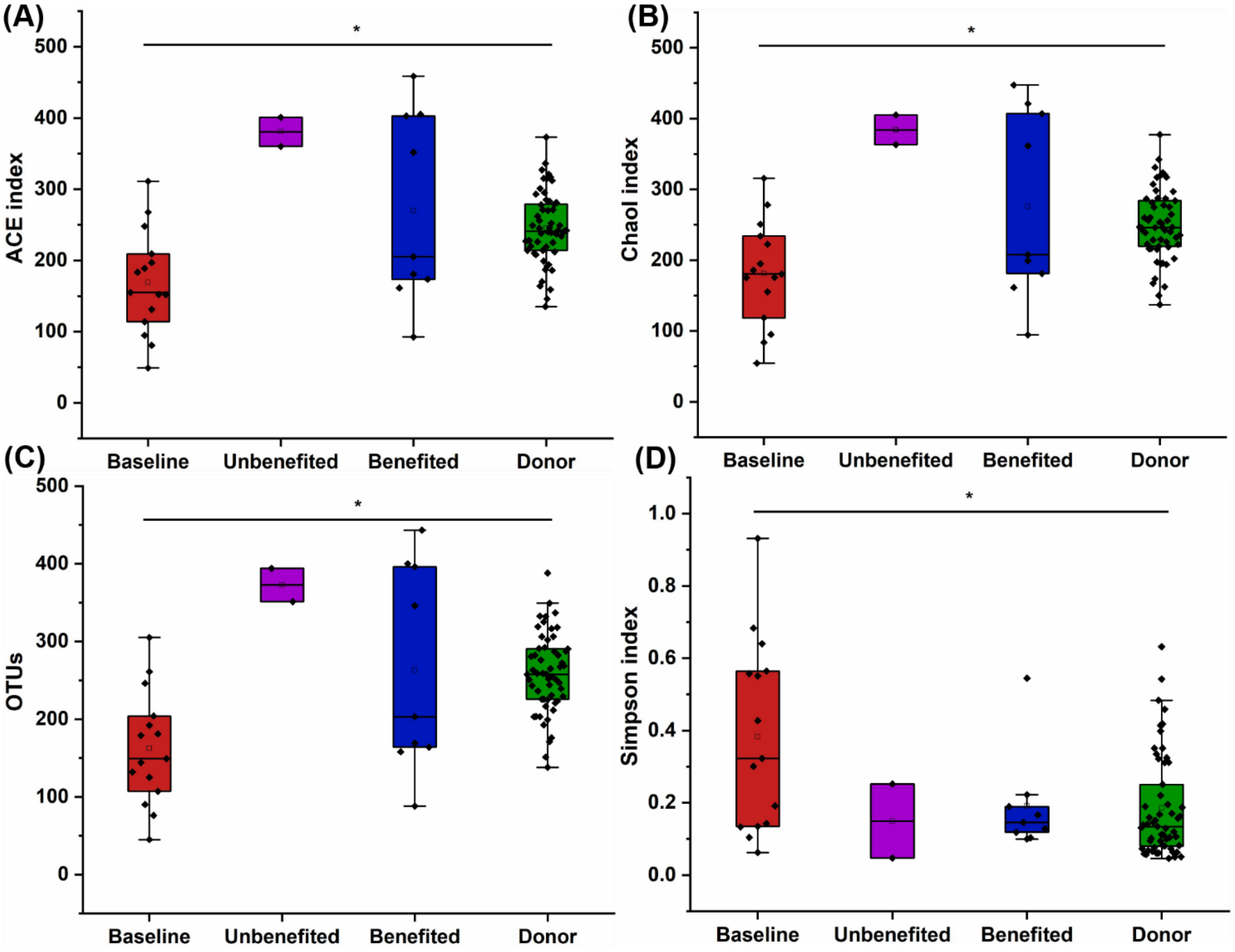

Figure S15. The α-diversity indexes of (A) ACE index, (B) Chao I index, (C) OTUs and (D) Simpson index among CD baseline subjects, FMT responsive and unresponsive subjects, and healthy donors.

Regarding to taxonomic differences between CD and donor subjects, totally 36 discriminatory OTUs were identified as discriminating blocks with threshold of LDA scores (log10) = 4 (Figure S16). Similar to UC results, opportunistic pathogens of *Enterobacteriaceae*[1] and *Gammaproteobactiria*[1] were also significantly enriched in CD baseline group, which were common features of UC and CD baseline. Dissimilarly, *Escherichia_Shigella* and *Enterococcus* were enriched in CD baseline, which was a general feature in previously reported IBD cases.[16] Furthermore, beneficial bacteria of *Roseburia*[4] was decreased in CD baseline, indicating a potential immune regulation disorder.[5-7]

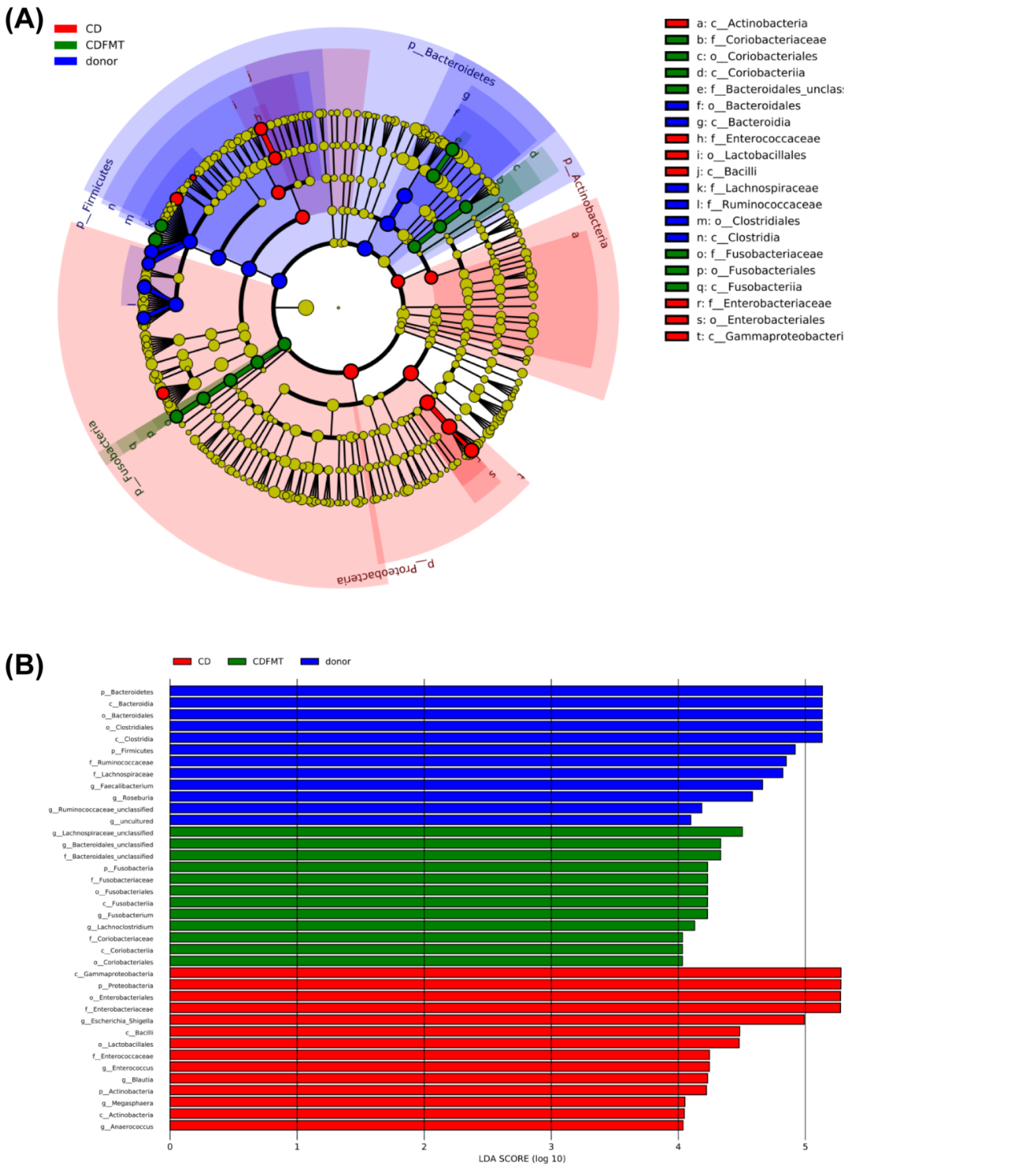

Figure S16. Linear discriminant analysis (LDA) integrated with effect size (LEfSe). (A) Cladogram indicating the phylogenetic distribution of microbiota correlated with CD patients (before and after FMT treatment) and donors. (B) The microbiota abundance differences among CD patients (before and after FMT treatment) and donors.

Via FMT treatment, *Bacteroidetes* phylum increased and *Proteobacteria* decreased in CD subjects, but other abnormally enriched bacteria phyla were still maintained in CD baseline level. More

generally, the parts of opportunistic pathogens were successfully decreased, but *Fusobacterium* was still significant enrichment.

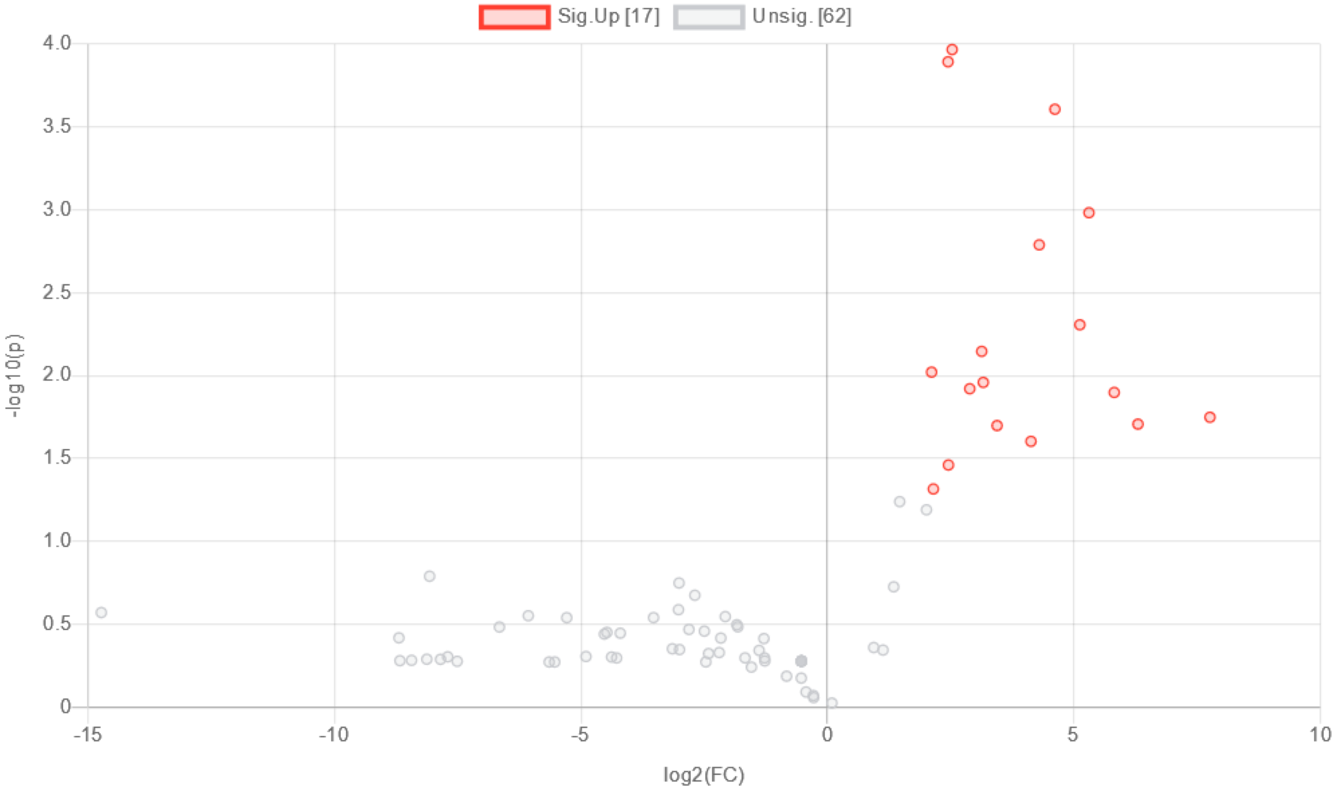

Figure S17. Volcano plot of microbe abundance between FMT responsive and unresponsive subjects. Legend: sig. up, upregulated microbe of FMT unresponsive patients relative to FMT responsive patients; unsig, no significant difference between FMT responsive and unresponsive subjects.

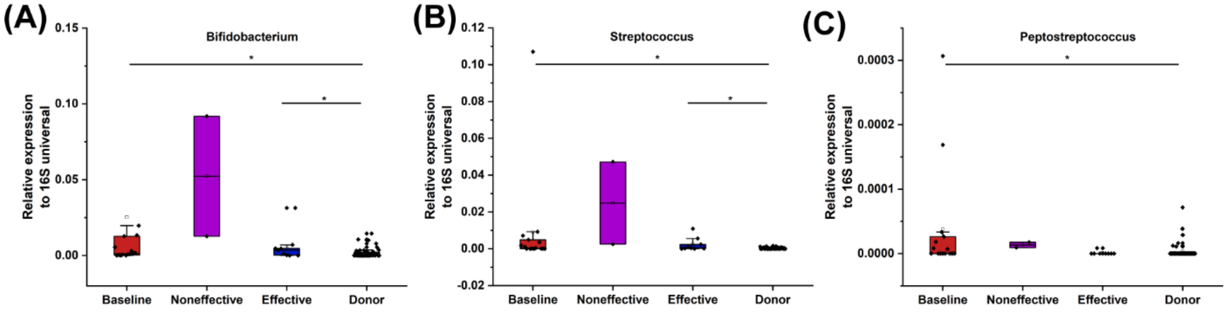

Figure S18. (A-C) The relative abundance of FMT efficacy-associated microbes among CD baseline, FMT responsive/unresponsive patients, and healthy donors.

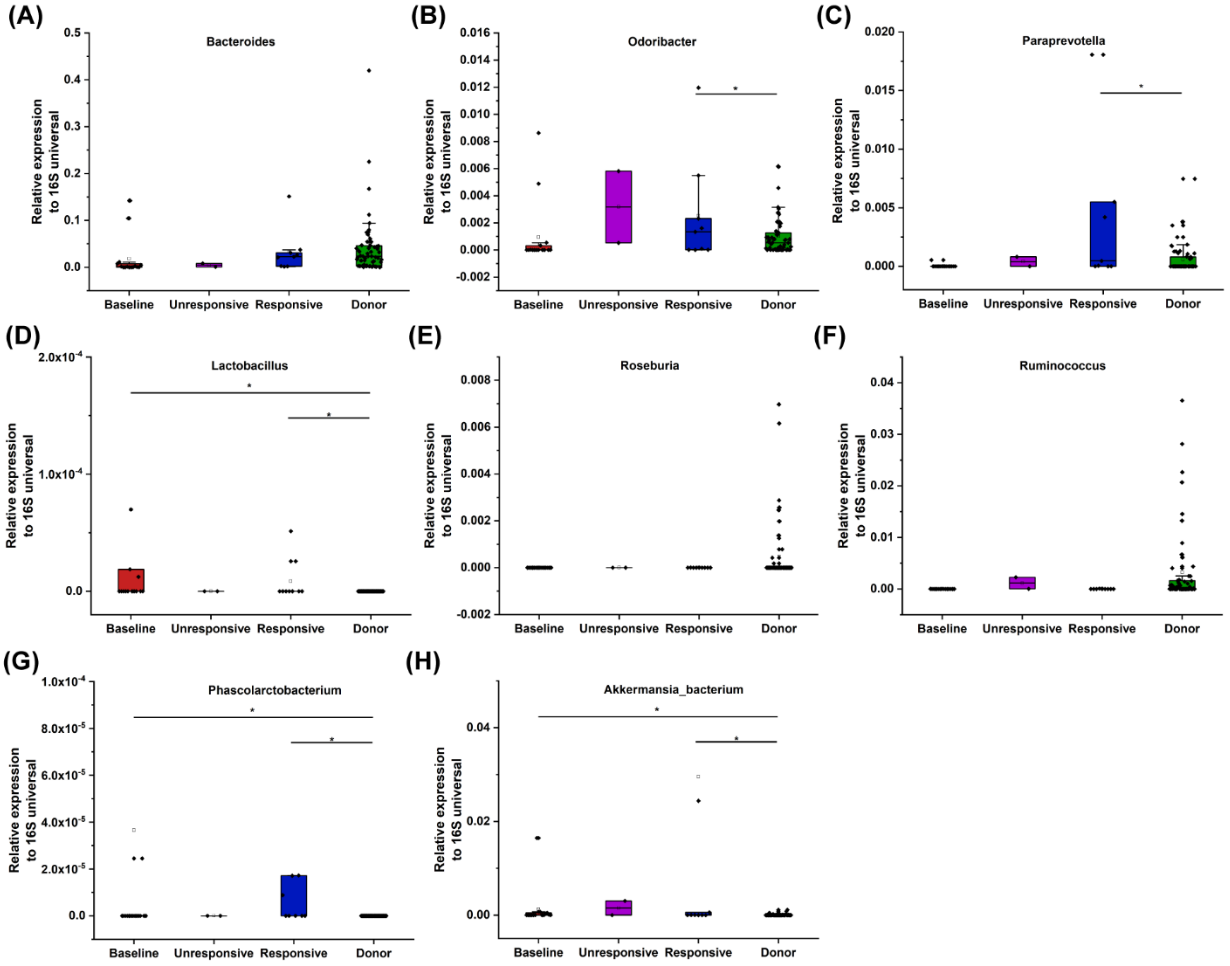

Figure S19. (A-H) The relative abundance of reported CD prognosis-associated microbes among CD baseline, FMT responsive/unresponsive patients, and healthy donors.

For identifying the FMT efficacy-associated microbes, we next performed volcano plot analysis between CD patients with or without FMT response (Figure S17). Eventually, 17 microbes, including 2 *Actinobacteriota* (*Bifidobacterium* and *Collinsella_bacterium*), 2 *Bacteroidota* (*Porphyromonas_bacterium*, *Prevotella_9_bacterium*) and 13 Firmicutes (*Streptococcus*, *Lachnospiraceae_ND3007_group_bacterium*, *Lachnospiraceae_NK4A136_group_bacterium*, *Lachnospiraceae_NK4A136_group_organism*, *Ruminococcus_torques_group*, *Monoglobus_bacterium*, *Butyricicoccus_bacterium*, *Faecalibacterium_bacterium*, *Ruminococcus_bacterium*, *Subdoligranulum_bacterium*, *Family_XIII_AD3011_group_bacterium*, *Peptostreptococcus* and *Dialister_organism*) were found enrichment in the FMT unresponsive subjects. Among them, only 3 microbes were enriched in the CD baseline and decreased to the donor level after FMT treatment, which was *Bifidobacterium*, *Streptococcus*, and *Peptostreptococcus* (Figure S18). *Bifidobacterium* and *Streptococcus* were recently reported have positive correlation with fecal calprotectin (an important IBD inflammation marker) in IBD pregnant women patients.[17] *Peptostreptococcus* was the target of immunoglobulin, which abundance increase may indicate an increased intense inflammatory response in intestine.[18] Traditional CD-related pathogenic microbes were also examined among CD baseline, CD FMT and donor subjects, which was inclusive of *Bacteroidota* genera of *Bacteroides*, *Odoribacter*, *Paraprevotella*; *Firmicutes* genera of *Lactobacillus*, *Roseburia*, *Ruminococcus*, *Phascolarctobacterium*; and *Verrucomicrobiota* genera of *Akkermansia_bacterium*. Among them, *Lactobacillus* was significantly

enriched in the CD baseline, *Bacteroides*, *Odoribacter*, *Paraprevotella*, *Roseburia*, and *Ruminococcus* were significantly decreased in the CD baseline (Figure S19).

S6. Host-microbe co-metabolites disordered in CD patients.

For metabolome in CD cohort, PCA score plot showed a distinct difference between donor and CD baseline (Figure 3C). Moreover, baseline, FMT responsive and unresponsive subjects also showed recognizable differences. PLS-DA was firstly used to find CD associated metabolites relative to donors, and the score plot shown great separation of them, without any overlap of 95% confidence interval (Figure S20A). The R^2 and Q^2 of the PLS-DA model were 85% and 75%, respectively. PLS-DA VIP plot showed 58 contributed metabolites relevant to CD phenotype discrimination (Figure S20B and Table S7). These CD differentially changed metabolites contained 17 amino acids, 15 bile acids, 10 fatty acids, 9 organic acids, and 7 other class metabolites. Overall, amino acids metabolites were abundant but bile acids and fatty acid metabolites were depleted in CD baseline patients (Figure S20C). Similar to UC cases, organic acids were not found consistent trends among CD case-control study. Unlike UC cases, the CD metabolome presented more dysregulated metabolites in bile acid classes, including primary bile acid of hyocholic acid, glycocholic acid and clycochenodeoxycholic acid; and secondary bile acid of lithocholic acid, deoxycholic acid, glycolithocholic acid, etc. These dysfunctions of bile acid classes were in agreement with previous findings,[8, 19] and may associate with aggravation of diarrhea symptom.[20, 21] Via FMT treatment, these differentially changed metabolites was gradually recovered to donors' characteristic (Figure S21). Then, based on the algorithm of linear regression combined with exhaustive calculation, 50 metabolites of them were eventually identified to be relevant with FMT-therapy (Figure S22A-B, Table S8). Based on the above evidence, these 50 metabolites were further used to validate FMT efficacy response, and the result showed good characteristic separation of FMT unbenefited patients from FMT benefited subjects (Figure S22B-C). In order to demonstrate the representativeness of these CD prognosis-related metabolites, we then performed the enrichment analysis of disease signatures based on the MetaboAnalyst and HMDB database. As shown in Figure S22D, these metabolites were significantly enriched in IBD or IBD-related diseases, including CD, IBS and rheumatoid arthritis, with $p\text{-value} < 0.05$.

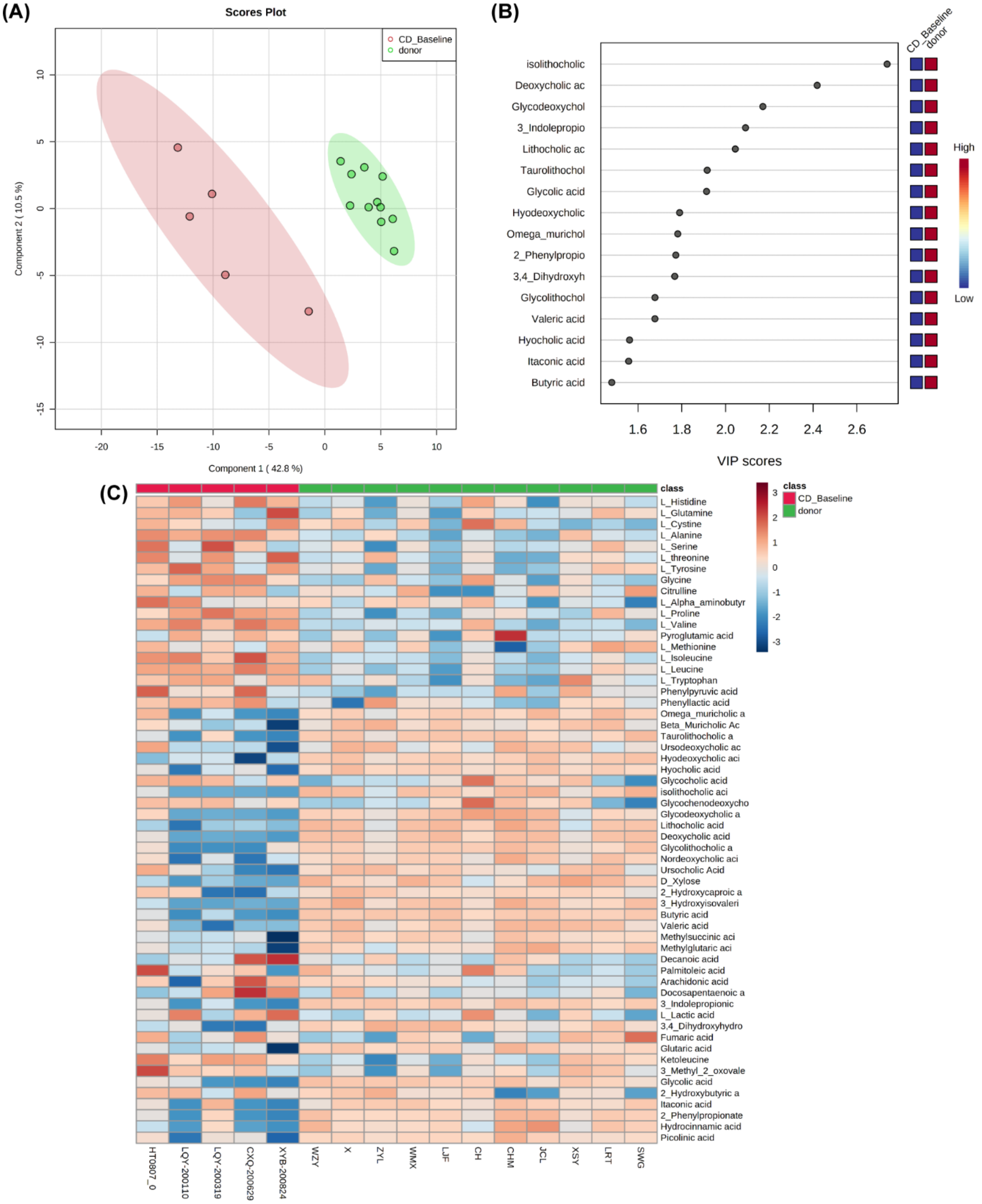

Figure S20. Metabolome difference between CD baseline subjects and donors. (A) PLS-DA score plot of metabolome between CD baseline subjects and donors. (B) Top 16 of key metabolites with VIP value more than 0.9. (C) Heatmap of 58 CD differentially expressed metabolites.

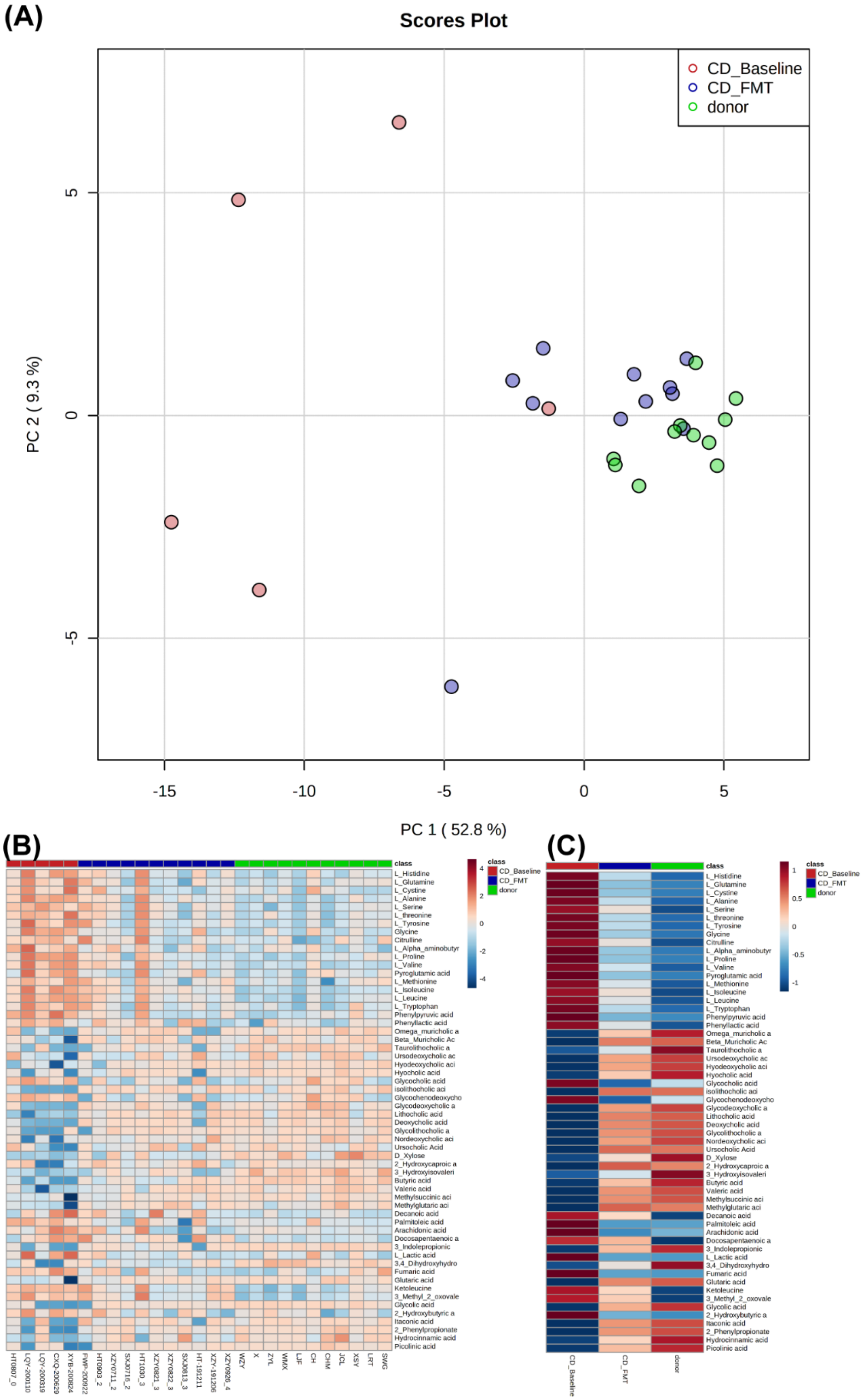

Figure S21. Metabolome difference among CD baseline, CD FMT and donor subjects. (A) PCA score plot of CD differentially expressed metabolites among CD baseline, CD FMT and donor subjects. Corresponding (B) heatmap and (C) mean-value heatmap among these three groups.

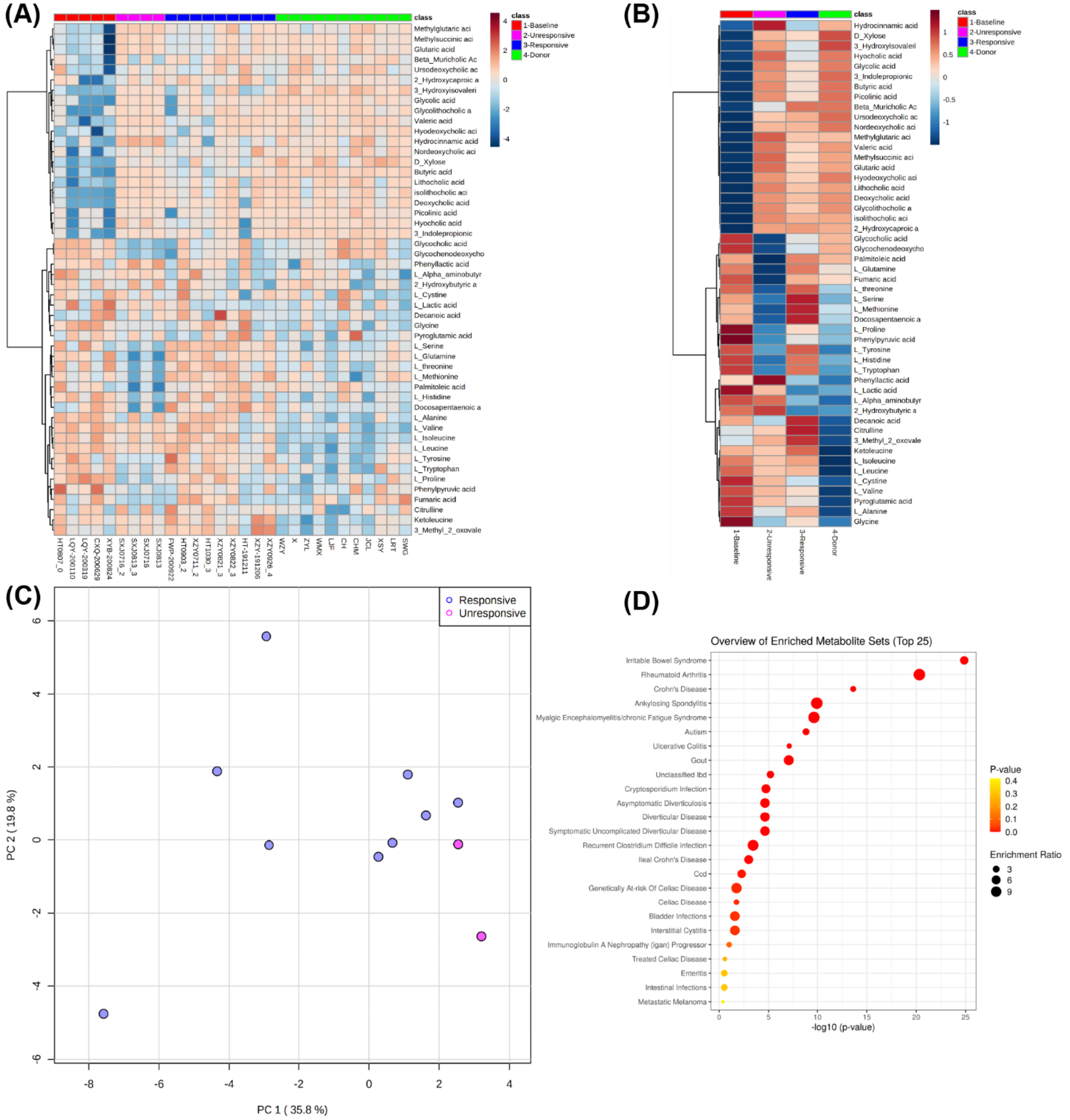

Figure S22. (A) Heatmap of the expression of FMT response associated metabolites that differentiate among CD baseline subjects, FMT responsive and unresponsive subjects, and healthy donors. (B) Heatmap of the average expression of FMT response associated metabolites that differentiate the four groups. (C) PCA score plot of FMT response associated metabolites among FMT responsive and unresponsive subjects. (D) Disease enrichment analysis based on FMT response associated metabolites.

To clarify the potential relationship between microbiota dysbiosis and metabolite disorders, we subsequently performed functional enrichment analysis of CD dysfunctional microbiota and metabolites. For

dysfunctional metabolites, bile acid biosynthesis, urea cycle, and ammonia recycling pathway were the most significant categories with p-value lower than 0.01 (Figure S23). For dysfunctional microbiota, 7 KEGG categories were successfully identified, including cellular processes, environmental information processing, genetic information processing, human diseases, metabolism, organismal systems, and unclassified.

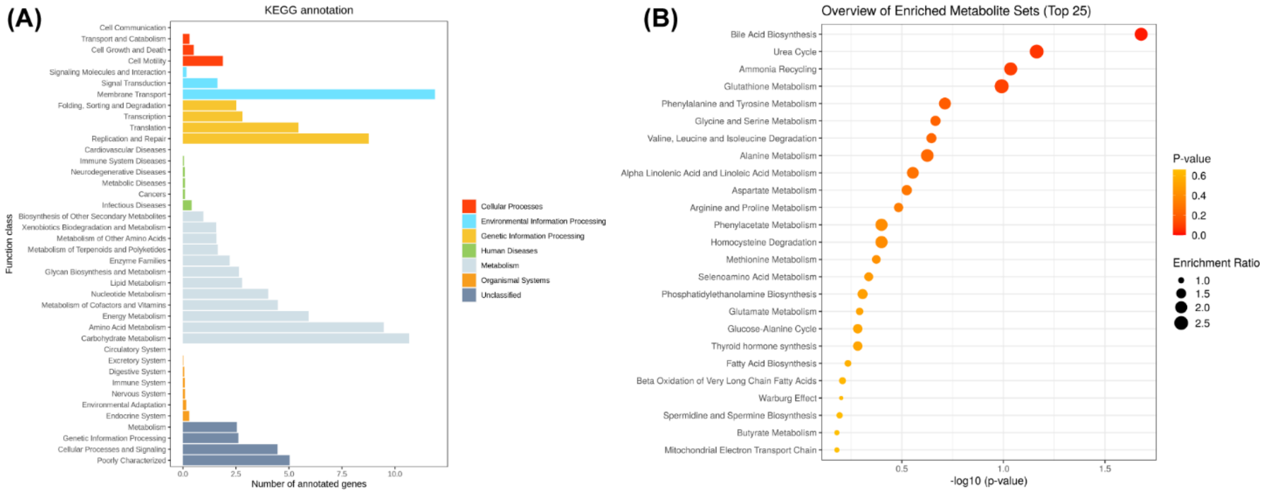

Figure S23. KEGG function analysis of (A) microbiota and (B) CD FMT responsive metabolites.

S7. Inflammation-driven microbe-metabolite network interaction of CD patients.

Then, an inflammation-driven microbe-metabolite correlation network was constructed by using inflammation indicators of patients as association nodes (Figure S24). To further verify the practicability of this inflammation-driven microbe-metabolite correlation network, ternary Spearman networks were constructed in CD baseline and CD FMT subjects, respectively. Only 92 correlation pairs were found in CD baseline subjects, and 11 correlation pairs were remained after excluding correlation between metabolites. On the contrary, CD FMT group had 345 correlation pairs, and 45 correlation pairs were remained after excluding correlation between metabolites. Meanwhile, all of these baseline-specific correlation pairs (n=11) were replaced by FMT-induced correlation after FMT treatment, which was coincident with UC FMT cases (Figure S25), indicating that FMT induced the substantial change of microbes-metabolites interaction network in the recipient’s intestine.

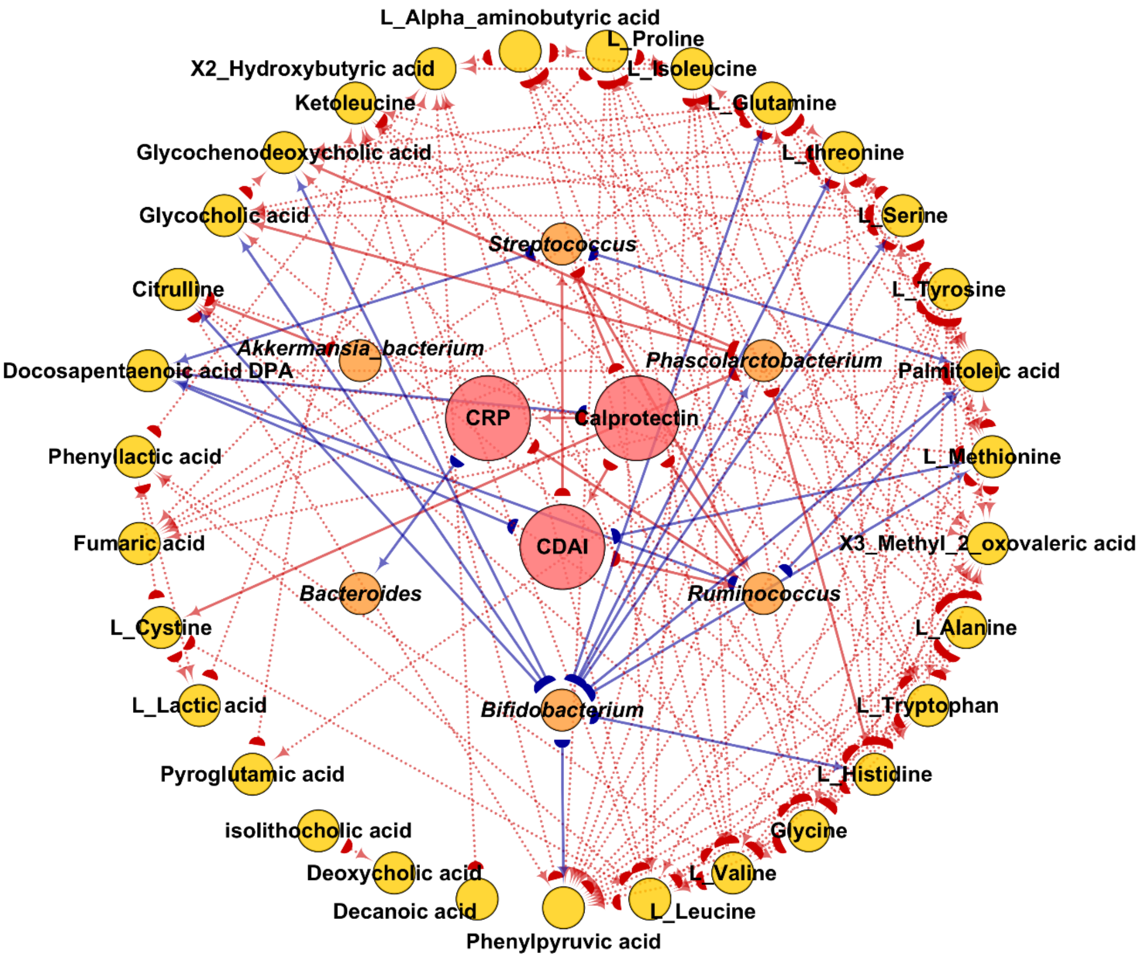

Figure S24. Spearman correlation network among CD FMT efficacy associated microbes, metabolites and subjects' clinical indicators.

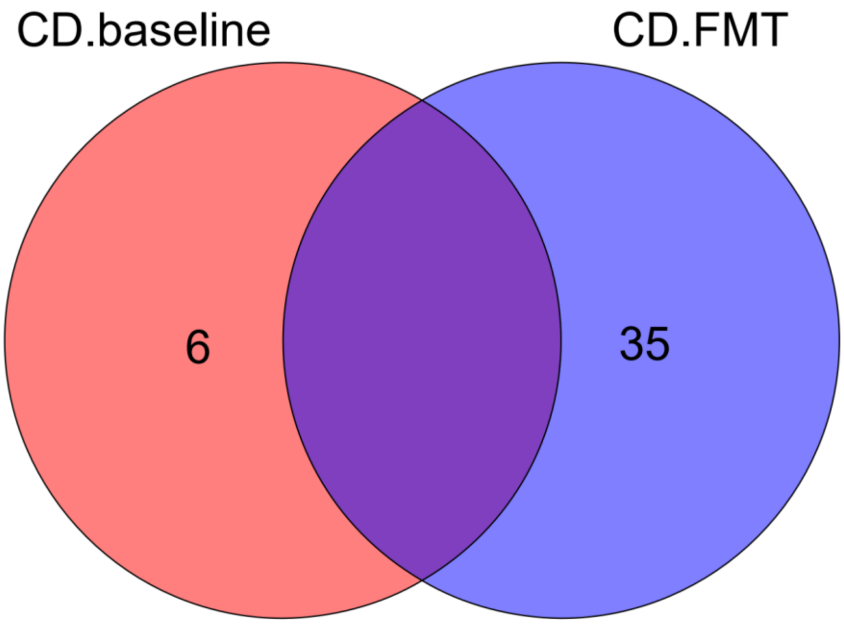

Figure S25. Venn diagram of Spearman correlation pairs (after excluding correlation among metabolites) among CD baseline and CD FMT subjects.

S8. Intervention effect on intestinal microbiota diversity by FMT or IFX treatment can be inherited by IFX-FMT combination treatment.

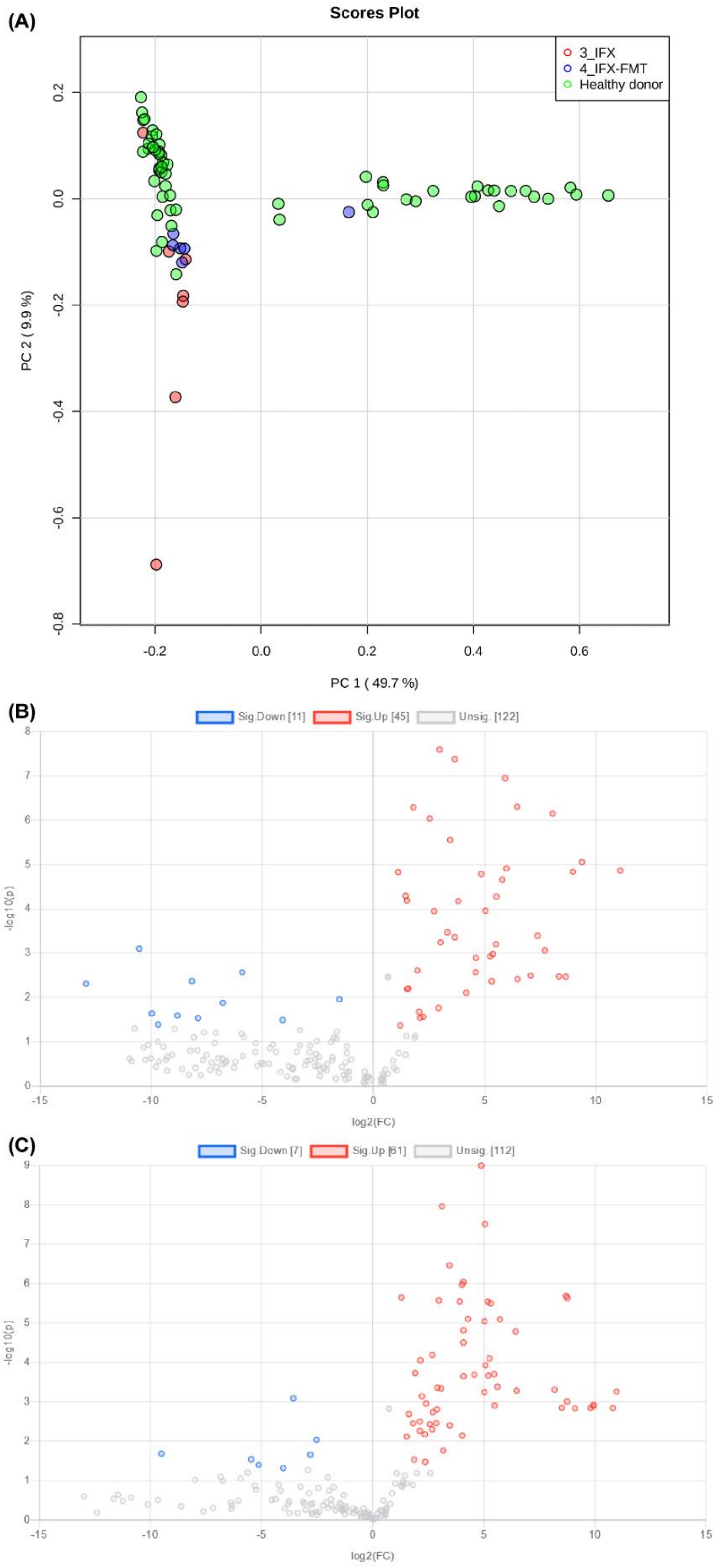

Figure S26. (A) PCA score plot of microbiome between CD IFX subjects, IFX-FMT subjects and donors. (B) Volcano plot of microbe abundance between IFX subjects and donors. (C) Volcano plot of microbe abundance between IFX-FMT subjects and donors. Legend: sig. up, upregulated microbes; sig. down, downregulated microbes; unsig, no significant difference.

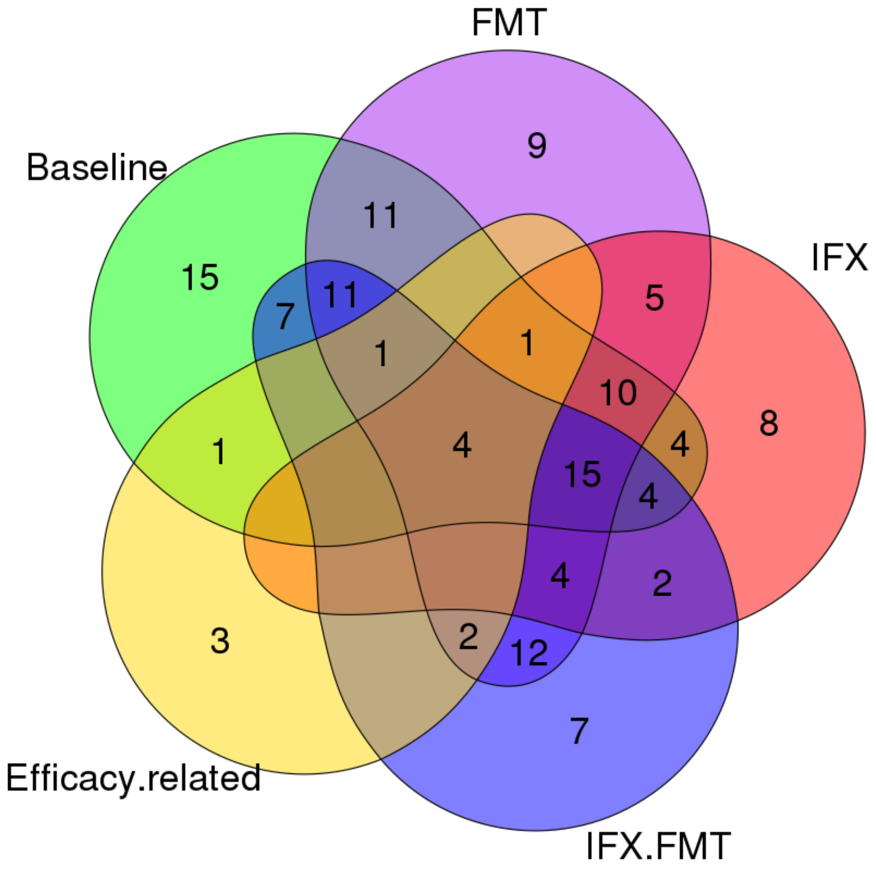

Figure S27. Venn diagram of characteristic microbes among baseline, IFX, FMT, IFX-FMT subjects.

FMT unbenefited CD patients gradually remitted after receiving FMT-IFX combined treatment (Figure 1). In order to fully elucidate the microbiota changes in this process, we respectively performed volcano plot analysis to obtain IFX subjects and IFX-FMT subjects differentially changed microbes relative to donors (Figure S26). For IFX subjects, 45 and 11 microbes were identified up- and down-regulated in them (Table S9). It is important that all IFX subjects-depleted microbes (n=11) and 58% of IFX subjects-enriched microbes (n=26) were in agreement with CD baseline cases. For IFX-FMT subjects, 61 microbes were up-regulated and 7 microbes were down-regulated in them (Table S10). Similarly, all depleted microbes (n=7) and 48% of enriched microbes (n=29) were CD baseline associated microbes. Taking the CD-associated microbes as a reference, we found that 91% of characteristic microbes were still dysbiosis after FMT treatment (53/58), 66% after IFX treatment (38/58) and 72% after IFX-FMT combined treatment (42/58) (Figure S27). Meanwhile, taking FMT response associated microbes as a reference, we also found IFX therapy had optimal results (Figure S27). Nevertheless, in terms of overall microbiota characteristics, patients treated with IFX-FMT combined therapy were more similar to the microbiome phenotype of donors than those

treated with IFX alone (Figure S26A). Moreover, pure IFX treated CD patients presented overall depletion in CD efficacy associated microbes, except for *Bifidobacterium*, and *Akkermansia_bacterium* (Figure 4A). This depletion can be partly inherited by IFX-FMT group, inclusive of microbes of *Roseburia*, *Ruminococcus*, *Lactobacillus*, and *Peptostreptococcus*. In contrary with IFX therapy, FMT therapy presented comprehensive microbiota interventions, especially in the recovery of *Bacteroides*, *Odoribacter* and *Paraprevotella* (Figure 4A). These characteristics of FMT therapy also can be inherited by FMT groups. More generally, *Bifidobacterium* and *Streptococcus* were CD baseline characteristically enriched genera, which could not be decreased to donor's level by FMT or IFX therapy, and also by FMT-IFX combined therapy (Figure S28A-B); *Peptostreptococcus* were CD baseline enriched microbe, which could be decreased to donor's level by IFX therapy and IFX-FMT combined therapy but not the pure FMT therapy (Figure S28C); *Lacbotacillus* was also the CD baseline abundant microbe, which could be decreased by FMT therapy, and decreased to donor's level by IFX-FMT therapy, but not be influenced by pure IFX therapy (Figure S28G). For CD baseline depleted genera, *Bacteroides* can be increased after FMT, IFX, or IFX-FMT treatment (Figure S28D); *Odoribacter* and *Paraprevotella* can be increased by FMT therapy and IFX-FMT combined therapy, but not significantly changed by pure IFX therapy (Figure S28E-F).

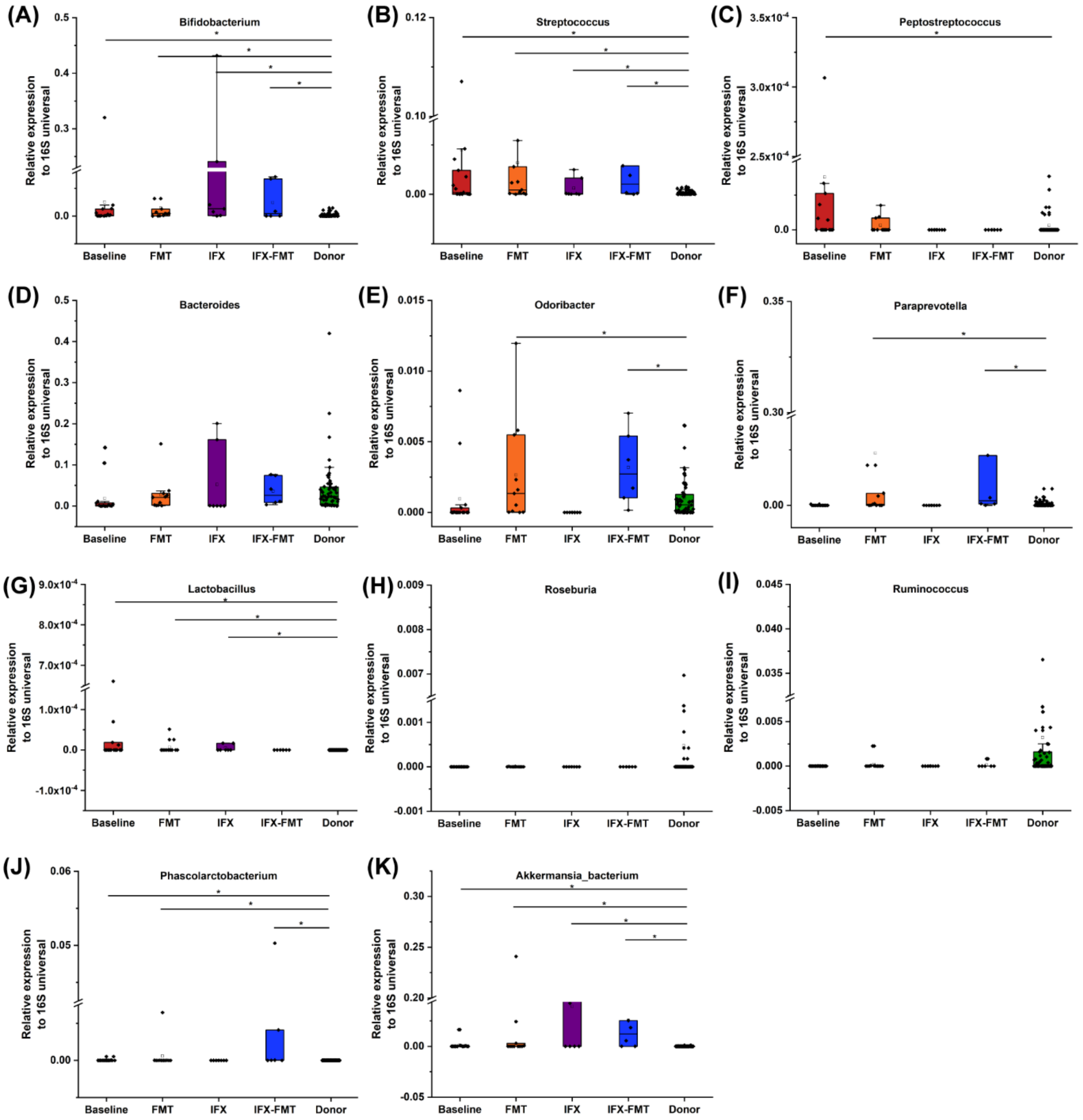

Figure S28. (A-K) The relative abundance of CD prognosis-associated microbes among CD baseline, FMT subjects, IFX subjects, IFX-FMT subjects and healthy donors.

S9. Analysis of failure cases in monotherapy.

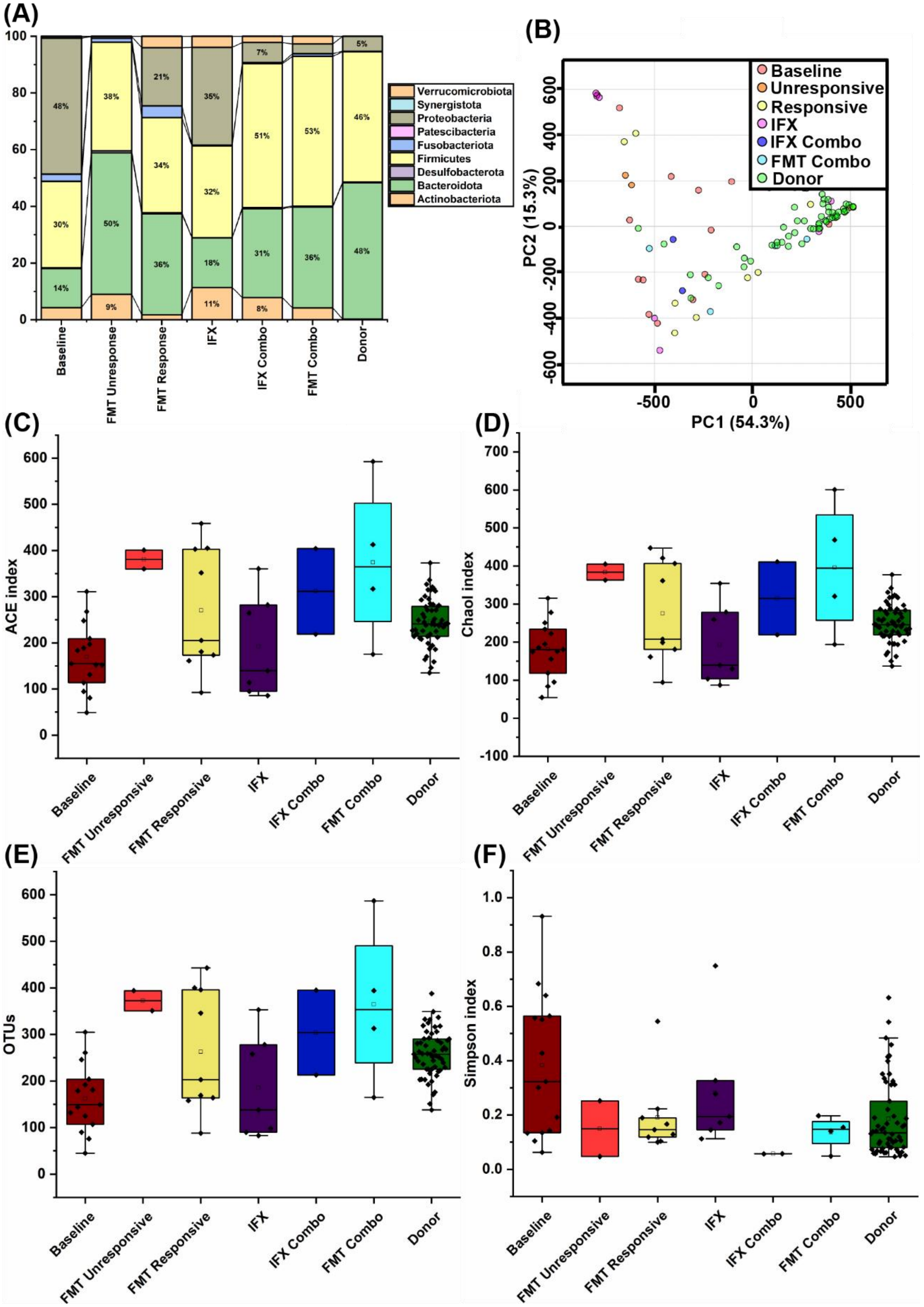

Figure S29. (A) Component proportion of bacterial phylum among the 7 groups. (B) PCA score plot of CD efficacy-related microbes among the 7 groups. (C-F) α-diversity indexes among the 7 groups.

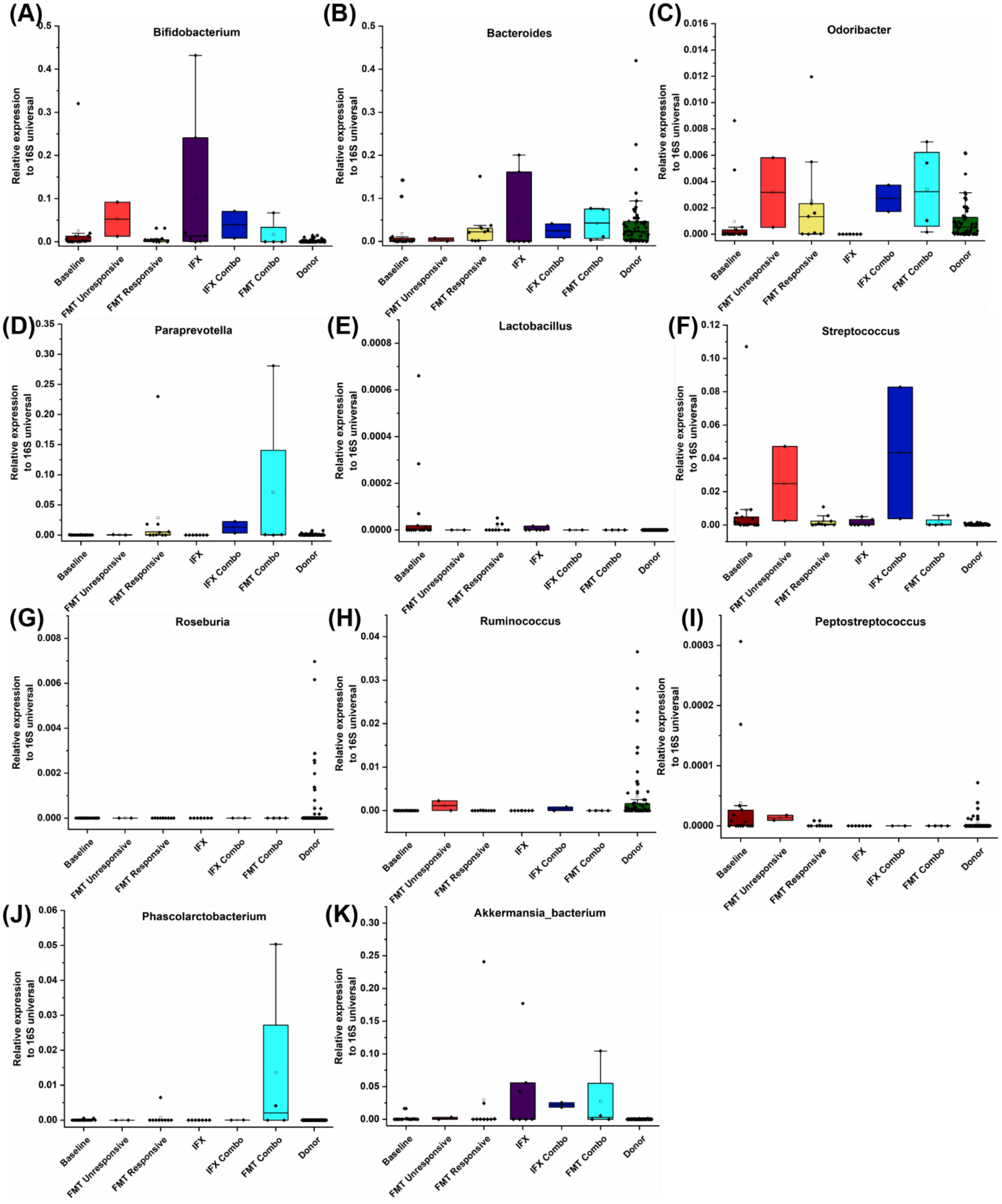

Figure S30. (A-K) The relative abundance of CD prognosis-associated microbes among the 7 groups.

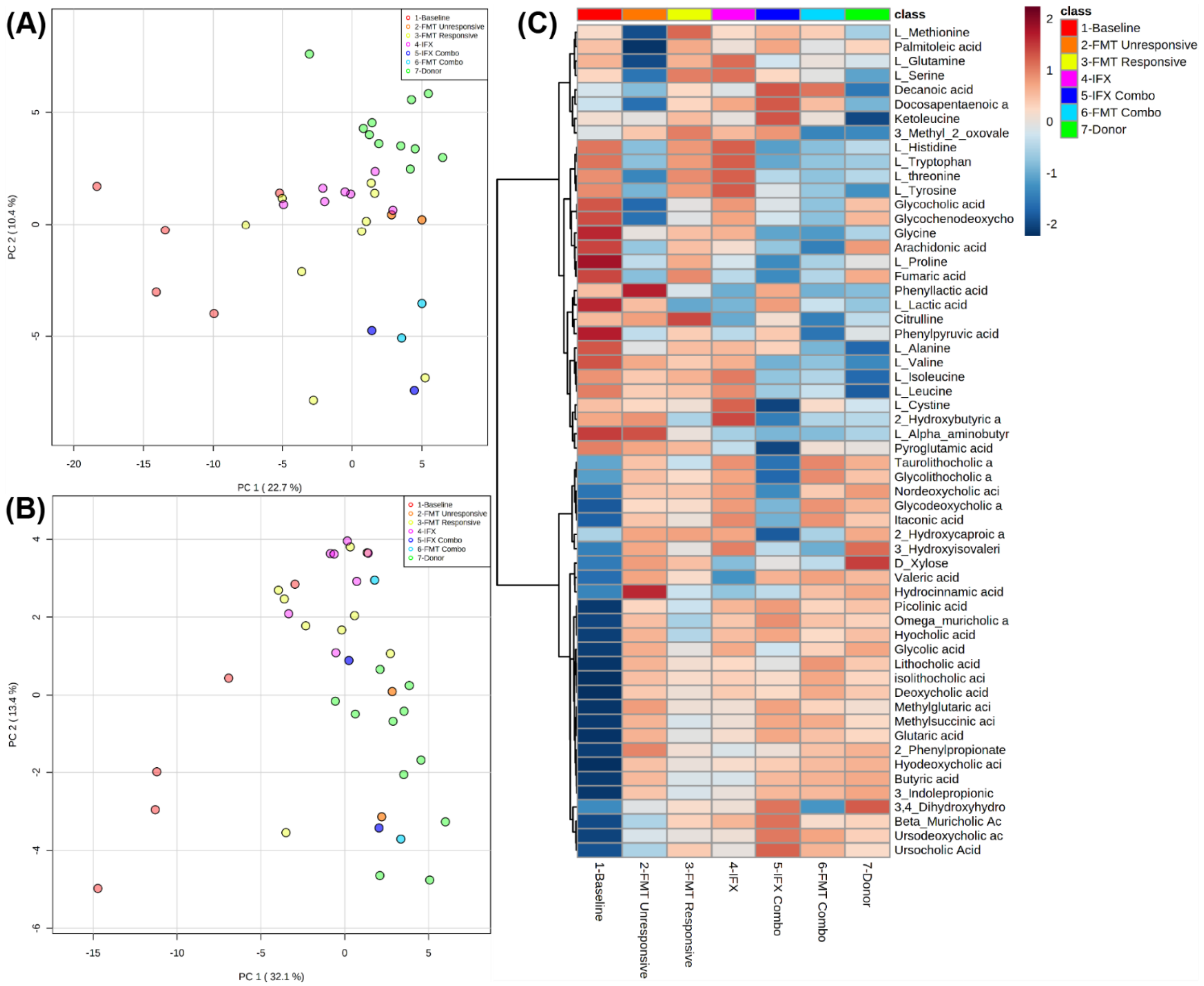

Figure S31. (A) The PCA score plot of metabolits among the 7 groups. (B) The PCA score plot of CD efficacy-related metabolits among the 7 groups. (C) Heatmap of mean of CD efficacy-related metabolits among the 7 groups.

For patients who failed in IFX treatment, we found that their proportion of Proteobacteria were abnormally elevated (up to 35%) relative to healthy donors (only 5%), and the pro-portions of Firmicutes and Bacteroidota were reduced to 32% and 18%, respectively (Figure S29). After the combined treatment of IFX and FMT, these abnormal bacteria phyla returned to the donor level. Specific to CD efficacy-related bacterial genera, we found that *Odoribacter* and *Paraprevotella*, the two CD-depleted microbes, are still depleted after IFX treatment, but their abundance increases after IFX-FMT combination treatment (Figure S30). For patients who failed in FMT treatment, we found their proportion of *Actinobacteriota* were increased (about 9%) and the proportion of *Proteobacteria* were reduced (< 1%) relative to healthy donors. After the combined treatment of IFX and FMT, these dysregulated bacteria phyla were im-proved. Peptostreptococcus was one of CD efficacy-related bacterial genera, which was enriched in CD baseline subjects and FMT unresponsive subjects, and decreased to donors' level after IFX-FMT combination treatment. The above-mentioned microbes may be markers of poor prognosis of monotherapy, and IFX-FMT combination therapy should be performed when these microbes are abnormal.

Metabolomics results also show that the order of IFX and FMT treatment is not essential to the final metabolic char-acteristics (Figure S31). For patients who have failed in IFX treatment, the concentration

of amino acid species (L-histidine, L-tryptophan, L-threonine, L-tyrosine, etc.) in their stool samples is higher, similar to the baseline level. After IFX-FMT combination therapy, the concentration of these metabolites returned to the donors' level. For patients who have failed in FMT treatment, some amino acids also show abnormally high concentrations, such as L-alanine, L-valine, L-isoleucine and L-leucine. After the IFX-FMT combined treatment, the concentration of these metabolites also returned to the donor level. Therefore, after monotherapy, if the amino acid species in the patient's stool is abnormally elevated, it should be transferred to IFX-FMT combination therapy as soon as possible.

S10. Intervention effect on host-microbe co-metabolism by FMT or IFX treatment also can be inherited by IFX-FMT combination treatment.

Heatmap of metabolome showed that IFX therapy, FMT therapy, and IFX-FMT combined therapy could improve CD subjects' metabolome even to donors' level (Figure 4F). Specifically, 58 metabolites were found differentially changed in CD baseline subjects relative to donors, but only 26 of them were maintained dysbiosis after FMT treatment, and similarly, 27 for IFX treatment and 15 for IFX-FMT combined treatment (Figure S32, Table S11 and Table S12). Especially, introducing FMT-response-related metabolites as a reference, only 11 metabolites were identified dysbiosis in IFX-FMT subjects, significantly lower than pure FMT subjects (n=23) and IFX subjects (n = 23) (Figure S32). More specifically, most of CD baseline depleted bile acid metabolites (except for glycocholic acid and glycochenodeoxycholic acid) can be recovered to donors' level via FMT therapy, and these effects can be inherited by IFX-FMT therapy except for taurolithocholic acid and glycolithocholic acid, which still depleted in IFX-FMT subjects; IFX therapy can improve all of depleted bile acid metabolites levels but not decrease the level of baseline-abundant bile acid metabolites (glycocholic acid and glycochenodeoxycholic acid); pure FMT or IFX therapy cannot decrease amino acid metabolites levels to donors' level, but IFX-FMT combined therapy has unique advantages in this aspect; IFX therapy seems cannot improve the depletion of D-xylose and valeric acid, but FMT therapy and IFX-FMT combined therapy not effect by this (Figure S33). In general, IFX-FMT combination therapy can inherit the advantages of pure FMT therapy and IFX therapy, and finally make the metabolomic characteristics of the treated patients closest to that of donor subjects.

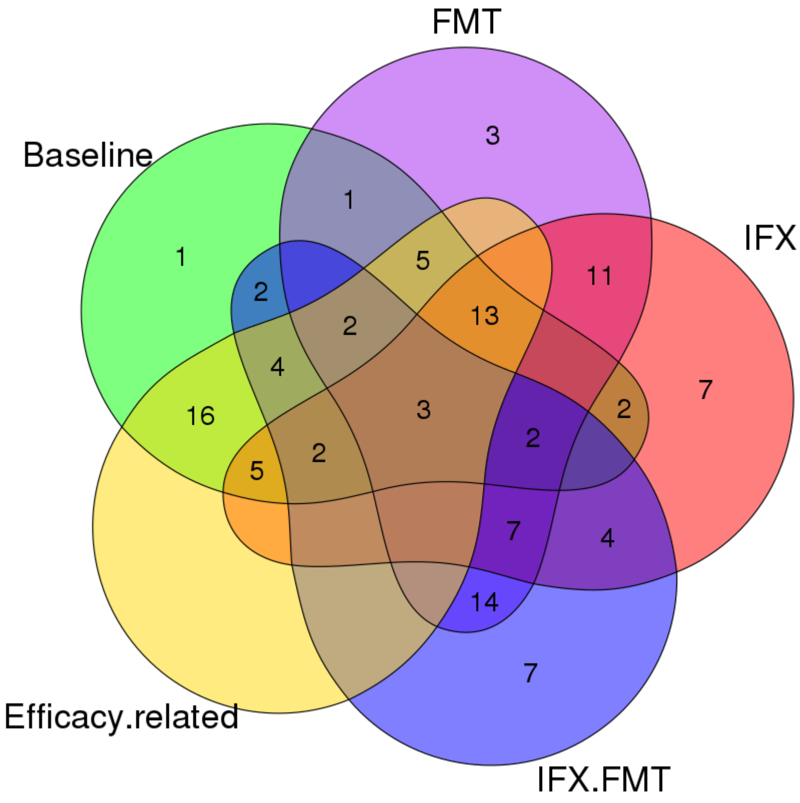

Figure S32. Venn diagram of characteristic metabolites among baseline, IFX, FMT, IFX-FMT subjects.

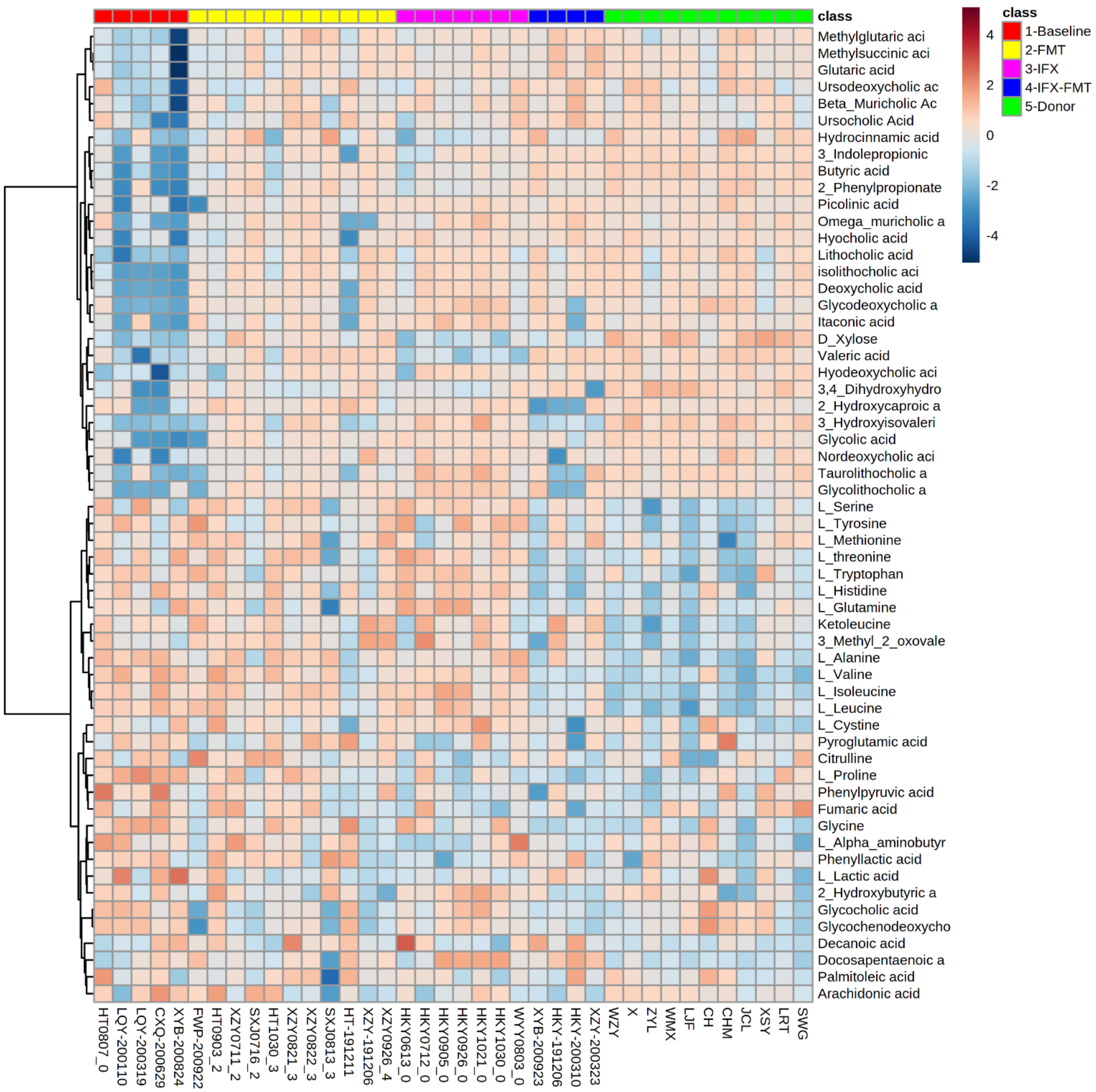

Figure S33. Heatmap of 58 CD differentially expressed metabolites among CD baseline, FMT subjects, IFX subjects, IFX-FMT subjects and donors.

S11. Annexed tables.

Table S1. Characteristics of patients include.

Patient	Disease duration (year)	Age at diagnosis	SEX	Disease location	Disease activity	Disease behavior	Extraintestinal manifestation	Concomitant treatment	Previous treatment	CRP (mg/dL)	Calprotectin protein (µg/g)	Clinical outcome
UC 1	3.3	20.5	m	E2	Mayo 5	-	N	-	5-ASA, GCS	3.02	>1800	R
UC 2	6.9	40.0	m	E3	Mayo 6	-	N	-	5-ASA, GCS	17.3	>1800	R
UC 3	6.4	48.7	m	E3	Mayo 9	-	N	GCS	5-ASA, AZA	>200	>1800	R
UC 4	13.5	54.4	f	E1	Mayo 7	-	Y	-	5-ASA	34.5	1350	R
UC 5	0.2	33.3	m	E2	Mayo 4	-	N	-	5-ASA	16.2	>1800	R
UC 6	3.1	19.2	f	E2	Mayo 4	-	N	-	5-ASA	9.2	>1800	R
UC 7	1.8	46.7	m	E2	Mayo 5	-	N	-	5-ASA, GCS	3.5	>1800	R
UC 8	20.5	23.2	f	E2	Mayo 5	-	N	-	5-ASA	6.5	>1800	R
UC 9	13.9	57.8	m	E2	Mayo 6	-	N	-	5-ASA, GCS	7.8	>1800	R
UC 10	8.5	56.3	m	E1	Mayo 4	-	N	-	5-ASA	6.22	1620	R
UC 11	7.5	17.6	m	E1	Mayo 2	-	N	-	5-ASA	3.02	876	R
UC 12	2.3	42.3	m	E2	Mayo 3	-	N	-	5-ASA, AZA	3.15	>1800	R
UC 13	4.0	60.6	m	E1	Mayo 3	-	N	-	5-ASA	3.07	1570	NR
UC 14	0.5	30.8	f	E1	Mayo 4	-	Y	-	5-ASA, GCS	3.02	1267	NR

UC 15	2.7	34.0	m	E3	Mayo 7	-	N	-	5-ASA	136.86	>1800	NR
CD 1	5.0	28.4	f	L1	CDAI 202	B1	N	-	5-ASA	<0.4	492	R
CD 2	0.5	36.2	m	L1	CDAI 184	B1	N	-	5-ASA	7.79	494	R
CD 3	0.8	31.8	f	L3	CDAI 365	B2	N	EEN	5-ASA, AZA	2.32	>1800	R
CD 4	1.1	26.3	m	L3	CDAI 321	B2	N	EEN	5-ASA	>200	>1800	R
CD 5	0.4	29.5	m	L1	CDAI 289	B2	Y	EEN	5-ASA	12.5	210	R
CD 6	5.1	25.1	m	L1	CDAI 169	B2	N	EEN	5-ASA	5.6	210	R
CD 7	0.9	33.9	m	L1	CDAI 221	B2	Y	EEN	5-ASA	3.5	465	R
CD 8	1.8	34.7	f	L3	CDAI 287	B1	Y	-	5-ASA	13.0	>1800	R
CD 9	1.0	24.3	f	L1	CDAI 210	B2	N	EEN	5-ASA, EEN	3.02	526	R
CD 10	1.5	23.6	f	L3	CDAI 321	B1	N	-	5-ASA	99.6	>1800	NR
CD 11	2.0	27.4	f	L1	CDAI 275	B2	N	EEN	5-ASA, AZA	3.78	909	NR
CD 12	7	31.2	m	L1	CDAI 290	B1	N	-	5-ASA	25.69	1021	-
CD 13	0.8	34.5	f	L3	CDAI 412	B1	N	-	5-ASA	22.2	>1800	-

CD 14	0.6	36.6	m	L3	CDAI 363	B2	N	EEN	5-ASA, EEN	31.4	>1800	-
CD 15	1.0	28.0	m	L1	CDAI 229	B2	N	EEN	5-ASA	3.02	512	-
Combo 1	3.0	23.0	m	L3	CDAI 240	B2+B3	Y	EEN	5-ASA, GCS	>200	>1800	R
Combo 2	2.2	26.2	m	L3	CDAI 243	B2+B3	Y	EEN	5-ASA, GCS	12.3	1205	R
Combo 3	1.0	16.7	m	L3	CDAI 138	B2+B3	N	EEN	5-ASA	5.6	960	R
Combo 4	0.8	29.9	m	L1	CDAI 213	B2+B3	N	EEN	5-ASA	1.2	580	R
Combo 5	1.3	26.3	m	L1	CDAI 283	B2+B3	N	EEN	5-ASA, GCS	3.1	562	R
Combo 6	5.6	25.5	m	L3	CDAI 267	B2	Y	EEN	5-ASA	3.9	830	R
Combo 7	3.1	35.8	f	L1	CDAI 176	B2	N	EEN	5-ASA	3.02	170	R

CRP: C-reactive protein; m: male; f: femal; 5-ASA: aminosalicylates; GCS: corticosteroid; EEN: exclusive enteral nutrition; AZA: azathioprine; R: responsive; NR: non-responsive

Table S2. Differentially enriched microbes between UC patients and healthy donors.

No.	Bacterial genus	Bacterial phylum	Enriched group
1	Actinomyces	Actinobacteriota	UC-enriched
2	Actinomyces_bacterium	Actinobacteriota	UC-enriched
3	Bifidobacterium	Actinobacteriota	UC-enriched
4	Rothia_bacterium	Actinobacteriota	UC-enriched
5	Atopobium	Actinobacteriota	UC-enriched
6	Collinsella_bacterium	Actinobacteriota	UC-enriched
7	Adlercreutzia_bacterium	Actinobacteriota	UC-enriched
8	Eggerthella	Actinobacteriota	UC-enriched
9	Senegalimassilia_bacterium	Actinobacteriota	UC-enriched
10	Slackia_bacterium	Actinobacteriota	UC-enriched
11	Odoribacter	Bacteroidota	UC-enriched
12	Porphyromonas_bacterium	Bacteroidota	UC-enriched
13	Paraprevotella	Bacteroidota	UC-enriched
14	Prevotella_bacterium	Bacteroidota	UC-enriched
15	Desulfovibrio_bacterium	Desulfobacterota	UC-enriched
16	Erysipelatoclostridium	Firmicutes	UC-enriched
17	Holdemanella_bacterium	Firmicutes	UC-enriched
18	Solobacterium	Firmicutes	UC-enriched
19	[Clostridium]_innocuum_group	Firmicutes	UC-enriched
20	Granulicatella	Firmicutes	UC-enriched
21	Enterococcus	Firmicutes	UC-enriched
22	Lacticaseibacillus	Firmicutes	UC-enriched
23	Lactobacillus	Firmicutes	UC-enriched
24	Lactobacillus_bacterium	Firmicutes	UC-enriched
25	Ligilactobacillus	Firmicutes	UC-enriched
26	Limosilactobacillus	Firmicutes	UC-enriched
27	Streptococcus	Firmicutes	UC-enriched
28	Streptococcus_bacterium	Firmicutes	UC-enriched
29	Streptococcus_organism	Firmicutes	UC-enriched
30	Gemella_bacterium	Firmicutes	UC-enriched
31	Christensenellaceae_R-7_group	Firmicutes	UC-enriched
32	Eubacterium	Firmicutes	UC-enriched
33	Blautia_bacterium	Firmicutes	UC-enriched
34	Dorea_bacterium	Firmicutes	UC-enriched
35	[Eubacterium]_hallii_group_bacterium	Firmicutes	UC-enriched
36	[Ruminococcus]_gauvreauii_group_bacterium	Firmicutes	UC-enriched

37	Oscillibacter	Firmicutes	UC-enriched
38	Ruminococcaceae_unclassified	Firmicutes	UC-enriched
39	Ruminococcaceae_organism_unclassified	Firmicutes	UC-enriched
40	Family_XIII_AD3011_group_bacterium	Firmicutes	UC-enriched
41	Mogibacterium	Firmicutes	UC-enriched
42	Anaerococcus	Firmicutes	UC-enriched
43	Peptostreptococcus	Firmicutes	UC-enriched
44	Acidaminococcus	Firmicutes	UC-enriched
45	Phascolarctobacterium	Firmicutes	UC-enriched
46	TM7x_bacterium	Patescibacteria	UC-enriched
47	Saccharimonadaceae_bacterium_unclassified	Patescibacteria	UC-enriched
48	Comamonas	Proteobacteria	UC-enriched
49	Eikenella	Proteobacteria	UC-enriched
50	Sutterella	Proteobacteria	UC-enriched
51	Sutterella_bacterium	Proteobacteria	UC-enriched
52	Escherichia-Shigella	Proteobacteria	UC-enriched
53	Klebsiella	Proteobacteria	UC-enriched
54	Pluralibacter	Proteobacteria	UC-enriched
55	Morganella_bacterium	Proteobacteria	UC-enriched
56	Acinetobacter	Proteobacteria	UC-enriched
57	Pseudomonas	Proteobacteria	UC-enriched
58	Pyramidobacter	Synergistota	UC-enriched
59	Akkermansia_bacterium	Verrucomicrobiota	UC-enriched
60	Parabacteroides	Bacteroidota	UC-depeleted
61	Lachnoclostridium_bacterium	Firmicutes	UC-depeleted
62	Lachnoclostridium_organism	Firmicutes	UC-depeleted
63	Lachnospira_bacterium	Firmicutes	UC-depeleted
64	Lachnospira_organism	Firmicutes	UC-depeleted
65	Lachnospiraceae_NK4A136_group_bacterium	Firmicutes	UC-depeleted
66	Lachnospiraceae_UCG-004	Firmicutes	UC-depeleted
67	Lachnospiraceae_UCG-004_bacterium	Firmicutes	UC-depeleted
68	Roseburia_bacterium	Firmicutes	UC-depeleted
69	Lachnospiraceae_bacterium_unclassified	Firmicutes	UC-depeleted
70	Butyricicoccus_bacterium	Firmicutes	UC-depeleted
71	Ruminococcus_bacterium	Firmicutes	UC-depeleted

Table S3. Differentially enriched microbes between high- and low-contributed donors.

No.	Bacterial genus	Bacterial phylum	Enriched group
1	Actinomyces_bacterium	Actinobacteriota	Low-contributed donors
2	Prevotella_9_bacterium	Bacteroidota	Low-contributed donors
3	Erysipelotrichaceae_UCG-003	Firmicutes	Low-contributed donors
4	Anaerostipes_bacterium	Firmicutes	Low-contributed donors
5	Lachnospiraceae_ND3007_group_bacterium	Firmicutes	Low-contributed donors
6	Lachnospiraceae_UCG-003_bacterium	Firmicutes	Low-contributed donors
7	[Ruminococcus]_torques_group_bacterium	Firmicutes	Low-contributed donors
8	Megamonas_bacterium	Firmicutes	Low-contributed donors
9	Comamonas	Proteobacteria	Low-contributed donors
10	Bacteroides_bacterium	Bacteroidota	High-contributed donors
11	Bacteroides_organism	Bacteroidota	High-contributed donors
12	Bilophila	Desulfobacterota	High-contributed donors
13	Christensenellaceae_R-7_group_bacterium	Firmicutes	High-contributed donors
14	Colidextribacter	Firmicutes	High-contributed donors
15	Subdoligranulum_bacterium	Firmicutes	High-contributed donors

Table S4. Differentially enriched metabolites between UC patients and healthy donors.

Serial number	Metabolites	Class	VIP value	FDR-corrected p values
1	L_Lactic acid	Organic acids	2.2689	<0.01
2	3_Indolepropionic acid	Indoles	2.2391	0.447
3	Phenyllactic acid	Benzenoids	2.1545	<0.01
4	3,4_Dihydroxyhydrocinnamic acid	Organic acids	2.0503	0.430
5	Glycohyodeoxycholic acid	Bile acids	1.8789	0.413
6	2_Phenylpropionate	Phenylpropanoic acids	1.8468	0.447
7	Docosapentaenoic acid DPA	Fatty acids	1.8095	<0.01
8	Ketoleucine	Organic acids	1.7713	<0.01
9	2_Hydroxybutyric acid	Organic acids	1.7573	<0.01
10	Isocitric acid	Organic acids	1.7406	0.443
11	Glycohyocholic acid	Bile acids	1.7307	0.856
12	Adrenic acid	Fatty acids	1.5754	<0.01
13	L_Isoleucine	Amino acids	1.5268	<0.01
14	L_Leucine	Amino acids	1.5249	<0.01
15	m_Aminobenzoic acid	Benzenoids	1.5239	<0.01
16	Glycoursodeoxycholic acid	Bile acids	1.5089	0.266
17	Citrulline	Amino acids	1.4694	<0.01
18	Ursocholic Acid	Bile acids	1.4458	0.466
19	Phenylpyruvic acid	Benzenoids	1.4386	<0.01
20	Alpha_ketoisovaleric acid	Organic acids	1.4249	<0.01
21	L_Alanine	Amino acids	1.424	<0.01
22	Omega_muricholic acid	Bile acids	1.4162	0.638
23	L_Valine	Amino acids	1.4132	<0.01
24	Valeric acid	Fatty acids	1.4043	0.365
25	Hydrocinnamic acid	Phenylpropanoic acids	1.3921	0.413
26	isolithocholic acid	Bile acids	1.3859	0.830
27	Arachidonic acid	Fatty acids	1.3628	<0.01
28	Beta_Muricholic Acid	Bile acids	1.3454	0.430
29	Isovaleric acid	Fatty acids	1.3378	0.856
30	Taurochenodeoxycholic acid	Bile acids	1.326	0.266
31	L_Lysine	Amino acids	1.3199	<0.01
32	3_Hydroxyisovaleric acid	Fatty acids	1.318	0.275
33	Tauroursodeoxycholic acid	Bile acids	1.3133	0.413
34	3_Methyl_2_oxovaleric acid	Organic acids	1.2929	<0.01
35	Glycine	Amino acids	1.2189	<0.01

36	7_Ketolithocholic Acid	Bile acids	1.204	0.430
37	Isobutyric acid	Fatty acids	1.2004	0.413
38	L_Serine	Amino acids	1.178	<0.01
39	3_Dehydrocholic acid	Bile acids	1.1647	0.266
40	Ursodeoxycholic acid	Bile acids	1.1589	0.254
41	2_Hydroxycaproic acid	Fatty acids	1.1569	0.466
42	L_Tryptophan	Amino acids	1.1458	<0.01
43	Hydroxyphenyllactic acid	Phenylpropanoic acids	1.1426	<0.01
44	L_Tyrosine	Amino acids	1.1073	<0.01
45	Hyodeoxycholic acid	Bile acids	1.082	0.299
46	Decanoic acid	Fatty acids	1.0747	0.013
47	L_threonine	Amino acids	1.0508	<0.01
48	Chenodeoxycholic acid	Bile acids	1.0487	0.254
49	Glycochenodeoxycholic acid	Bile acids	1.0424	0.141
50	L_Cystine	Amino acids	1.034	<0.01
51	Glycolithocholic acid	Bile acids	1.0187	0.973
52	Butyric acid	Fatty acids	1.0065	0.299
53	L_Methionine	Amino acids	1.0059	<0.01
54	Ornithine	Amino acids	0.99868	<0.01
55	Nor Cholic acid	Bile acids	0.99379	0.299
56	Hyocholic acid	Bile acids	0.98614	0.051
57	Heptanoic acid	Fatty acids	0.98198	0.181
58	Taurocholic acid	Bile acids	0.97424	0.051
59	Citric acid	Organic acids	0.96851	0.070
60	Glycolic acid	Organic acids	0.96583	0.027
61	L_Histidine	Amino acids	0.94501	<0.01
62	Glycocholic acid	Bile acids	0.94434	0.086
63	L_Proline	Amino acids	0.93369	0.027
64	Nordeoxycholic acid	Bile acids	0.92945	0.458
65	7_Dehydrocholic acid	Bile acids	0.92233	0.299

Table S5. FMT response-associated metabolites in UC patients.

Serial number	Metabolites	Class
1	L_Lysine	Amino acids
2	L_Histidine	Amino acids
3	Ornithine	Amino acids
4	L_Cystine	Amino acids
5	L_Alanine	Amino acids
6	L_threonine	Amino acids
7	L_Tyrosine	Amino acids
8	Glycine	Amino acids
9	Citrulline	Amino acids
10	L_Proline	Amino acids
11	L_Valine	Amino acids
12	L_Methionine	Amino acids
13	L_Isoleucine	Amino acids
14	L_Leucine	Amino acids
15	L_Tryptophan	Amino acids
16	Phenylpyruvic acid	Benzenoids
17	Phenyllactic acid	Benzenoids
18	Omega_muricholic acid	Bile acids
19	Glycohyodeoxycholic acid	Bile acids
20	Ursodeoxycholic acid	Bile acids
21	Hyodeoxycholic acid	Bile acids
22	isolithocholic acid	Bile acids
23	Nor Cholic acid	Bile acids
24	2_Hydroxycaproic acid	Fatty acids
25	3_Hydroxyisovaleric acid	Fatty acids
26	Butyric acid	Fatty acids
27	Isobutyric acid	Fatty acids
28	Isovaleric acid	Fatty acids
29	Valeric acid	Fatty acids
30	Heptanoic acid	Fatty acids
31	Decanoic acid	Fatty acids
32	Arachidonic acid	Fatty acids
33	Adrenic acid	Fatty acids
34	3_Indolepropionic acid	Indoles
35	L_Lactic acid	Organic acids

36	Alpha_ketoisovaleric acid	Organic acids
37	Glycolic acid	Organic acids
38	2_Hydroxybutyric acid	Organic acids
39	Hydroxyphenyllactic acid	Phenylpropanoic acids
40	2_Phenylpropionate	Phenylpropanoic acids
41	Hydrocinnamic acid	Phenylpropanoic acids

Table S6. Differentially enriched microbes between CD patients and healthy donors.

Serial number	Bacterial genus	Bacterial phylum	Enriched group
1	Actinomyces	Actinobacteriota	CD-enriched
2	Actinomyces_bacterium	Actinobacteriota	CD-enriched
3	Bifidobacterium	Actinobacteriota	CD-enriched
4	Rothia_bacterium	Actinobacteriota	CD-enriched
5	Atopobium	Actinobacteriota	CD-enriched
6	Collinsella_bacterium	Actinobacteriota	CD-enriched
7	Eggerthella	Actinobacteriota	CD-enriched
8	Senegalimassilia_bacterium	Actinobacteriota	CD-enriched
9	Porphyromonas_bacterium	Bacteroidota	CD-enriched
10	Prevotella	Bacteroidota	CD-enriched
11	Prevotella_7_organism	Bacteroidota	CD-enriched
12	Prevotella_bacterium	Bacteroidota	CD-enriched
13	Erysipelatoclostridium	Firmicutes	CD-enriched
14	Solobacterium	Firmicutes	CD-enriched
15	[Clostridium]_innocuum_group	Firmicutes	CD-enriched
16	Abiotrophia_bacterium	Firmicutes	CD-enriched
17	Granulicatella	Firmicutes	CD-enriched
18	Enterococcus	Firmicutes	CD-enriched
19	Lactiplantibacillus	Firmicutes	CD-enriched
20	Lactobacillus	Firmicutes	CD-enriched
21	Lactobacillus_bacterium	Firmicutes	CD-enriched
22	Ligilactobacillus	Firmicutes	CD-enriched
23	Limosilactobacillus	Firmicutes	CD-enriched
24	Streptococcus	Firmicutes	CD-enriched
25	Streptococcus_bacterium	Firmicutes	CD-enriched
26	Streptococcus_organism	Firmicutes	CD-enriched
27	Gemella_bacterium	Firmicutes	CD-enriched
28	Clostridium_sensu_stricto_1	Firmicutes	CD-enriched
29	Eubacterium	Firmicutes	CD-enriched
30	Anaerostipes	Firmicutes	CD-enriched
31	UBA1819_organism	Firmicutes	CD-enriched
32	Family_XIII_AD3011_group_bacterium	Firmicutes	CD-enriched
33	Mogibacterium	Firmicutes	CD-enriched
34	Anaerococcus	Firmicutes	CD-enriched
35	Peptostreptococcus	Firmicutes	CD-enriched

36	Peptostreptococcus_bacterium	Firmicutes	CD-enriched
37	Acidaminococcus	Firmicutes	CD-enriched
38	Phascolarctobacterium	Firmicutes	CD-enriched
39	Anaeroglobus	Firmicutes	CD-enriched
40	Dialister_organism	Firmicutes	CD-enriched
41	Megasphaera	Firmicutes	CD-enriched
42	Megasphaera_bacterium	Firmicutes	CD-enriched
43	Veillonella_bacterium	Firmicutes	CD-enriched
44	Fusobacterium	Fusobacteriota	CD-enriched
45	TM7x_bacterium	Patescibacteria	CD-enriched
46	Saccharimonadaceae_bacterium_unclassified	Patescibacteria	CD-enriched
47	Acidovorax	Proteobacteria	CD-enriched
48	Comamonas	Proteobacteria	CD-enriched
49	Eikenella	Proteobacteria	CD-enriched
50	Neisseria_bacterium	Proteobacteria	CD-enriched
51	Sutterella	Proteobacteria	CD-enriched
52	Escherichia-Shigella	Proteobacteria	CD-enriched
53	Escherichia-Shigella_bacterium	Proteobacteria	CD-enriched
54	Klebsiella	Proteobacteria	CD-enriched
55	Morganella_bacterium	Proteobacteria	CD-enriched
56	Pseudomonas	Proteobacteria	CD-enriched
57	Akkermansia_bacterium	Verrucomicrobiota	CD-enriched
58	Bacteroides_organism	Bacteroidota	CD-depleted
59	Prevotella_9_bacterium	Bacteroidota	CD-depleted
60	Christensenellaceae_R-7_group_bacterium	Firmicutes	CD-depleted
61	CAG-56	Firmicutes	CD-depleted
62	Dorea_bacterium	Firmicutes	CD-depleted
63	Lachnoclostridium_bacterium	Firmicutes	CD-depleted
64	Lachnospira_bacterium	Firmicutes	CD-depleted
65	Lachnospira_organism	Firmicutes	CD-depleted
66	Lachnospiraceae_ND3007_group_bacterium	Firmicutes	CD-depleted
67	Lachnospiraceae_NK4A136_group_bacterium	Firmicutes	CD-depleted
68	Lachnospiraceae_NK4A136_group_organism	Firmicutes	CD-depleted
69	Lachnospiraceae_UCG-004_bacterium	Firmicutes	CD-depleted
70	Roseburia_bacterium	Firmicutes	CD-depleted
71	[Eubacterium]_hallii_group_bacterium	Firmicutes	CD-depleted
72	[Ruminococcus]_torques_group	Firmicutes	CD-depleted
73	Lachnospiraceae_bacterium_unclassified	Firmicutes	CD-depleted
74	Monoglobus_bacterium	Firmicutes	CD-depleted
75	Butyricicoccus_bacterium	Firmicutes	CD-depleted

76	Colidextribacter	Firmicutes	CD-depleted
77	UCG-003_bacterium	Firmicutes	CD-depleted
78	Faecalibacterium_bacterium	Firmicutes	CD-depleted
79	Ruminococcus_bacterium	Firmicutes	CD-depleted
80	Subdoligranulum_bacterium	Firmicutes	CD-depleted
81	[Eubacterium]_coprostanoligenes_group_bacterium_unclassified	Firmicutes	CD-depleted
82	Romboutsia_bacterium	Firmicutes	CD-depleted
83	Parasutterella_bacterium	Proteobacteria	CD-depleted

Table S7. Differentially enriched metabolites between CD patients and healthy donors.

Serial number	Metabolites	Class	VIP value	FDR-corrected p values
1	isolithocholic acid	Bile acids	2.7383	<0.01
2	Deoxycholic acid	Bile acids	2.4187	<0.01
3	Glycodeoxycholic acid	Bile acids	2.1705	0.050
4	3_Indolepropionic acid	Indoles	2.0913	0.103
5	Lithocholic acid	Bile acids	2.0445	0.576
6	Taurolithocholic acid	Bile acids	1.9162	0.103
7	Glycolic acid	Organic acids	1.9131	0.147
8	Hyodeoxycholic acid	Bile acids	1.79	0.624
9	Omega_muricholic acid	Bile acids	1.7815	0.624
10	2_Phenylpropionate	Phenylpropanoic acids	1.7728	0.126
11	3,4_Dihydroxyhydrocinnamic acid	Organic acids	1.7678	0.624
12	Glycolithocholic acid	Bile acids	1.6775	0.147
13	Valeric acid	Fatty acids	1.6773	0.459
14	Hyocholic acid	Bile acids	1.5614	0.685
15	Itaconic acid	Organic acids	1.5573	0.136
16	Butyric acid	Fatty acids	1.4801	0.087
17	L_Isoleucine	Amino acids	1.3842	<0.01
18	Nordeoxycholic acid	Bile acids	1.3679	0.971
19	L_Valine	Amino acids	1.3659	<0.01
20	Ursocholic Acid	Bile acids	1.3635	1
21	Methylglutaric acid	Fatty acids	1.3228	0.576
22	L_Leucine	Amino acids	1.3198	<0.01
23	L_Proline	Amino acids	1.2876	<0.01
24	Beta_Muricholic Acid	Bile acids	1.2648	0.396
25	Glycine	Amino acids	1.2508	<0.01
26	Ketoleucine	Organic acids	1.2484	<0.01
27	L_Alanine	Amino acids	1.2395	<0.01
28	L_Lactic acid	Organic acids	1.2356	0.012
29	L_Histidine	Amino acids	1.2204	<0.01
30	Picolinic acid	Pyridines	1.2011	0.941
31	Glutaric acid	Organic acids	1.1936	0.576
32	Phenylpyruvic acid	Benzenoids	1.1832	<0.01
33	Phenyllactic acid	Benzenoids	1.1785	<0.01
34	Glycocholic acid	Bile acids	1.1725	<0.01

35	Hydrocinnamic acid	Phenylpropanoic acids	1.1552	0.126
36	L_Alpha_aminobutyric acid	Amino acids	1.1536	<0.01
37	Docosapentaenoic acid DPA	Fatty acids	1.1486	0.031
38	L_Serine	Amino acids	1.1325	<0.01
39	L_threonine	Amino acids	1.1203	<0.01
40	Glycochenodeoxycholic acid	Bile acids	1.12	<0.01
41	L_Tryptophan	Amino acids	1.1178	<0.01
42	3_Methyl_2_oxovaleric acid	Organic acids	1.1169	<0.01
43	L_Tyrosine	Amino acids	1.1137	<0.01
44	Methylsuccinic acid	Fatty acids	1.1106	0.576
45	2_Hydroxycaproic acid	Fatty acids	1.1044	0.685
46	Decanoic acid	Fatty acids	1.0681	0.023
47	3_Hydroxyisovaleric acid	Fatty acids	1.0404	0.087
48	L_Glutamine	Amino acids	1.037	<0.01
49	2_Hydroxybutyric acid	Organic acids	1.0232	<0.01
50	Pyroglutamic acid	Amino acids	0.99038	<0.01
51	L_Cystine	Amino acids	0.974	<0.01
52	D_Xylose	Carbohydrates	0.96367	0.087
53	Ursodeoxycholic acid	Bile acids	0.94237	0.103
54	Palmitoleic acid	Fatty acids	0.94027	<0.01
55	Fumaric acid	Organic acids	0.93706	<0.01
56	L_Methionine	Amino acids	0.93038	<0.01
57	Arachidonic acid	Fatty acids	0.92047	<0.01
58	Citrulline	Amino acids	0.90995	<0.01

Table S8. FMT response-associated metabolites in CD patients.

Serial number	Metabolites	Class
1	L_Histidine	Amino acids
2	L_Glutamine	Amino acids
3	L_Cystine	Amino acids
4	L_Alanine	Amino acids
5	L_Serine	Amino acids
6	L_threonine	Amino acids
7	L_Tyrosine	Amino acids
8	Glycine	Amino acids
9	Citrulline	Amino acids
10	L_Alpha_aminobutyric acid	Amino acids
11	L_Proline	Amino acids
12	L_Valine	Amino acids
13	Pyroglutamic acid	Amino acids
14	L_Methionine	Amino acids
15	L_Isoleucine	Amino acids
16	L_Leucine	Amino acids
17	L_Tryptophan	Amino acids
18	Phenylpyruvic acid	Benzenoids
19	Phenyllactic acid	Benzenoids
20	Beta_Muricholic Acid	Bile acids
21	Ursodeoxycholic acid	Bile acids
22	Hyodeoxycholic acid	Bile acids
23	Hyocholic acid	Bile acids
24	Glycocholic acid	Bile acids
25	isolithocholic acid	Bile acids
26	Glycochenodeoxycholic acid	Bile acids
27	Lithocholic acid	Bile acids
28	Deoxycholic acid	Bile acids
29	Glycolithocholic acid	Bile acids
30	Nordeoxycholic acid	Bile acids
31	D_Xylose	Carbohydrates
32	2_Hydroxycaproic acid	Fatty acids
33	3_Hydroxyisovaleric acid	Fatty acids
34	Butyric acid	Fatty acids
35	Valeric acid	Fatty acids
36	Methylsuccinic acid	Fatty acids
37	Methylglutaric acid	Fatty acids

38	Decanoic acid	Fatty acids
39	Palmitoleic acid	Fatty acids
40	Docosapentaenoic acid DPA	Fatty acids
41	3_Indolepropionic acid	Indoles
42	L_Lactic acid	Organic acids
43	Fumaric acid	Organic acids
44	Glutaric acid	Organic acids
45	Ketoleucine	Organic acids
46	3_Methyl_2_oxovaleric acid	Organic acids
47	Glycolic acid	Organic acids
48	2_Hydroxybutyric acid	Organic acids
49	Hydrocinnamic acid	Phenylpropanoic acids
50	Picolinic acid	Pyridines

Table S9. Differentially enriched microbes between IFX treated CD patients and healthy donors.

Serial number	Bacterial genus	Bacterial phylum	Enriched group
1	Sutterella	Proteobacteria	CD-IFX enriched
2	Prevotella_bacterium	Bacteroidota	CD-IFX enriched
3	Lachnoclostridium	Firmicutes	CD-IFX enriched
4	Clostridium_innocuum_group	Firmicutes	CD-IFX enriched
5	Pseudomonas	Proteobacteria	CD-IFX enriched
6	Enterococcus	Firmicutes	CD-IFX enriched
7	Clostridioides	Firmicutes	CD-IFX enriched
8	Atopobium	Actinobacteriota	CD-IFX enriched
9	Akkermansia_bacterium	Verrucomicrobiota	CD-IFX enriched
10	Bifidobacterium	Actinobacteriota	CD-IFX enriched
11	Hungatella	Firmicutes	CD-IFX enriched
12	Erysipelatoclostridium	Firmicutes	CD-IFX enriched
13	Lactobacillus	Firmicutes	CD-IFX enriched
14	Anaerostipes	Firmicutes	CD-IFX enriched
15	Ruminococcus_gnavus_group_bacterium	Firmicutes	CD-IFX enriched
16	Ligilactobacillus	Firmicutes	CD-IFX enriched
17	Escherichia-Shigella_bacterium	Proteobacteria	CD-IFX enriched
18	Morganella	Proteobacteria	CD-IFX enriched
19	Escherichia-Shigella	Proteobacteria	CD-IFX enriched
20	Actinomyces	Actinobacteriota	CD-IFX enriched
21	Veillonella_bacterium	Firmicutes	CD-IFX enriched
22	Actinomyces_bacterium	Actinobacteriota	CD-IFX enriched
23	DTU089_organism	Firmicutes	CD-IFX enriched
24	Raoultella	Proteobacteria	CD-IFX enriched
25	Intestinibacter	Firmicutes	CD-IFX enriched
26	Flavonifractor_bacterium	Firmicutes	CD-IFX enriched
27	Eggerthella_Gordonibacter_sp.	Actinobacteriota	CD-IFX enriched
28	TM7x_bacterium	Patescibacteria	CD-IFX enriched
29	Comamonas	Proteobacteria	CD-IFX enriched
30	Lactococcus	Firmicutes	CD-IFX enriched
31	Streptococcus	Firmicutes	CD-IFX enriched
32	Eikenella	Proteobacteria	CD-IFX enriched
33	Aeromonas	Proteobacteria	CD-IFX enriched
34	Lacticaseibacillus	Firmicutes	CD-IFX enriched
35	Klebsiella	Proteobacteria	CD-IFX enriched
36	Ruminococcus_gnavus_group_organism	Firmicutes	CD-IFX enriched
37	Fusobacterium	Fusobacteriota	CD-IFX enriched

38	Providencia	Proteobacteria	CD-IFX enriched
39	Ruminococcaceae_unclassified	Firmicutes	CD-IFX enriched
40	Weissella	Firmicutes	CD-IFX enriched
41	UBA1819_organism	Firmicutes	CD-IFX enriched
42	Parabacteroides	Bacteroidota	CD-IFX enriched
43	Pluralibacter	Proteobacteria	CD-IFX enriched
44	Solobacterium	Firmicutes	CD-IFX enriched
45	Acidaminococcus	Firmicutes	CD-IFX enriched
46	Faecalibacterium_bacterium	Firmicutes	CD-IFX depleted
47	Colidextribacter	Firmicutes	CD-IFX depleted
48	Lachnospiraceae_bacterium_unclassified	Firmicutes	CD-IFX depleted
49	Subdoligranulum_bacterium	Firmicutes	CD-IFX depleted
50	Lachnoclostridium_bacterium	Firmicutes	CD-IFX depleted
51	UCG-003_bacterium	Firmicutes	CD-IFX depleted
52	Roseburia_bacterium	Firmicutes	CD-IFX depleted
53	Ruminococcus_bacterium	Firmicutes	CD-IFX depleted
54	Christensenellaceae_R-7_group_bacterium	Firmicutes	CD-IFX depleted
55	Lachnospiraceae_UCG-004_bacterium	Firmicutes	CD-IFX depleted
56	Lachnospira_organism	Firmicutes	CD-IFX depleted

Table S10. Differentially enriched microbes between IFX-FMT treated CD patients and healthy donors.

Serial number	Bacterial genus	Bacterial phylum	Enriched group
1	Eggerthella	Actinobacteriota	IFX-FMT enriched
2	Atopobium	Actinobacteriota	IFX-FMT enriched
3	Collinsella_bacterium	Actinobacteriota	IFX-FMT enriched
4	Rothia_bacterium	Actinobacteriota	IFX-FMT enriched
5	Paraprevotella_bacterium	Bacteroidota	IFX-FMT enriched
6	TM7x_bacterium	Patescibacteria	IFX-FMT enriched
7	Enterococcus	Firmicutes	IFX-FMT enriched
8	Rhodococcus	Actinobacteriota	IFX-FMT enriched
9	Akkermansia_bacterium	Verrucomicrobiota	IFX-FMT enriched
10	Anaerostipes	Firmicutes	IFX-FMT enriched
11	Bifidobacterium	Actinobacteriota	IFX-FMT enriched
12	Erysipelatoclostridium	Firmicutes	IFX-FMT enriched
13	Actinomyces_bacterium	Actinobacteriota	IFX-FMT enriched
14	Negativibacillus_bacterium	Firmicutes	IFX-FMT enriched
15	Saccharimonadaceae_bacterium_unclassified	Patescibacteria	IFX-FMT enriched
16	Intestinibacter	Firmicutes	IFX-FMT enriched
17	Clostridioides	Firmicutes	IFX-FMT enriched
18	Sutterella	Proteobacteria	IFX-FMT enriched
19	Ruminococcaceae_bacterium_unclassified	Firmicutes	IFX-FMT enriched
20	Family_XIII_AD3011_group_bacterium	Firmicutes	IFX-FMT enriched
21	Clostridium_innocuum_group	Firmicutes	IFX-FMT enriched
22	Anaerococcus	Firmicutes	IFX-FMT enriched
23	UBA1819_organism	Firmicutes	IFX-FMT enriched
24	Flavonifractor_bacterium	Firmicutes	IFX-FMT enriched
25	Mogibacterium	Firmicutes	IFX-FMT enriched
26	Adlercreutzia_bacterium	Actinobacteriota	IFX-FMT enriched
27	Ruminococcus_gnavus_group_bacterium	Firmicutes	IFX-FMT enriched
28	Blautia_bacterium	Firmicutes	IFX-FMT enriched
29	Streptococcus	Firmicutes	IFX-FMT enriched
30	DTU089_organism	Firmicutes	IFX-FMT enriched
31	Ruminococcus_gnavus_group	Firmicutes	IFX-FMT enriched
32	Ruminococcaceae_organism_unclassified	Firmicutes	IFX-FMT enriched

33	Paraprevotella	Bacteroidota	IFX-FMT enriched
34	Phascolarctobacterium	Firmicutes	IFX-FMT enriched
35	Family_XIII_AD3011_group_organism	Firmicutes	IFX-FMT enriched
36	Parabacteroides_bacterium	Bacteroidota	IFX-FMT enriched
37	Eubacterium	Firmicutes	IFX-FMT enriched
38	Phascolarctobacterium_bacterium	Firmicutes	IFX-FMT enriched
39	Klebsiella	Proteobacteria	IFX-FMT enriched
40	Senegalimassilia_bacterium	Actinobacteriota	IFX-FMT enriched
41	Prevotella_bacterium	Bacteroidota	IFX-FMT enriched
42	Eubacterium_hallii_group_bacterium	Firmicutes	IFX-FMT enriched
43	Ruminococcaceae_unclassified	Firmicutes	IFX-FMT enriched
44	Christensenellaceae_R-7_group	Firmicutes	IFX-FMT enriched
45	Faecalitalea_bacterium	Firmicutes	IFX-FMT enriched
46	Solobacterium	Firmicutes	IFX-FMT enriched
47	Holdemanella_bacterium	Firmicutes	IFX-FMT enriched
48	Odoribacter	Bacteroidota	IFX-FMT enriched
49	Actinomyces	Actinobacteriota	IFX-FMT enriched
50	Desulfovibrio_bacterium	Desulfobacterota	IFX-FMT enriched
51	Oscillibacter	Firmicutes	IFX-FMT enriched
52	Streptococcus_organism	Firmicutes	IFX-FMT enriched
53	Tyzzerella_bacterium	Firmicutes	IFX-FMT enriched
54	Christensenellaceae_R-7_group_bacterium	Firmicutes	IFX-FMT enriched
55	Porphyromonas_bacterium	Bacteroidota	IFX-FMT enriched
56	Clostridium_sensu_stricto_1	Firmicutes	IFX-FMT enriched
57	Incertae_Sedis_organism	Firmicutes	IFX-FMT enriched
58	Dorea_bacterium	Firmicutes	IFX-FMT enriched
59	Morganella_bacterium	Proteobacteria	IFX-FMT enriched
60	Romboutsia_bacterium	Firmicutes	IFX-FMT enriched
61	Desulfovibrio	Desulfobacterota	IFX-FMT enriched
62	Lachnoclostridium_bacterium	Firmicutes	IFX-FMT depleted
63	Faecalibacterium_bacterium	Firmicutes	IFX-FMT depleted
64	Butyricicoccus_bacterium	Firmicutes	IFX-FMT depleted
65	Lachnospiraceae_bacterium_unclassified	Firmicutes	IFX-FMT depleted
66	Bacteroides_organism	Bacteroidota	IFX-FMT depleted
67	Roseburia_bacterium	Firmicutes	IFX-FMT depleted
68	Lachnospiraceae_UCG-004_bacterium	Firmicutes	IFX-FMT depleted

Table S11. Differentially enriched metabolites between IFX treated CD patients and healthy donors.

Serial number	Metabolites	Class
1	Valeric acid	Fatty acids
2	Chenodeoxycholic acid	Bile acids
3	Docosapentaenoic acid DPA	Fatty acids
4	3,4_Dihydroxyhydrocinnamic acid	Organic acids
5	p_Hydroxyphenylacetic acid	Benzenoids
6	Beta_Alanine	Amino acids
7	L_Isoleucine	Amino acids
8	N_Phenylacetylphenylalanine	Amino acids
9	Adrenic acid	Fatty acids
10	Hydrocinnamic acid	Phenylpropanoic acids
11	D_Xylose	Carbohydrates
12	Indoleacetic acid	Indoles
13	2_Phenylpropionate	Phenylpropanoic acids
14	L_Leucine	Amino acids
15	2_Hydroxy_3_methylbutyric acid	Fatty acids
16	L_Serine	Amino acids
17	Itaconic acid	Organic acids
18	L_Valine	Amino acids
19	Adipic acid	Fatty acids
20	Alpha_Linolenic acid	Fatty acids
21	3_Indolepropionic acid	Indoles
22	Ethylmethylacetic acid	Fatty acids
23	L_Histidine	Amino acids
24	3_Methyladipic acid	Fatty acids
25	2_Hydroxybutyric acid	Organic acids
26	L_threonine	Amino acids
27	Ketoleucine	Organic acids
28	D_Glucose	Carbohydrates
29	3_Methyl_2_oxovaleric acid	Organic acids
30	L_Tryptophan	Amino acids
31	L_Glutamine	Amino acids
32	L_Alanine	Amino acids
33	Glycolithocholic acid	Bile acids
34	L_Cystine	Amino acids
35	4_Hydroxybenzoic acid	Benzenoids
36	L_Tyrosine	Amino acids

37	Nor Cholic acid	Bile acids
38	L_Lysine	Amino acids
39	Isovaleric acid	Fatty acids
40	Octanoic acid	Fatty acids
41	Eicosapentaenoic acid EPA	Fatty acids
42	Butyric acid	Fatty acids
43	Glyceric acid	Carbohydrates
44	Citramalic acid	Fatty acids
45	Phenylacetic acid	Benzenoids
46	Taurolithocholic acid	Bile acids
47	L_Acetylcarnitine	Carnitines
48	Docosahexaenoic acid DHA	Fatty acids
49	Decanoic acid	Fatty acids
50	Homovanillic acid	Benzenoids
51	Methylcysteine	Amino acids
52	Benzoic acid	Benzenoids
53	Ornithine	Amino acids
54	Glycine	Amino acids
55	Isobutyric acid	Fatty acids
56	m_Aminobenzoic acid	Benzenoids

Table S12. Differentially enriched metabolites between IFX-FMT treated CD patients and healthy donors.

Serial number	Metabolites	Class
1	Threonic acid	Organic acids
2	2_Hydroxycaproic acid	Fatty acids
3	Isocitric acid	Organic acids
4	Glycohyocholic acid	Bile acids
5	Glycohyodeoxycholic acid	Bile acids
6	Taurochenodeoxycholic acid	Bile acids
7	Hippuric acid	Benzenoids
8	Docosapentaenoic acid DPA	Fatty acids
9	Taurocholic acid	Bile acids
10	Tauroursodeoxycholic acid	Bile acids
11	Citric acid	Organic acids
12	3_Hydroxybutyric acid	Organic acids
13	4_Aminohippuric acid	Benzenoids
14	Taurolithocholic acid	Bile acids
15	3_Hydroxyisovaleric acid	Fatty acids
16	3,4_Dihydroxyhydrocinnamic acid	Organic acids
17	Glycolithocholic acid	Bile acids
18	Eicosapentaenoic acid EPA	Fatty acids
19	Nordeoxycholic acid	Bile acids
20	Taurodeoxycholic acid	Bile acids
21	Decanoic acid	Fatty acids
22	D_Xylose	Carbohydrates
23	N_Phenylacetylphenylalanine	Amino acids
24	Glyceric acid	Carbohydrates
25	Ursocholic Acid	Bile acids
26	Glycolic acid	Organic acids
27	Docosahexaenoic acid DHA	Fatty acids
28	Propionylcarnitine	Carnitines
29	Adrenic acid	Fatty acids
30	Cis_and trans_Cinnamic acid	Phenylpropanoic acids
31	L_Asparagine	Amino acids
32	Alpha_Linolenic acid	Fatty acids
33	Ketoleucine	Organic acids
34	Homovanillic acid	Benzenoids
35	Dimethylglycine	Amino acids
36	Ethylmethylacetic acid	Fatty acids

37	L_Acetylcarnitine	Carnitines
38	D_Fructose	Carbohydrates
39	D_Glucose	Carbohydrates
40	Adipic acid	Fatty acids
41	Glycochenodeoxycholic acid	Bile acids
42	Dodecanoic acid	Fatty acids
43	Glycodeoxycholic acid	Bile acids
44	Glycocholic acid	Bile acids
45	4_Hydroxyphenylpyruvic acid	Benzenoids
46	Myristoleic acid	Fatty acids
47	Creatine	Amino acids

Reference:

1. Yang Y, Misra BB, Liang L, et al. Integrated microbiome and metabolome analysis reveals a novel interplay between commensal bacteria and metabolites in colorectal cancer. Theranostics 2019;9:4101-4114.
2. Sargun A, Gerner RR, Raffatellu M, et al. Harnessing Iron Acquisition Machinery to Target Enterobacteriaceae. J Infect Dis 2021;223:S307-s313.
3. Molloy MJ, Grainger JR, Bouladoux N, et al. Intraluminal containment of commensal outgrowth in the gut during infection-induced dysbiosis. Cell Host Microbe 2013;14:318-28.
4. Wu X, Pan S, Luo W, et al. Roseburia intestinalis-derived flagellin ameliorates colitis by targeting miR-223-3p-mediated activation of NLRP3 inflammasome and pyroptosis. Mol Med Rep 2020;22:2695-2704.
5. Shen Z, Zhu C, Quan Y, et al. Insights into Roseburia intestinalis which alleviates experimental colitis pathology by inducing anti-inflammatory responses. J Gastroenterol Hepatol 2018;33:1751-1760.
6. Zhu C, Song K, Shen Z, et al. Roseburia intestinalis inhibits interleukin-17 excretion and promotes regulatory T cells differentiation in colitis. Mol Med Rep 2018;17:7567-7574.
7. Tan B, Luo W, Shen Z, et al. Roseburia intestinalis inhibits oncostatin M and maintains tight junction integrity in a murine model of acute experimental colitis. Scand J Gastroenterol 2019;54:432-440.
8. Lloyd-Price J, Arze C, Ananthakrishnan AN, et al. Multi-omics of the gut microbial ecosystem in inflammatory bowel diseases. Nature 2019;569:655-662.
9. Aden K, Rehman A, Waschina S, et al. Metabolic Functions of Gut Microbes Associate With Efficacy of Tumor Necrosis Factor Antagonists in Patients With Inflammatory Bowel Diseases. Gastroenterology 2019;157:1279-1292.e11.
10. Shen ZH, Zhu CX, Quan YS, et al. Relationship between intestinal microbiota and ulcerative colitis: Mechanisms and clinical application of probiotics and fecal microbiota transplantation. World J Gastroenterol 2018;24:5-14.
11. Zhang R, Qin Q, Liu B, et al. TiO(2)-Assisted Laser Desorption/Ionization Mass Spectrometry for Rapid Profiling of Candidate Metabolite Biomarkers from Antimicrobial-Resistant Bacteria. Anal Chem 2018;90:3863-3870.
12. Xin Z, Zhai Z, Long H, et al. Metabolic Profiling by UPLC-Orbitrap-MS/MS of Liver from C57BL/6 Mice with DSS-Induced Inflammatory Bowel Disease. Mediators Inflamm 2020;2020:6020247.

13. Guo D, Yang J, Ling F, et al. Elemental Diet Enriched with Amino Acids Alleviates Mucosal Inflammatory Response and Prevents Colonic Epithelial Barrier Dysfunction in Mice with DSS-Induced Chronic Colitis. J Immunol Res 2020;2020:9430763.
14. Ward JBJ, Lajczak NK, Kelly OB, et al. Ursodeoxycholic acid and lithocholic acid exert anti-inflammatory actions in the colon. Am J Physiol Gastrointest Liver Physiol 2017;312:G550-g558.
15. Mroz MS, Lajczak NK, Goggins BJ, et al. The bile acids, deoxycholic acid and ursodeoxycholic acid, regulate colonic epithelial wound healing. Am J Physiol Gastrointest Liver Physiol 2018;314:G378-g387.
16. Estevinho MM, Rocha C, Correia L, et al. Features of Fecal and Colon Microbiomes Associate With Responses to Biologic Therapies for Inflammatory Bowel Diseases: A Systematic Review. Clin Gastroenterol Hepatol 2020;18:1054-1069.
17. Kim ES, Tarassishin L, Eisele C, et al. Longitudinal Changes in Fecal Calprotectin Levels Among Pregnant Women With and Without Inflammatory Bowel Disease and Their Babies. Gastroenterology 2021;160:1118-1130.e3.
18. Rengarajan S, Vivio EE, Parkes M, et al. Dynamic immunoglobulin responses to gut bacteria during inflammatory bowel disease. Gut Microbes 2020;11:405-420.
19. Duboc H, Rajca S, Rainteau D, et al. Connecting dysbiosis, bile-acid dysmetabolism and gut inflammation in inflammatory bowel diseases. Gut 2013;62:531-9.
20. Fitzpatrick LR, Jenabzadeh P. IBD and Bile Acid Absorption: Focus on Pre-clinical and Clinical Observations. Front Physiol 2020;11:564.
21. Hou RG, Fan L, Liu JJ, et al. Bile acid malabsorption is associated with diarrhea in acute phase of colitis. Can J Physiol Pharmacol 2018;96:1328-1336.
